# A novel chemotactic factor derived from the extracellular matrix protein decorin recruits mesenchymal stromal cells *in vitro* and *in vivo*

**DOI:** 10.1371/journal.pone.0235784

**Published:** 2020-07-13

**Authors:** Sandi Grainne Dempsey, Christopher Hamilton Miller, Julia Schueler, Robert W. F. Veale, Darren J. Day, Barnaby C. H. May

**Affiliations:** 1 Aroa Biosurgery Limited, Airport Oaks, Auckland, New Zealand; 2 School of Biological Sciences, Victoria University of Wellington, Wellington, New Zealand; 3 Discovery Services, Charles River, Freiburg, Germany; Università degli Studi della Campania, ITALY

## Abstract

Soft tissue is composed of cells surrounded by an extracellular matrix that is made up of a diverse array of intricately organized proteins. These distinct components work in concert to maintain homeostasis and respond to tissue damage. During tissue repair, extracellular matrix proteins and their degradation products are known to influence physiological processes such as angiogenesis and inflammation. In this study we developed a discovery platform using a decellularized extracellular matrix biomaterial to identify new chemotrophic factors derived from the extracellular matrix. An *in vitro* culture of RAW.264 macrophage cells with the biomaterial ovine forestomach matrix led to the identification of a novel ~12 kDa chemotactic factor, termed ‘MayDay’, derived from the N-terminal 31–188 sequence of decorin. The recombinant MayDay protein was shown to be a chemotactic agent for mesenchymal stromal cells *in vitro* and *in vivo*. We hypothesize that the macrophage-induced cleavage of decorin, via MMP-12, leads to the release of the chemotactic molecule MayDay, that in turn recruits cells to the site of damaged tissue.

## Introduction

Progenitor cells play an important role in the constructive remodeling of soft tissues following damage or disease. Recruitment of progenitor cells to the site of injury is facilitated by a range of signaling molecules, for example platelet-derived growth factor, stromal cell-derived factor 1 (SDF-1), and insulin-like growth factor [1–4]. Signaling molecules are expressed by several different cell types in response to tissue injury, and the extracellular matrix (ECM) sequesters and stores signaling molecules, and releases them when tissue is damaged [5–7]. A well characterized example of progenitor cell recruitment occurs through SDF-1 signaling, where SDF-1 is up-regulated at sites of tissue damage and recruits endogenous mesenchymal stem cells via the CXC chemokine receptor 4 [8]. This phenomenon has been reproduced *in vitro* using bone marrow-derived mesenchymal stromal cells (BM-MSCs) [4, 9–13] and adipose-derived mesenchymal stromal cells (AD-MSCs) [1, 11] and in an *in vivo* model of skeletal fracture repair [14].

Macrophages are one of the first responders to tissue damage and have a well-established role in constructive remodeling. Importantly, macrophages express matrix metalloproteases (MMPs) that degrade and remodel the ECM during tissue repair, liberating ECM-bound signaling molecules [15]. Macrophages can be activated via different mechanisms towards a classical (M1) or alternative (M2) phenotype, which support inflammation or remodeling respectively [16]. The profile of MMPs secreted by macrophages is dependent on their phenotype and expression changes over the course of soft tissue repair [17].

Decellularized extracellular matrix (dECM) biomaterials derived from various mammalian tissue sources have been developed for a range of soft tissue repair applications [18–20]. These biomaterials retain much of the biochemical composition and structure of tissue ECM and serve as a temporary scaffold of cell infiltration, proliferation and the regeneration of soft tissue. Like tissue ECM, dECMs undergo constructive remodeling over time and are completely absorbed into the regenerating soft tissue [21]. Ovine forestomach matrix (OFM) is a dECM derived from the rumen of sheep, specially the propria submucosa, and has been shown to contain a number of ECM proteins including 24 different collagens, proteoglycans, including perlecan and decorin (DCN), cytokines and growth factors [22, 23]. *In vivo* studies have demonstrated that OFM is anti-inflammatory [24], stimulates angiogenesis [25] and is remodeled over time [26]. Clinically, OFM has found a range of applications in soft tissue repair, including wound healing [27–33], reconstructive surgery [34], and abdominal wall repair [35]. Given that dECM biomaterials contain a milieu of growth factor binding proteins, cytokines and growth factors, it is not surprising these biomaterials recruit MSCs *in vivo* and *in vitro*. For example, a dehydrated human amnion/chorion membrane dECM was shown to increase proliferation and migration of both AD-MSCs and BM-MSCs *in vitro* [36]. Decellularized muscle tissue material has also shown to promote myogenic cell migration and satellite cell homing in a rat model of volumetric muscle loss [37].

Regenerative medicine has sought to harness the potential therapeutic benefit of MSCs for soft tissue repair. Traditional strategies have focused on the isolation, expansion and delivery of allogeneic or autologous stem cell populations to the site of tissue damage [38, 39]. However, an alternate approach is to recruit endogenous (allogeneic) MSCs via a chemotactic agent to the site of damage, thus eliminating the time, cost and potential complications that are associated with the isolation and culture of MSCs *ex vivo* [40]. With an aim to find novel MSC chemotactic agents we exploited the natural reservoir of chemotactic factors stored in tissue ECM and developed a discovery platform utilizing a dECM co-cultured with biologically relevant cells. Specifically, OFM and macrophages were used to isolate and identify a novel ECM-derived chemotactic agent that recruited MSCs *in vivo* and *in vitro*.

## Materials and methods

### General

Ovine forestomach matrix (OFM), terminally sterilized with ethylene oxide was supplied by Aroa Biosurgery Limited (Auckland, New Zealand). Cells were maintained in Dulbeccós modified Eaglés Medium (DMEM) (Gibco™, Waltham, MA, USA) supplemented with Fetal Bovine Serum (FBS) (Gibco) as required to final concentrations of 0.5% (DMEM0.5), 2% (DMEM2), 5% (DMEM5) or 15% (DMEM15) and 1% Penicillin-Streptomycin (10,000 U/mL) (Gibco). All cultures were maintained in a 5% CO_2_ atmosphere with 95% humidity at 37°C.

Murine animal manipulations were carried out in strict accordance with the recommendations in the Guide for the Care and Use of Laboratory Animals of the Society of Laboratory Animals (GV SOLAS) in an AAALAC accredited animal facility. All animal experiments were approved by the Committee on the Ethics of Animal Experiments of the regional council (Regierungspräsidium Freiburg, Abt. Landwirtschaft, Ländlicher Raum, Veterinär- und Lebensmittelwesen—Ref. 35, permit #:G-16/97). Ovine animal manipulations were conducted in accordance with applicable New Zealand animal welfare regulations and under approval of a local animal ethics committee (AgResearch AE Application 13941).

### Isolation of ovAD-MSC

Ovine adipose derived stomal cells (ovAD-MSC) were isolated from subcutaneous fat tissue from donor animals according to the method of Li *et al*. [41]. Briefly, adipose tissue was aseptically excised from the shoulder of adult female sheep. Tissue specimens were cut to ~1 x 1 cm and rinsed in Dulbeccós Phosphate Buffered Saline (DPBS) (Gibco) (3x, 20 mL) at rt°C for 10 mins. Tissues were minced and then digested with 0.1% collagenase/DPBS (10 mL) from *Clostridium histolyticum* (Sigma-Aldrich, St Louis, MI, USA) for 1 h at 37°C, with gentle shaking at 50 rpm. An equal volume of DMEM5 was added and incubated overnight on a 100 mm cell culture plate (Corning, NY, USA). Adherent cells were rinsed (DMEM2, 10 mL) and passaged in DMEM2 for 3 passages. Cells were maintained in DMEM2 (10 mL) with media changed every 3 days and trypsinized using TrypLE™ Express (1.5 mL) (Gibco) once a week.

### Differentiation of ovAD-MSC

Cells (ovAD-MSCs, passage 3) were split and seeded onto 24-well plates (Corning) in DMEM2 at a concentration of 100,000 cells/mL (0.5 mL) and incubated until monolayers were 80% confluent. Media was changed to osteogenic, chorondrogenic or adipogenic differentiating medias (1 mL) (StemPro™ Osteogenesis Differentiation Kit, Adipogenesis Differentiation Kit, Chondrogenesis Differentiation Kit, Life technologies, Carlsbad, US). Cells were maintained for two weeks in the respective media, with media changed every 3 days. Differentiated cell monolayers were rinsed in DPBS (1 mL) then fixed in 10% neutral buffered formalin (1 mL) (Sigma-Aldrich) for 10 mins at rt°C. Monolayers of adipocytes were stained with Oil Red O (0.5% w/v isopropanol, 1 mL, rt°C, 10 min) (Sigma-Aldrich) and counter stained with 0.1% haemotoxylin (1 mL, rt°C, 10 min) (Sigma-Aldrich). Osteocytes were stained with 2% Alizarin Red S (1 mL, rt°C, 10 min) (Sigma-Aldrich). Chondrocytes were stained with Toluidine Blue (1 mL, 0.1% w/v, rt°C, 10 min) (Sigma-Aldrich). Stained monolayers were rinsed with ROH_2_O (3x, 1 mL) then imaged using an Olympus inverted phase contrast and microscope (IX51, Olympus, Tokyo, Japan).

### Isolation and differentiation of muBM-MSC

Murine bone marrow derived stromal cells (muBM-MSCs) were isolated from femur and tibia of freshly euthanized mice according to the method of Soleimani et al.[42]. Briefly, Balb/c mice (n = 10) (Charles River, Sulzfeld, Germany) were euthanized via cervical dislocation and the femur and tibia surgically dissected. The ends of tibia and femur were cut to open the bone marrow cavity. The cavity was flushed using a 27-gauge needle attached to a 10 mL syringe containing DMEM15 (5 mL) with 0.1% Gentamycin (Life Technologies, Carlsbad, US) and 5% Amphotericin B (Sigma-Aldrich) and aspirate collected into a 15 mL tube on ice. Bone marrow aspirate was incubated in DMEM15 (2 mL) with 0.1% Gentamycin (Life Technologies,) and 5% Amphotericin B (Sigma-Aldrich) for 3 h on 24-well Primaria™ tissue culture plates (Corning). Cultures were then rinsed with PBS (3x, 1 mL per well) to remove non adherent cells. Adherent muBM-MSC’s were maintained in DMEM15 (1 mL per well) then split using TrypLE™ Express (0.5 mL) (Gibco) to Primaria™ tissue culture plates and expanded for 2 to 4 passages, with media changed every 3 days, and splitting once per week until a stable cell line was established.

### Differentiation of muBM-MSC

muBM-MSCs were seeded onto Primaria™ 6-well plates and cultured in differentiating media (2 mL) (StemPro™ Osteogenesis Differentiation Kit, Adipogenesis Differentiation Kit, Chondrogenesis Differentiation Kit, Life technologies). For osteocyte and adipocyte differentiation cells were seeded at 80,000 per well, for chondrocyte differentiation cells were plated as 5 μL droplets of cell solution at a concentration of 160,000,000 cells /mL. Cells were cultured for 2–4 weeks before fixing with 4% paraformaldehyde (2 mL) (Sigma-Aldrich). Cultures were stained with either; 1% Lipidtoxgreen (2 mL) (Thermo Fisher Scientific, Waltham, Massachusetts), 2% Alizarin Red (2 mL) (Abcam, Milton, UK), 1% Alcian Blue (2 mL) (Sigma-Aldrich). Cultures rinsed with PBS (3x, 2 mL) then imaged at 100x using an Axiovert 35 inverted phase contrast and fluorescence microscope (Zeiss, Jena, Germany).

### Characterization of muBM-MSC by flow cytometry

Characterization of muBM-MSC was carried by flow cytometry using a mouse mesenchymal stem cell marker antibody panel (R&D systems, Minneapolis, US). Cells at passage 3, were split to a 96-well plate (50,000 cells/well) (Corning), and rinsed in DPBS (3x, 20 μL). Epitopes were blocked with purified rat anti-mouse CD16/CD32 (Mouse BD Fc Block™, BD Biosciences, Franklin Lakes, NJ, USA) (10 μL) diluted to 50 μg/mL with FACS Buffer (2% FBS in PBS) and the plate incubated for 5 mins at rt°C. Cells were stained with the antibodies; rat anti-mouse Sca-1 IgG2A monoclonal antibody, rat anti-mouse CD29 IgG2A monoclonal antibody, sheep anti-mouse CD44 IgG2B monoclonal antibody, rat anti-mouse CD73 IgG2A monoclonal antibody, rat anti-mouse CD105 IgG2A monoclonal antibody, rat anti-mouse CD106 IgG2A monoclonal antibody. Negative MSC markers included: rat anti-mouse CD11b IgG2B and rat anti-mouse CD45 IgG2B (R&D systems, Minneapolis, US). The primary rat antibodies were detected with secondary goat anti-rat IgG (Jackson Immuno Research, West Grove, PA, USA) and the primary sheep antibodies with a donkey anti-sheep IgG (R&D systems). All antibodies were diluted to give a final concentration of 1 μg/well (100 μL), prepared as a solution in Aqua Zombie™ (Biolegend, San Diego, CA, USA) live dead stain (1:100 diluted in PBS). Plates were incubated for 30 mins at 4°C in the dark, then washed with FACS buffer (3x, 200 μL). Plates were centrifuged at 400 rpm, 5 mins, and the supernatant removed. Stained cells were resuspended FACS buffer (200 μL). Cell suspensions were analyzed using an ATTUNE NXT Acoustic Focusing Cytometer (Thermo Fisher Scientific).

### Macrophage OFM co-culture

OFM was cut to ~4 x 4 cm samples and pre-conditioned in DMEM (2 mL) at 37°C, for 16 h, in 100 mm culture plates (Corning). RAW 264.7 (ATCC, TIB-71) [43] macrophages (M0ϕ) in DMEM (1 mL at 100,000 cells/mL) were seeded onto OFM (~100,000 cells) and incubated (30 min, 37°C) to allow cell attachment. Additional DMEM was added to a final volume of 5 mL per well. Samples were incubated for 24 h at 37°C. As controls, the above procedure was carried out without OFM (Mϕ) and without macrophages (OFM). Media was aspirated, collected and phenylmethanesulfonyl fluoride (PMSF) (Sigma-Aldrich) added at a final concentration of 10 μM. Samples of conditioned media from the respective samples (Mϕ, OFM, OFM+Mϕ) were sterile filtered (0.22 μm) and stored at -20°C prior to use.

### Transwell migration assay

Transwell migration assays were conducted according to the method of Boyden *et al*. [44] using a 24-well transwell system (6.5 mm Transwell®, Corning). Conditioned media samples (OFM, Mϕ and OFM+Mϕ) were diluted 1:1 in DMEM and supplemented with FBS to a final concentration of 0.5% (DMEM0.5). DMEM0.5 and recombinant human FGF2 (Sigma-Aldrich) (50 ng/mL in DMEM0.5) were used as negative and positive controls, respectively. For each well, 400 μL of each test sample were added to the lower chamber in triplicate. ovAD-MSC (passage 6) were trypsinized using TrypLE™ Express (1.5 mL) (Gibco) counted and resuspended in DMEM0.5 to 100,000 cells/mL. Cell suspensions (100 μL) were plated to the insert (upper chamber). Cultures were incubated for 6 h, then transwell membranes removed from plates and rinsed with DPBS (500 μL). Non-migrated cells were removed from the inserts using a cotton tip and inserts were fixed with ice cold methanol (0.5 mL) (Sigma) diluted to 80% v/v in ROH_2_O for 10 mins. Fixed inserts were transferred to a new plate containing 0.5 mL of 0.5% (w/v) crystal violet (Sigma-Aldrich) staining solution in 20% methanol/ ROH_2_O (v/v) for 30 mins. Inserts were rinsed with ROH_2_O (3x, 100 mL), then dried. Cells were imaged by inverted microscope (IX51, Olympus, Tokyo, Japan) at 400x magnification, taking five representative images per insert across the entire insert. The number of migrated cells was counted manually using ImageJ (NIH, Bethseda, USA), and multiplied by the area of the membrane (0.33 cm^2^) to determine the total number of migrated cells per insert. The number of migrated cells was expressed relative to the number of cells that migrated in the media only controls. Results were expressed as Normalized Cell Migration, relative to the media only controls, and represent the average from three independent experiments. Statistical analysis (t-test) was conducted using GraphPad Prism (ver 8.4.1) (Graphpad Software Inc, CA, USA); ‘*’, p<0.05; ‘**’, p<0.01; ‘***’, p<0.001; ‘****’, p<0.0001.

### FITC staining OFM

A 10 mg/mL stock solution of fluorescein isothiocyanate (FITC) (Sigma-Aldrich) was prepared in dimethyl sulfoxide (DMSO) (Sigma-Aldrich) and diluted in sodium bicarbonate (0.1 M NaHCO_3_, pH 9.3) prior to use. OFM (approx. 100 mg) was incubated for 1 h at 4°C with 10 mL FITC at a final concentration of 10 μg/mL (OFM_FITC10_). The reaction was quenched via the addition of 0.1 M Tris-HCl (pH 9.6). OFM was rinsed in DPBS (3x, 30 mins, 100 rpm, rt°C). The resultant FITC labelled OFM (OFM_FITC10_) was stored at 4°C prior to use.

FITC labelled OFM was used to generate conditioned media as described above. Briefly, OFM or OFM_FITC10_ were cut to ~1 x 1 cm samples and pre-conditioned in DMEM at 37°C, for 16 h, in 24-well plates. RAW 264.7 murine macrophages (Mϕ) in DMEM (500 μL, 50,000 cells/mL) were seeded onto the OFM and incubated (30 mins, 37°C) to allow cell attachment. Additional DMEM was added to a final volume of 1 mL per well. Samples were incubated for 48 h at 37°C (5% CO_2_). Samples of conditioned media from the respective samples (Mϕ, OFM, OFM+Mϕ) or OFM_FITC10_+ Mϕ) were sterile filtered (0.22 μm) and stored at -20°C prior to use

### Tris-glycine SDS-PAGE separation of conditioned media

Samples of conditioned media (30 μL) were diluted 3:1 with 4x Laemlie buffer (100 mM Tris, pH 6.8, 8% w/v SDS, 40% v/v glycerol, 20% w/v β-mercaptoethanol, 0.2% w/v bromophenol blue). Samples were boiled in a water bath at 100°C for 10 mins. Tris-glycine gels (4% acrylamide stacking gel and a 20% acrylamide resolving gel) were made with a BioRad gel system and run with a glycine running buffer (25 mM Tris, 192 mM glycine, 0.1% w/v SDS, pH 8.3) (Sigma-Aldrich). A total volume of 15 μL of each sample was loaded per well and 8 μL of protein standard ladder (Precision Plus Protein™ Dual Color Standards, BioRad, Hercules, CA, United States). Tris-glycine gels were run for 1 h at 100 V. Fluorescent protein bands were visualized on a Fluoroskan Ascent FL (Thermo Fisher Scientific) before staining with Coomassie brilliant blue (0.1% w/v Coomassie Brilliant Blue R-250, 50% v/v methanol, 10% glacial acetic acid) for 3 h at rt°C with gentle shaking. Coomassie stained gel were imaged using a Typhoon FLA 9500 (GE HealthCare, Chicago, IL, USA). Raw gel images are included in S2 File.

### Identification of proteins by electrospray ionization mass spectrometry

#### Sample preparation

OFM (4 x 4 cm) was labelled with 0 and 10 μg/mL FITC in 0.1 M sodium bicarbonate as described above. The resulting labelled and unlabeled materials (OFM and OFM_FITC10_) were conditioned for 16 h in DMEM (5 mL). OFM and macrophage co-cultures were conducted as described above with the modification of ~50,000 RAW265.7 macrophage cells per 4 x 4 cm OFM sample in a final volume of 0.5 mL DMEM. Cultures were incubated for 24 h and conditioned media from these samples (OFM+Mϕ, OFM_FITC10_+Mϕ and Mϕ alone) collected. Samples were sterile filtered (0.22 μm) and treated with PMSF, as described above. Samples were desalted with PBS (5 mL) and concentrated by ultrafiltration using Amicon Ultra-15 centrifuge filters (Ultracel-PL membrane, 3 kDa, Merk/Millipore, Burlington, Massachusetts, United States) and stored at -20°C before use.

Protein quantification was carried out using a Bicinchoninic Acid (BCA) kit for protein determination (Sigma-Aldrich) according to the manufacturer’s instructions.

#### In solution trypsin digestion

A sample of OFM+Mϕ was resuspended in PBS to a final protein concentration of 0.1 mg/mL. Samples (20 μg) were reduced with 10 mM 1,4-dithiothreitol (DTT) (Sigma-Aldrich) (20 μL, 60 mins, 60°C), then alkylated with 20 mM iodoacetamide (Sigma-Aldrich) (20 μL, 30 mins, rt°C in the dark). Sample (60 μL) were digested overnight (37°C) with 0.1 μg trypsin (Sigma-Aldrich). The sample was dried, then reconstituted in loading buffer (0.1 M sodium bicarbonate) prior to ESI MS/MS analysis.

#### Size exclusion chromatography

Lyophilized samples OFM+Mϕ conditioned media, were resuspended in PBS to a final protein concentration of 0.1 mg/mL. Samples (100 μL, ~ 100 μg) were subjected to size-exclusion chromatography (SEC) (GE Superdex 75 10/300 GL, GE Healthcare, MA, USA) and fractionated into a 96 well plate, using a mobile phase of 50 mM sodium phosphate (pH 7), 150 mM NaCl, and a flow rate of 0.35 mL/min. Elutant was monitored at 214, 220 and 280 nm. Fractions were pooled based on the known retention times and molecular weights of the following standards: aldolase, conalbumin, carbonic anhydrase, RNaseA, and aprotinin. Pooled samples (~1 mL) were reduced with 10 mM DTT at 60°C for 1 h, then alkylated with 25 mM iodoacetamide (30 mins, rt°C). Trypsin (500 ng) was added and samples digested overnight at 37°C. Samples were desalted using a OMIX C_18_ 100 μL tip (Agilent/Varian, A57003100K, Santa Clara, CA, USA), prior to eluting in 100 μL acetonitrile (ACN)/formic acid. The samples were dried, then reconstituted in loading buffer prior to ESI MS/MS analysis.

#### Tris-Tricine SDS-PAGE and in-gel protein digestion

Samples (OFM+Mϕ_,_ OFM_FITC10_+Mϕ and Mϕ only) were prepared as described above, then diluted 3:1 with 4x Laemlie buffer and denatured, as described above. A total volume of 15 μL of each sample was loaded per well and 8 μL of protein standard ladder (Precision Plus Protein™ Dual Color Standards, BioRad). Tris-Tricine gels (4% acrylamide stacking gel and a 16% acrylamide resolving gel) were run using a cathode buffer (100 mM Tris, 100 mM tricine, 0.1% w/v SDS, pH 8.25) and an anode buffer (100 mM Tris, pH 8.9). Gels were run for 2 h at 60 V on ice (~4°C). Tris-Tricine gels were fluorescently visualized on a Fluoroskan Ascent FL (Thermo Fisher Scientific) before staining with Coomassie brilliant blue (0.1% Coomassie Brilliant Blue R-250, 50% v/v methanol and 10% glacial acetic acid) for 3 h at rt°C with gentle shaking. Coomassie stained gel were imaged using a Typhoon FLA 9500 scanner (GE Healthcare, Chicago, IL, USA).

The area of interest, corresponding to the band with MW 12 kDa was excised from the gel from lanes containing both OFM+Mϕ, OFM_FITC10_+Mϕ. The sample was reduced with DTT (10 mM, 20 μL) for 1 h at 60°C, then alkylated with iodoacetamide (20 mM, 20 μL) for 30 mins in dark at room temperature. Proteins were digested with 100 ng of trypsin overnight at rt°C. The samples were dried, then reconstituted in loading buffer prior to ESI MS/MS analysis

#### ESI MS/MS analysis

Injections were made to an Eksigent Ultra nanoLC system (Eksigent, Livermore, CA USA), coupled to a Triple TOF 5600 (AB Sciex, Redwood City, CA, USA). Digested samples (reconstituted to 10, 20 or 40 μL volumes) was injected onto a peptide trap (peptide Captrap, Michrom Bioresources, Auburn, CA, USA) and desalted with 0.1% aqueous formic acid/2% acetonitrile (ACN), at 10 μL/min for 5 mins. The peptide trap was then switched into line to an analytical column (Halo C18, 160Å, 2.7 μm, 75 μm x 10 cm, Advances Materials Inc., Wilmington, DE, USA). Peptides from trypsin digested samples and in-gel digested samples, were eluted from the column using a solvent gradient; 95% (aqueous 0.1% formic acid)/5% (99.9% ACN/0.1% formic acid) to 60% (aqueous 0.1% formic acid)/40% (99.9% ACN/0.1% formic acid), at a flow rate of 550 nL/min over a 42 min period. Peptides from SEC were eluted from the column using a solvent gradient; H_2_O:ACN (95:5; + 0.1% formic acid) to H_2_O:ACN (5:95; + 0.1% formic acid) with constant flow (500 nL/min) over an 80 min period.

The eluent was subject to positive ion nanoflow electrospray analysis in an information dependent acquisition (IDA) mode. In IDA mode a TOFMS survey scan was acquired (m/z 350–1500, 0.25 second), with the ten most intense multiply charged ions (counts >150) in the survey scan sequentially subjected to MS/MS analysis. MS/MS spectra were accumulated for 200 milliseconds in the mass range m/z 100–1500 with the total cycle time 2.3 seconds. The raw data files (.wiff) were converted to mascot generic files (.mgf) using AB SCIEX CommandDriver software (AB SCIEX, Redwood City, CA, USA). Data files were submitted to Mascot (Matrix Science, UK) and searched against Swissprot database (*Ovis aries* [sp_sheep_140625].

### Computational digestion of DCN

The MEROPS database (http://merops.sanger.ac.uk/) was used to search for known proteolytic cleavage sites of human DCN, (amino acid residues 1–359) using the sequence obtained from NCBI (accession number: P07585).

### *In vitro* digestion of DCN by MMP-12

Stock solutions of MMP-12 catalytic domain (Sino Biologicals, Beijing China), and DCN (Sino Biologicals) were prepared at 0.25 mg/mL in ROH_2_O and stored at -20°C prior to use. Samples of DCN (10 μL) were digested with 0, 0.1, 5, or 10 μL the MMP-12 solution, to give protein:enzyme ratios of 1:0, 100:1, 2:1 or 1:1. Samples were made up to a final volume of 40 μL with MMP-12 buffer (50 mM Tris, NaCl 100 mM, 0.05% w/v Brij35, pH 8.0). The samples were incubated at 37°C for 16 h, with gentle shaking.

Digested Samples (30 μL) were diluted 3:1 with 4x Laemlie buffer and denatured, as described above. A total volume of 30 μL was loaded onto precast Bis-Tris gels (4–12% Bolt NuPAGE, Invitrogen, Carlsbad, CA, USA). Bis-Tris gels were run with a protein standard solution (5 μL) (SeeBlue protein standard, Invitrogen) using an Invitrogen Mini Gel system (Invitrogen™) in a BOLT running buffer (Bolt™ MES SDS Running Buffer, Invitrogen™) for 90 mins at 100 V. Gels were rinsed (3x, ROH_2_O, 10 mL) and then stained with Coomassie as described above. Raw gel images are included in S2 File.

For transwell migration assays, samples were prepared as follows: DCN (20 μL at 0.25 mg/mL) and MMP-12 (10 μL at 0.25 mg/mL) were made up in to 40 μL with digestion buffer giving a protein:enzyme ratio of 2:1, as described above. The samples were incubated overnight at 37°C for 16 h. As controls, DCN (20 μL at 0.25 mg/mL) and MMP-12 (10 μL at 0.25 mg/mL) were made up to 40 μL with digestion buffer and a sample of digestion buffer alone (‘control’) were incubated for the same length of time. After incubation, each solution was combined with 0.5 mL DMEM0.5 to quench enzymatic digestion. Transwell migration assay using ovAD-MSC cells was conducted as described above. Results were expressed as Normalized Cell Migration, relative to the media only samples, and represent the average from three independent experiments. Statistical analysis (t-test) was conducted using GraphPad Prism (ver 8.4.1); ‘*’, p<0.05; ‘**’, p<0.01; ‘***’, p<0.001; ‘****’, p<0.0001.

### Bioactivity of recombinant His-MayDay(31–170)

Recombinant HIS tagged MayDay(31–170) (rec-HIS*ov*MayDay(31–170)) was expressed and purified by Biomatik (Ontario, Canada), according to standard procedures. Briefly, the ovine DCN sequence 31–170 with a 6xHis-tag fused to its N-terminus was cloned into a pET30a cloning vector. The expression plasmid was transformed into *Eschericha coli* BL21 and grown at 37°C in Luria Broth (LB) media supplemented with 50 μg/mL Kanamycin (Sigma-Aldrich) until OD600 nm of 0.6 was reached, then Isopropyl β- d-1-thiogalactopyranoside (IPTG) (0.2 mM) (Sigma-Aldrich) was added to the media, and the culture was further incubated for 16 h at 15°C. The cells were harvested by centrifugation, pellet resuspended and sonicated in lysis buffer (50 mM Tris, pH 8.5, 300 mM NaCl, 20 mM imidazole) (Sigma-Aldrich). The cell debris was pelleted by centrifugation and supernatant loaded onto a Ni-IDA affinity column pre-equilibrated with lysis buffer, centrifuged and supernatant collected. Fractions were analyzed by SDS-PAGE. Fractions were pooled and dialyzed against the final buffer (50 mM Tris, pH 8.5,150 mM NaCl). Recombinant protein sequences are provided in S4 Fig.

Recombinant His-tagged MayDay (31–171) (rec-HIS*ov*MayDay(31–170)) was tested in a transwell migration assay using ovAD-MSC, as described above. rec-HIS*ov*MayDay(31–170) was a prepared in PBS (0.1 mg/mL), then diluted to a final concentration of 0.05, 0.50 and 5.00 ng/mL in DMEM0.5. Human recombinant SDF-1 (Sigma) was prepared in PBS (0.1 mg/mL) and diluted to a final concentration of 50 ng/mL in DMEM0.5. Results were expressed as Normalized Cell Migration, relative to the media only samples, and represent the average from three independent experiments. Statistical analysis (t-test) was conducted using GraphPad Prism (ver 8.4.1); ‘*’, p<0.05; ‘**’, p<0.01; ‘***’, p<0.001; ‘****’, p<0.0001.

### *In vivo* model of mesenchymal stromal cell recruitment

#### Recombinant MayDay(31–170) expression

Tag free protein (rec-*ov*MayDay(31–170)) was expressed as described above using a pSUMO vector in BL21 *E*. *coli*. After expansion, expression and lysis, supernatant was loaded onto a Q SepharoseTM fast flow pre-equilibrated with lysis buffer, centrifuge e and the supernatant, analyze fraction by SDS-PAGE. Purity (>85%) was confirmed by SDS-PAGE. Lyophilized proteins were stored at -20°C. Recombinant protein sequences are provided in S4 Fig.

#### Fluorescent labeling of muBM-MSC

muBM-MSC were labeled immediately prior to injection into Balb/c mice using the Cellvue NIR815 fluorescent cell labeling kit (Licor, Lincoln, US). A plate of muBM-MSC at 80% confluency where resuspended in DMEM (5 mL), centrifuged and resuspended in Diluent C (Licor, Lincoln, US) to give a final concentration of 2x10^7^ cells/mL. Cells were labeled with a near infrared dye (NIR815), as per manufacturer’s instructions. Briefly, CellVue dye stock solution (2 μL, 4x10^-6^ M) was added to Diluent C (1 mL). The dye was then added to muBM-MSC in Diluent C (1 mL) and incubated at 37°C for 5 mins. The reaction was quenched with FBS (2 mL). Cells were pelleted by centrifugation at 400 rpm for 10 mins, then rinsed with PBS (3x, 10 mL). After final wash cells were resuspended in DMEM15 (5 mL) and held at 37°C prior to use.

#### *In vivo* MSC chemotaxis

Test articles, recombinant MayDay (rec-*ov*MayDay31-170), and human recombinant SDF-1 (Sigma) were prepared in 0.9% sterile saline (Braun, Melsungen, Germany). Five treatment groups were used; rec-*ov*MayDay(31–170) [1 μg/animal, (~0.05 μg/kg); 10 μg/animal (~ 0.5 mg/kg) and 25 μg/animal (~1.25 mg/kg)]; SDF-1 10 μg/animal, (~0.5 mg/kg) and 0.9% sterile saline control. Balb/c mice were anesthetized using isoflurane and placed in a ventral recumbency with anesthetic gas administered via nose cone. Injection sites (hind limb and tail) were prepared with chlorhexidine wipes and test articles administered to the Balb/c mice (n = 3 per test article) via a 30 μL intramuscular injection to the right hind-limb muscle (‘treated’).

After 5–10 mins NIR815 labelled muBM-MSC (~5x10^6^ cells) were injected (5 mL/kg) into the tail vein of each animal. Animals were imaged using an optical imaging system (Pearl Trilogy, Licor, Lincoln, US) at pre-determined timepoints; 0, 3, 6, 12 and 24 h after administration of the labelled muBM-MSC. After 24 h animals were euthanized by cervical dislocation. Hindlimb muscle tissue from the ‘treated’ sites were dissected, as well as a matched ‘normal’ tissue from the left hind limb of each animal. Additionally, major organs (brain, spleen, liver, gut, kidney and lung) were harvested from all animals.

Explanted ‘treated’ and ‘normal’ muscle tissue was imaged using a Pearl Trilogy Imaging system on the 800 nm channel (ex: 786 nm, em: 814 nm) and the fluorescence signal (pixels) determined for each, using Image studio software (ver 5.2, Licor). For each sample, a background fluorescence signal (pixels) was also measured, based on an equivalent area surrounding the tissue sample. Sample fluorescence was determined based on the signal of the test sample (‘treated’ and ‘normal’), minus the corresponding background fluorescence.

## Results

### Bioactivity of conditioned media

**Fig 1:**
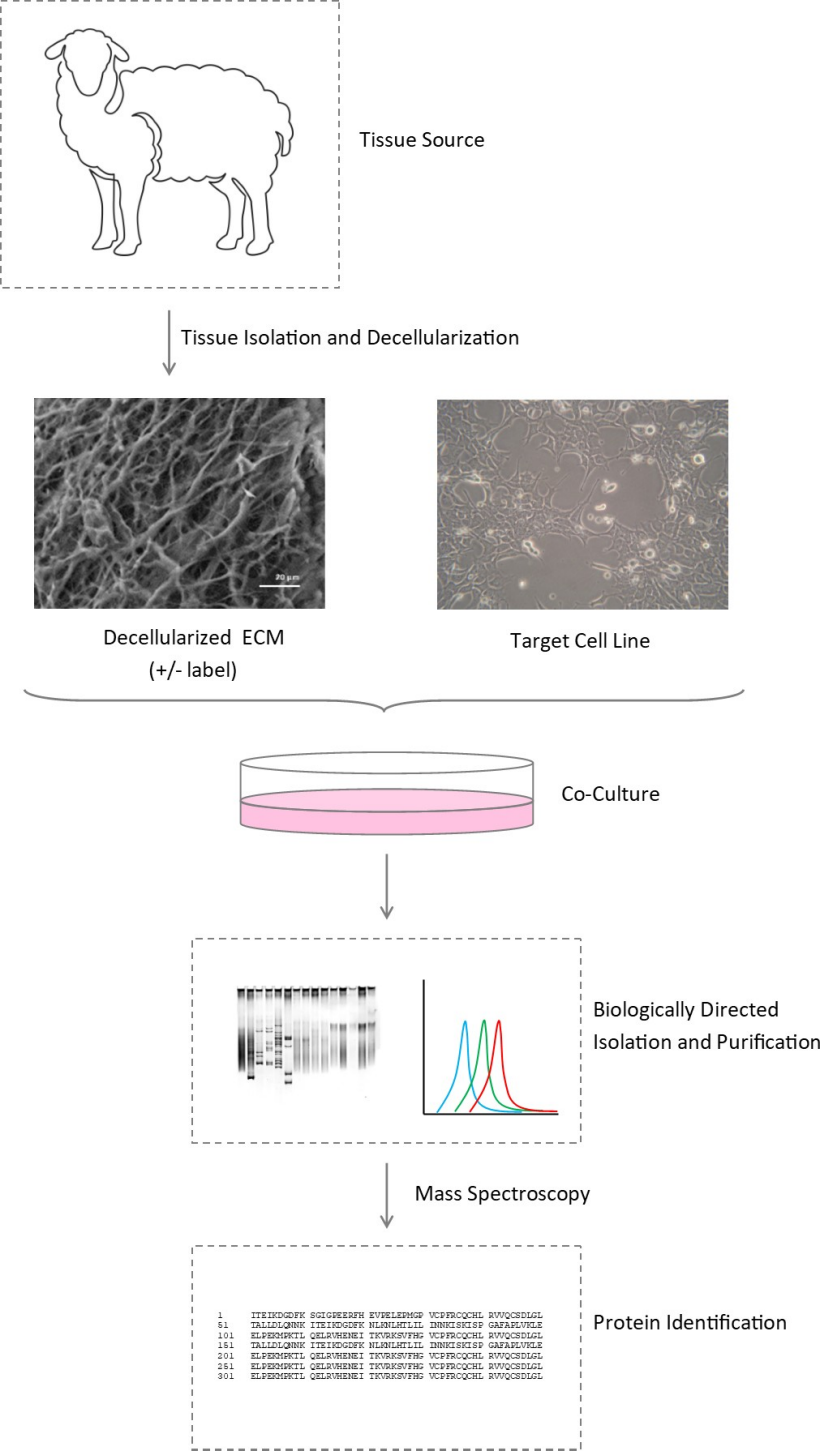
Schematic representation of the experimental design. A decellularized ECM (e.g. OFM), is produced from the raw tissue starting material of interest (e.g. ovine forestomach, nerve, lung), and incubated with the target cell line (e.g. macrophages, progenitor cells, neurons). Proteins of interest, generated from the co-culture, and originating from either the ECM or cells, can be purified and screened for bioactivity.

OFM, prepared from ovine forestomach tissue and terminal sterilized, was cultured with murine macrophages to generate conditioned media (OFM+Mϕ), according to the schematic representation of the discovery platform included in Fig 1.

**Fig 2:**
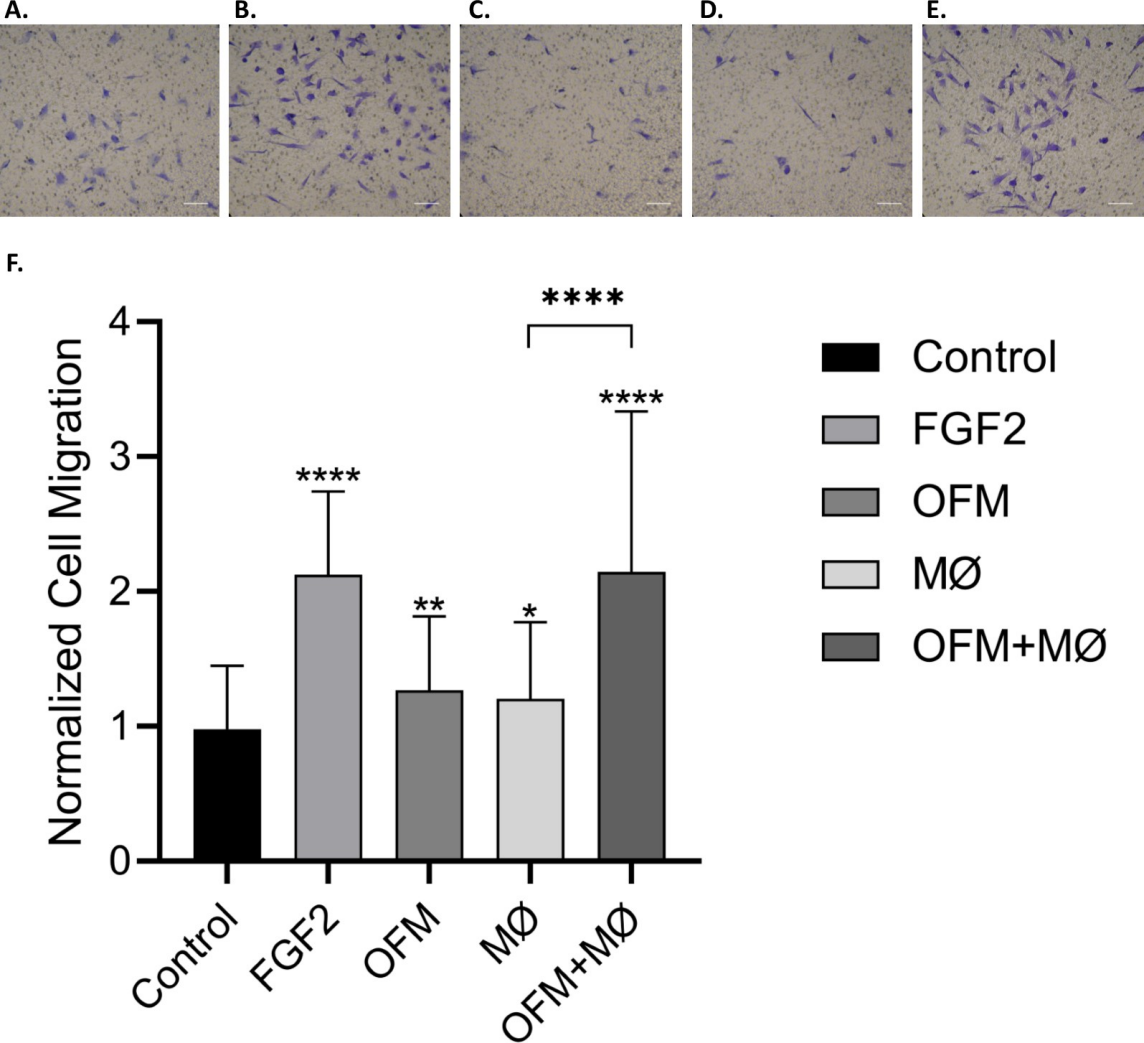
Bioactivity of conditioned media in a MSC migration assay. Conditioned media was generated from culture media containing OFM (OFM), RAW murine macrophage cells (Mϕ), and OFM co-cultured with macrophages (OFM+Mϕ). A transwell migration assay was conducted using ovAD-MSCs, with media alone (‘Control’) and FGF2 (50 ng/mL), included as the positive and negative controls respectively. Migrated ovAD-MSCs were imaged after 6 h. Representative photomicrographs of test groups are included in panels A through E (A. Media control; B. FGF2 (50 ng/mL); C. OFM; D. Mϕ; E. OFM+Mϕ). Cell migration was quantified and results are expressed as the average cell migration normalized to the media control (‘Normalized Cell Migration’) (F). Error bars represent standard deviation from three independent experiments. Statistical significance was determined via t-test, where; ‘*’p ≤ 0.05 ‘**’p ≤ 0.01 ‘***’p ≤ 0.001; ‘****’p ≤ 0.000.

A transwell migration assay, using ovAD-MSCs, was used to quantify the chemotactic activity of conditioned media. Isolated ovAD-MSCs were differentiated to demonstrate multipotency towards osteocytes, adipocytes and chondrocytes (S1 Fig). OFM+Mϕ conditioned media gave a significant increase in ovAD-MSC migration compared with the media only control (2.14±1.19 and 0.98±0.47, respectively, Fig 2). Conditioned media derived from macrophages alone (Mϕ, 1.21±0.57, Fig 2), or OFM alone (OFM, 1.27±0.55, Fig 2) increased cell migration relative to the media only control, but the combination of OFM and Mϕ (OFM+Mϕ) gave the greatest relative cell migration.

### Isolation and identification of the bioactive protein

**Fig 3:**
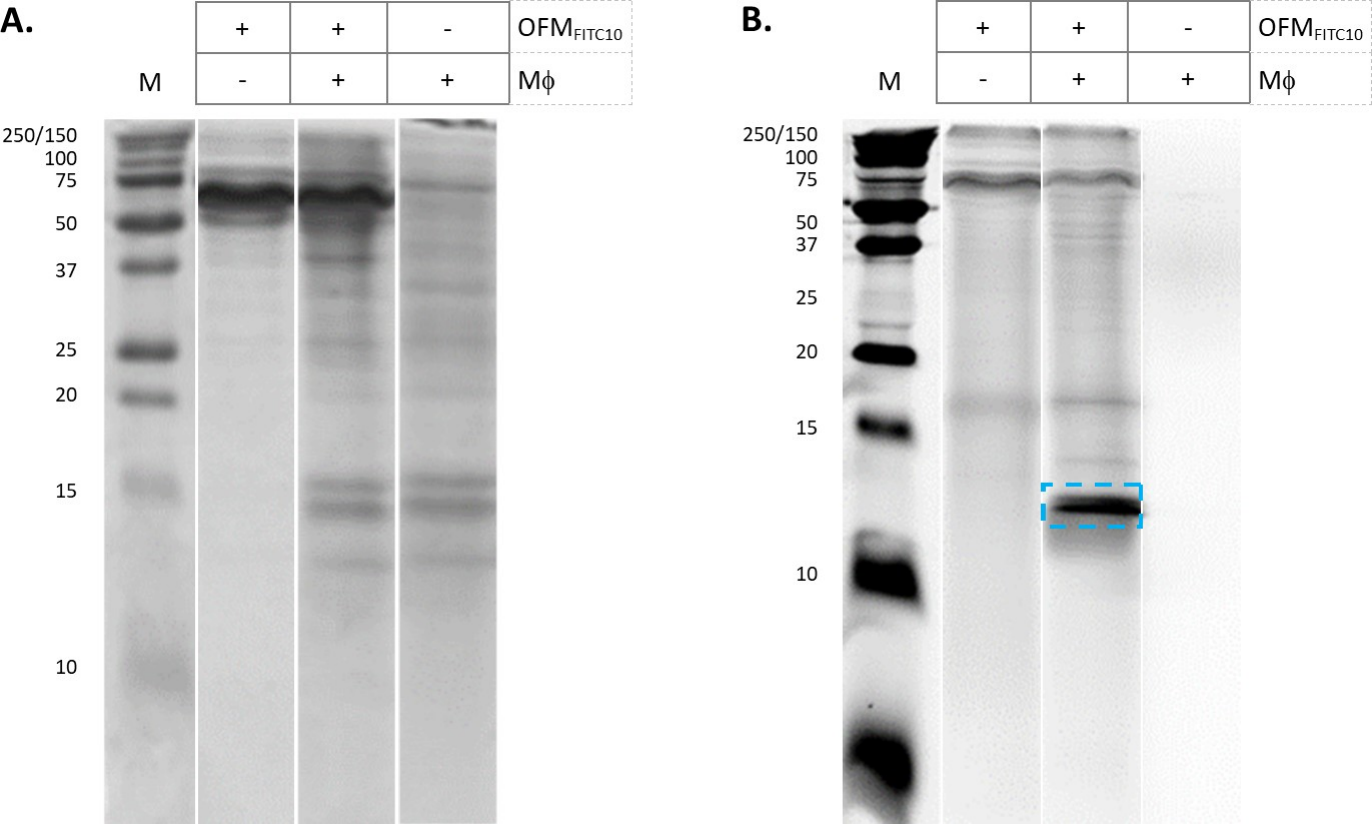
Tris-glycine SDS-PAGE of conditioned media. Conditioned media from cultures of OFM_FTIC10_, OFM_FITC10_+Mϕ, and Mϕ alone were separated by Tris-glycine SDS-PAGE electrophoresis. Tris-glycine gels were stained with either Coomassie (A) or imaged via a fluorescence scanner (B). The ~12 kDa protein band of interest highlighted in panel B (blue). Unedited gel images are provided in Supporting Information (S2. File).

FITC-labelled OFM (OFM_FITC10_) was prepared and cultured with macrophages to distinguish OFM-derived and macrophage derived proteins. Samples of conditioned media generated from OFM (OFM_FITC10_), macrophages (Mϕ) and a co-culture of OFM and macrophages (OFM_FITC10_+Mϕ) were separated on a Tris-glycine gel and the resultant protein bands imaged using a fluorescent scanner (Fig 3B). The sample originating from macrophages did not contain any fluorescently labelled protein bands (Fig 3B, lane 4), while the FITC labelled OFM sample contained high MW fluorescent protein bands (~75–250 kDa, Fig 3B, lane 2). The sample originating from a co-culture of FITC labelled OFM and macrophages (OFM_FITC10_+Mϕ) contained high MW fluorescent protein bands, as well as a fluorescently labelled protein band at ~12 kDa (blue dotted box, Fig 3B lane 3).

**Fig 4:**
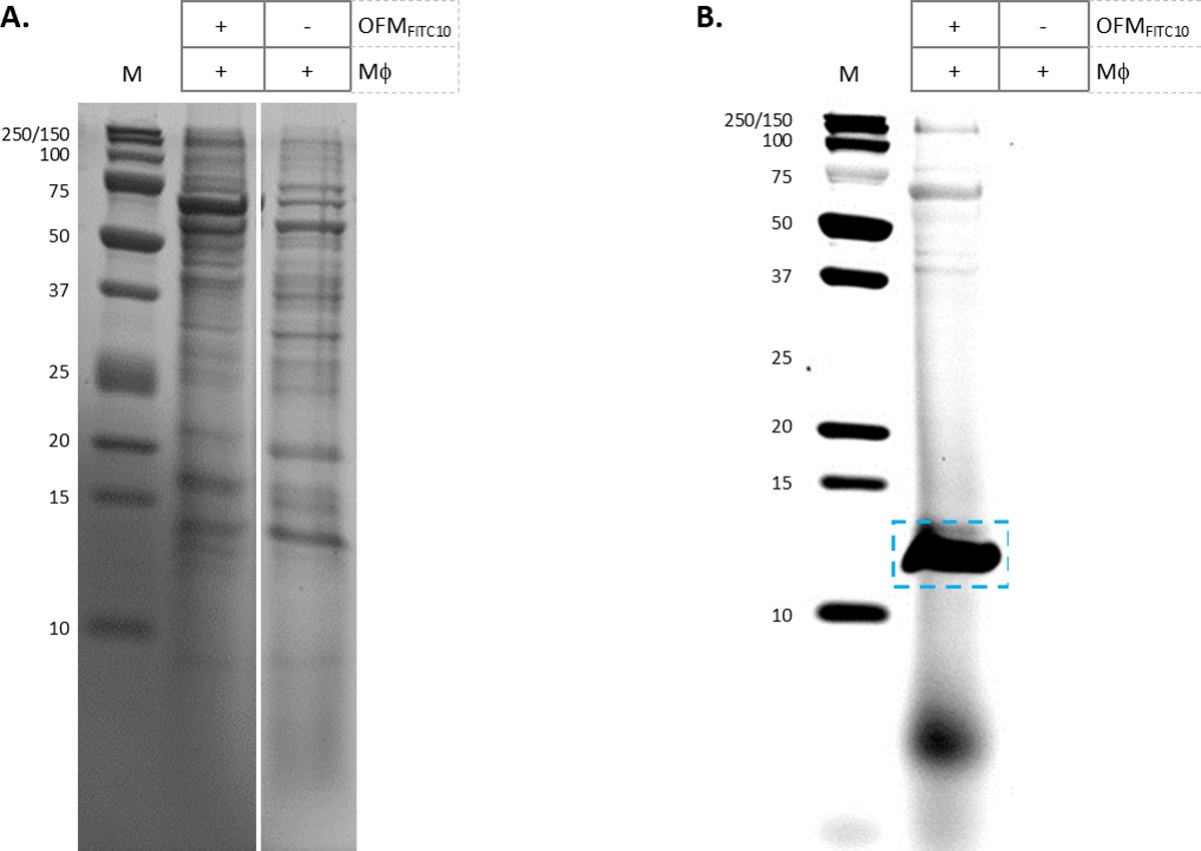
Tris-Tricine SDS-PAGE of conditioned media. Conditioned media from cultures of Mϕ and Mϕ+OFM_FTIC10_, were separated by Tris-Tricine SDS-PAGE electrophoresis. Tris-Tricine gels were stained with either Coomassie (A) or imaged via a fluorescence scanner (B). The ~12 kDa protein band of interest was excised for ESI/MS, (highlighted in panel B). Unedited gel images are provided in Supporting Information (S2 File).

**Fig 5:**
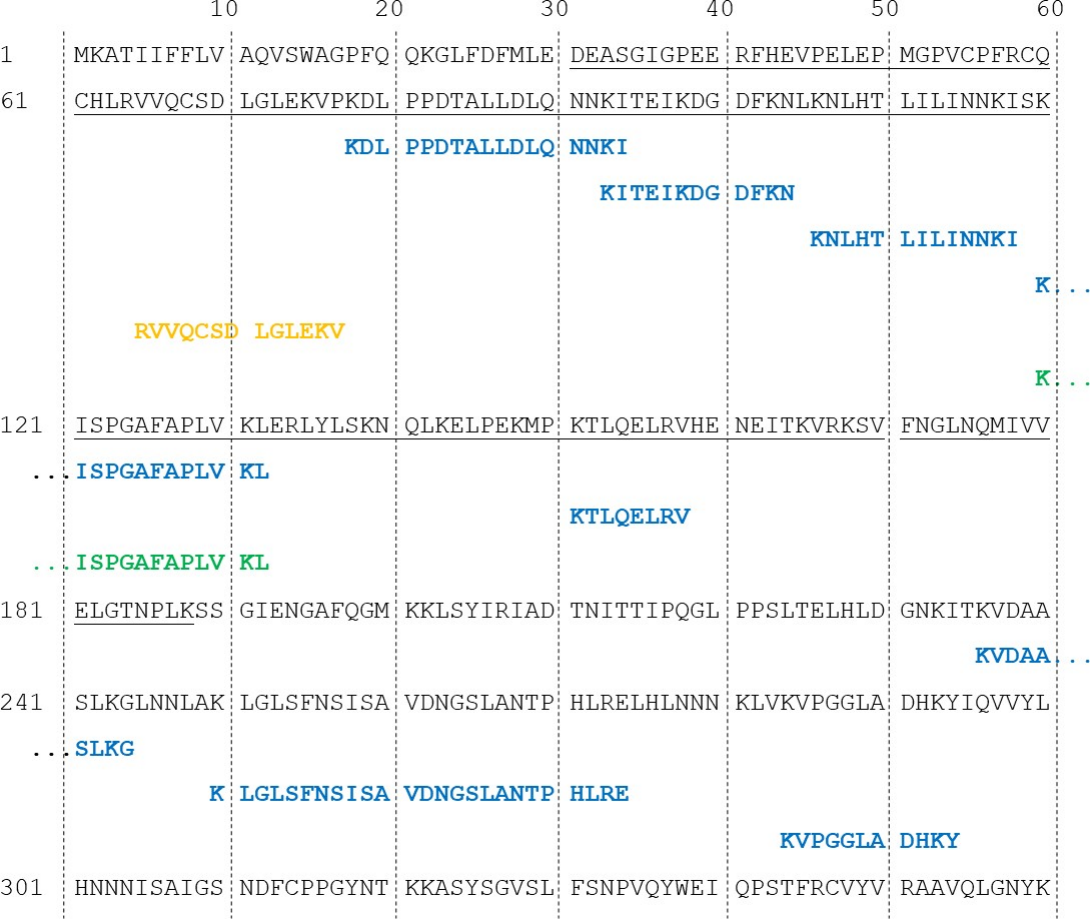
ESI MS/MS identification of DCN peptide fragments. Ovine DCN (1–360; accession number: Q9TTE2) (grey); putative MayDay(31–189) sequence (grey underlined). DCN peptide fragments identified from ESI analysis derived from samples of trypsin digested media (blue), size exclusion (yellow) and Tris-Tricine in-gel digestion (green).

Mass spectroscopy methods were employed to identify the ~12 kDa fluorescent protein originating from the co-culture of OFM and macrophages. Samples were prepared from OFM_FITC10_+Mϕ and OFM+Mϕ and analyzed by MS using three preparatory methods; an in solution tryspin digest of the conditioned media; size-exclusion chromatography purification; 1-D Tris-Tricine gel separation (as shown in Fig 4B) followed by in-gel trypsin digest. In each approach samples of both OFM_FITC10_+Mϕ and OFM+Mϕ were analyzed, using the FITC sample to track the protein(s) of interest via fluorescence. ESI MS/MS analysis was conducted on OFM+Mϕ samples only. The MASCOT database was used to identify all identified protein fragments from the three sample preparation methods. Peptides from the ECM protein DCN was consistently identified from the MASCOT search results (S1 File), peptide matches are shown in Fig 5. The in-solution trypsin digestion approach identified multiple DCN peptides spanning much of the protein sequence (‘blue’, Fig 5). The SEC approach identified one peptide from the N-terminal region of DCN (‘yellow’, Fig 5). The sample prepared by Tris-Tricine gel separation and in gel digestion identified a second N-terminal DCN peptide (‘green’, Fig 5).

### MMP12 digestion of DCN generates a bioactive protein

**Fig 6:**
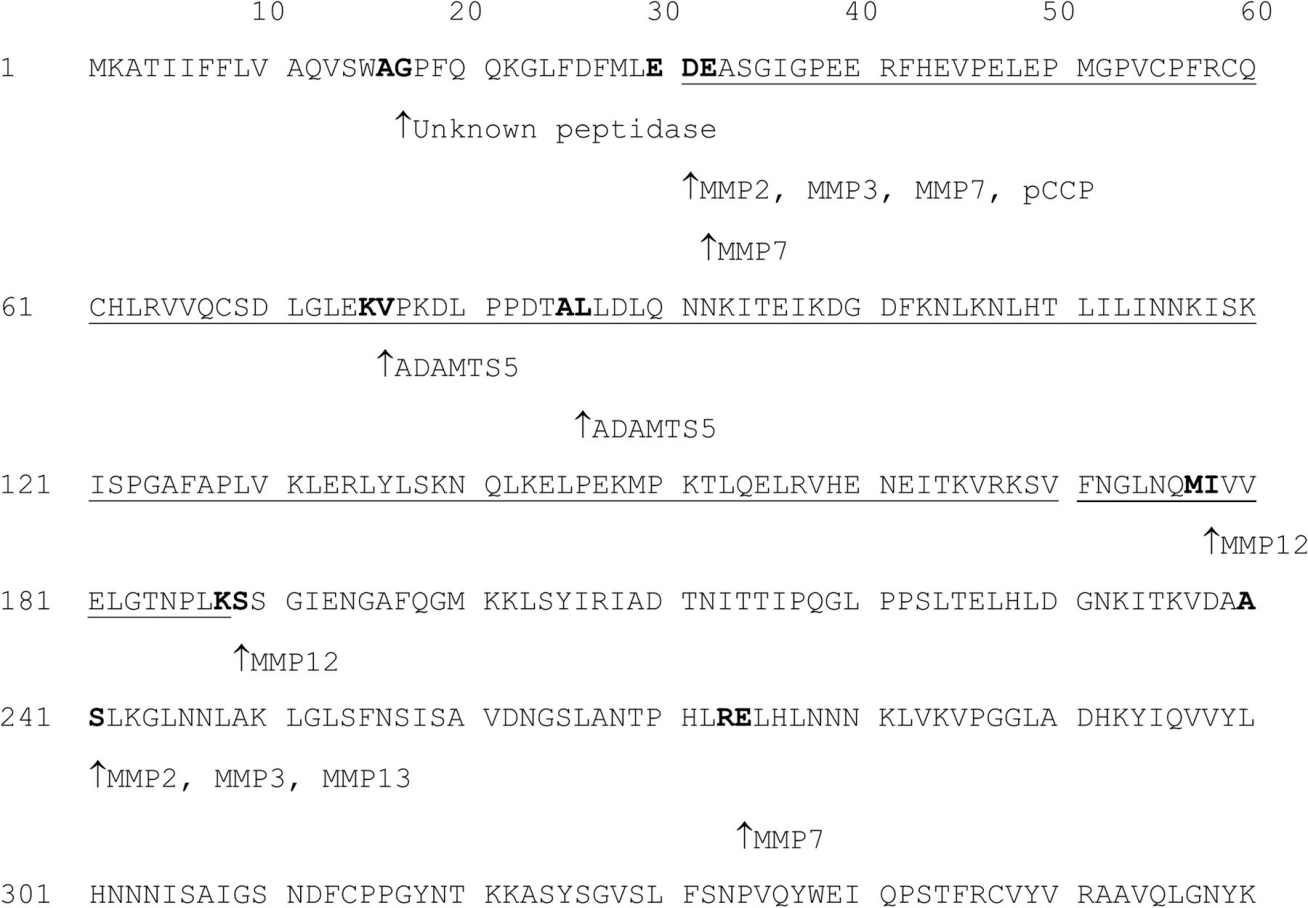
MEROPS computational proteolysis of ovine DCN. Ovine DCN (1–360; accession number: Q9TTE2) (grey); putative MayDay(31–188) sequence (grey underlined). Protease cleavage sites predicted by MEROPS based on the human DCN sequence (1–360; accession number: P07585). Cleavage sites on the DCN sequence are indicated as ‘bold’ text; ‘↑‘ indicates the predicted C-terminal residue of the cleavage site for each indicated protease.

A theoretical proteolytic digest of DCN was conducted *in silico* using the MERPOS database to predict the proteolytic cleavage sites of ovine DCN, based on sequence homology to known human DCN cleavage sites. As shown in Fig 6, DCN contained predicted protease sites for MMP-2, -3, -7, -12 and -13, as well as ADAMTs5. Two MMP-12 sites were predicted to occur between residues 177–178 and 188–189.

**Fig 7:**
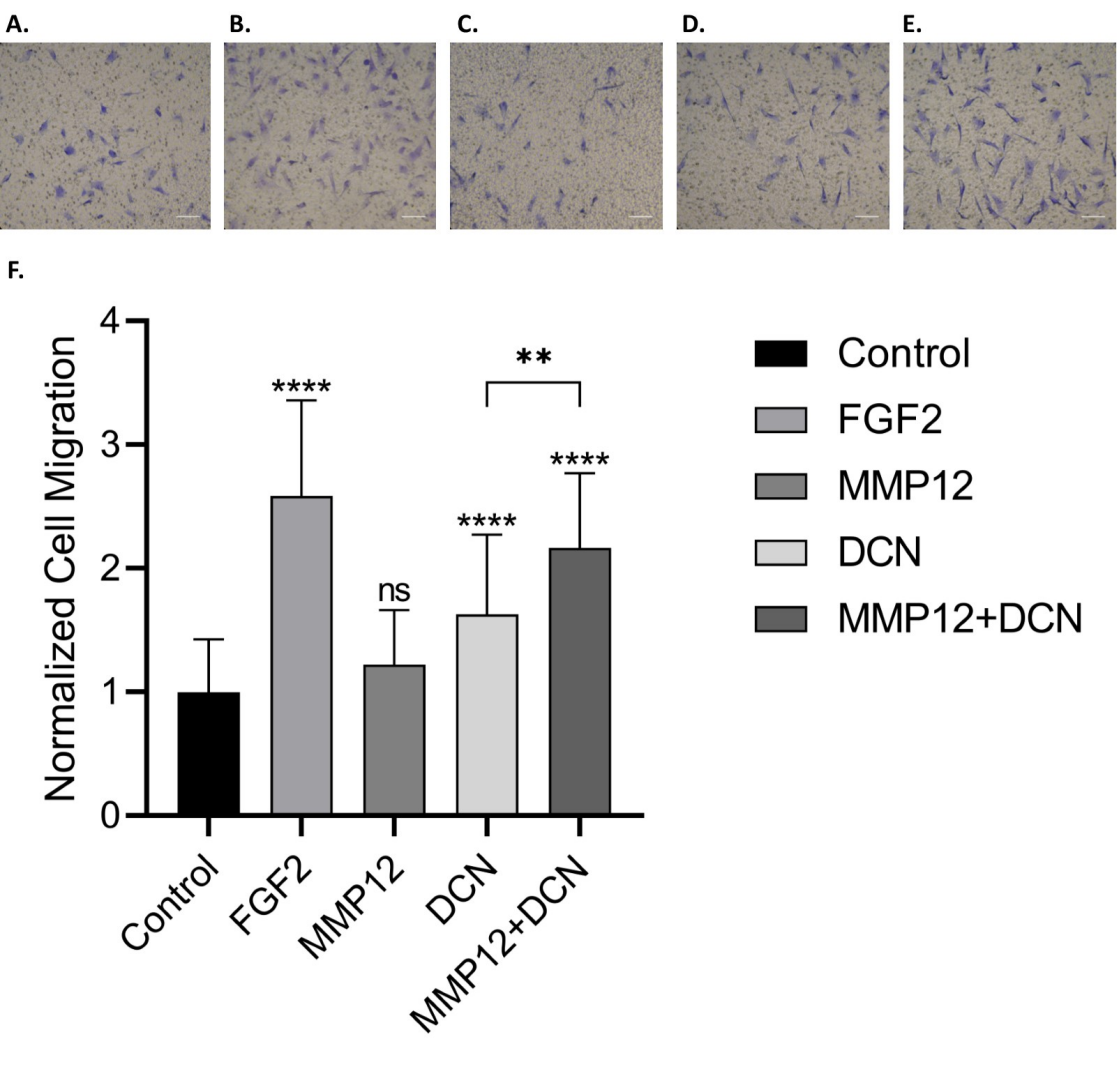
Bioactivity of MMP12 digested DCN in a MSC migration assay. Recombinant human DCN was digested with MMP-12 prior to a transwell migration assay was conducted using ovAD-MSCs. Media alone (‘Control’) and FGF2 (50 ng/mL), included as the positive and negative controls, respectively. Migrated ovAD-MSCs were imaged after 6 h. Representative photomicrographs of test groups are included in panels A through E (A = media control; B = FGF2 (50 ng/mL); C = MMP12; D = DCN; E = MMP12+DCN). Cell migration was quantified and results expressed as the average cell migration normalized to the media control (‘Normalized Cell Migration’) (F). Error bars represent standard deviation from three independent experiments. Statistical significance was determined via t-test, where; ‘*’p ≤ 0.05 ‘**’p ≤ 0.01 ‘***’p ≤ 0.001; ‘****’p ≤ 0.000.

Studies were conducted to assess the bioactivity of DCN fragments resulting from MMP12 digestion. Recombinant human DCN was digested with MMP-12 and digestion confirmed via Bis-Tris gel electrophoresis (S2 File). Samples of MMP-12 digested DCN, MMP12 and DCN were assayed for chemotactic activity using the ovAD-MSC transwell migration assay (Fig 7). The relative cell migration of ovAD-MSC increased with MMP12 digested DCN (2.16±0.60), compared with undigested DCN (1.63±0.64), and MMP-12 alone (1.22±0.44).

### *In vitro* bioactivity of recombinant DCN protein fragment

**Fig 8:**
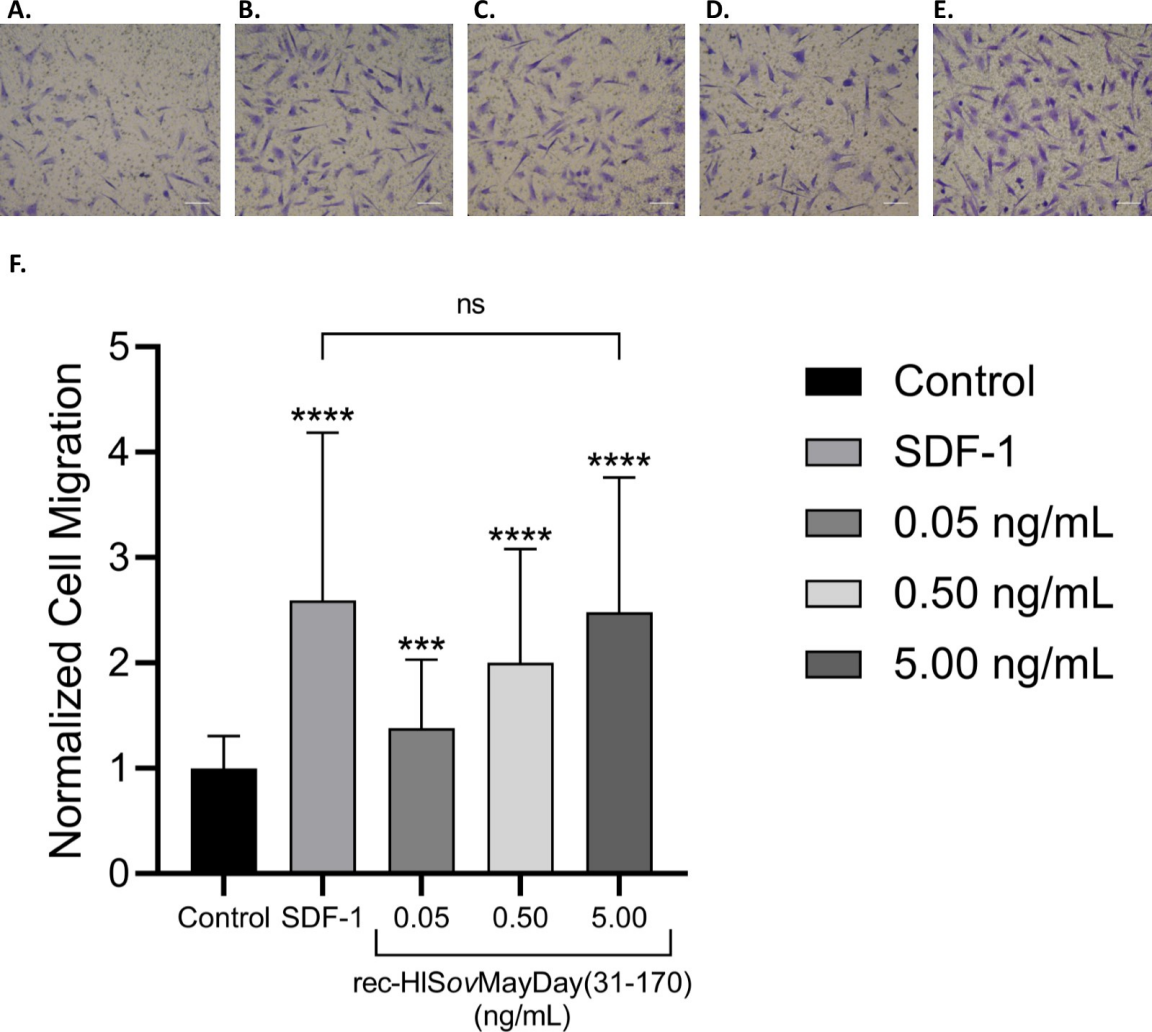
Bioactivity of recombinant MayDay(31–170) in a MSC migration assay. Recombinant HIS-tagged MayDay(31–170) [rec-HISovMayDay(31–170)] was assayed at three concentrations using a transwell assay using ovAD-MSC. Media alone (‘Control’) and SDF-1 (50 ng/mL), included as the positive and negative controls, respectively. Migrated ovAD-MSCs were imaged after 6 h. Representative photomicrographs of test groups are included in panels A through E (A = media control; B = SDF-1 (50 ng/mL); C = 0.05 ng/mL; D = 0.50 ng/mL; E = 5.00 ng/mL). Cell migration was quantified and expressed as the average cell migration normalized to the media control (‘Normalized Cell Migration’) (F). Error bars represent standard deviation from three independent experiments. Statistical significance was determined via t-test, where; ‘*’p ≤ 0.05 ‘**’p ≤ 0.01 ‘***’p ≤ 0.001; ‘****’p ≤ 0.000.

The N-terminal recombinant protein sequence of ovine DCN, terminating at residue 170 were expressed and purified (rec-HIS*ov*MayDay(31–170)). The recombinant protein was assayed for chemotactic activity in the ovAD-MSC transwell migration assay (Fig 8). The known MSC chemotactic agent SDF-1 (50 ng/mL) was included as a positive control. Purified rec-HIS*ov*MayDay(31–170) was bioactive in a dose dose dependent manner. The chemotactic activity of rec-HIS*ov*MayDay(31–170) at 5.00 ng/mL was equivalent to the bioactivity of the positive control SDF-1 at 50 ng/mL.

### *In vivo* bioactivity of recombinant DCN protein fragment

Bone marrow-derived cells (muBM-MSCs) were isolated and expanded from Balb/Cmice. The multipotency of the isolated muBM-MSC was verified by tri-lineage differentiation assay (osteogenesis, adipogenesis, and chondrogenesis) (S2 Fig). Additionally, muBM-MSC were shown to be positive for the MSC markers; CD29, CD105, CD106, CD44, CD73, Sca1 via FACS analysis (S3 Fig).

**Fig 9:**
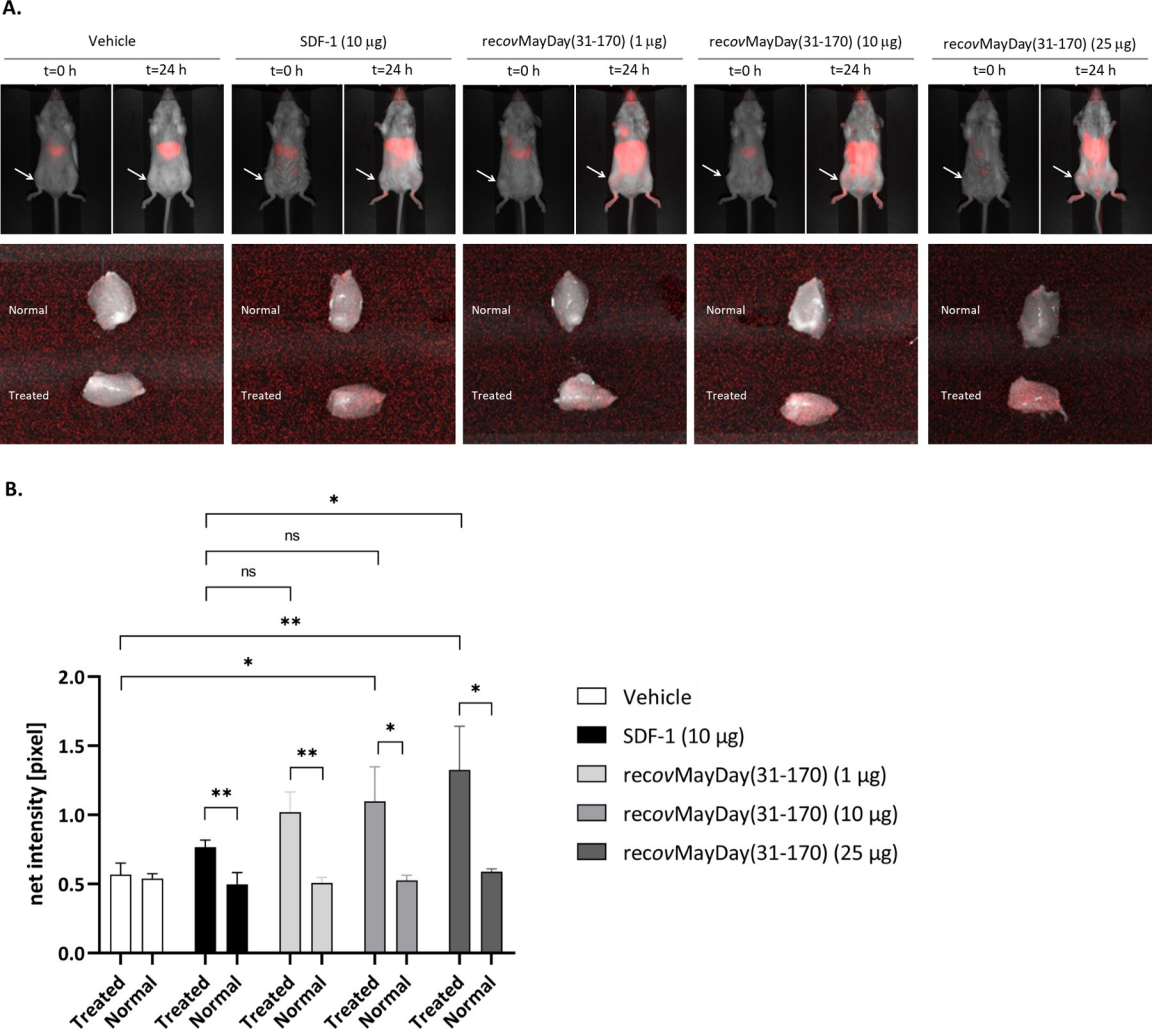
Bioactivity of recombinant MayDay(31–170) in an *in vivo* model of MSC recruitment. A. Representative images of animals from each of the treatment groups at t = 0 and t = 24 h post injection of labelled muBM-MSC. Arrows indicate the injection site for each of the treatment groups. Representation images of excised ‘normal’ and ‘treated’ muscle tissue. B. Quantification of fluorescence intensity (pixels) of excised ‘normal’ and ‘treated’ muscle tissue for each of the treatment groups. Error bars represent standard error form triplicate animals. Statistical significance was determined via t-test, where; ‘*’p ≤ 0.05 ‘**’p ≤ 0.01 ‘***’p ≤ 0.001; ‘****’p ≤ 0.000.

For the *in vivo* MSC recruitment assay the tag-free protein, rec-*ov*MayDay(31–170), was expressed. Labelled muBM-MSC were then delivered to recipient Balb/C mice via tail vein injection then animals were injected with an intramuscular dose of rec-*ov*MayDay(31–170) (1, 10 or 25 μg) or SDF-1 (10 μg). After 24 hours the recruitment of exogenous labelled muBM-MSC cells was quantified by imaging the ‘normal’ and ‘treated’ explanted hind limb. At all concentrations of rec-*ov*MayDay(31–170) tested, a significant increase in the recruitment of muBM-MSC to the injection site was observed (‘normal’ vs ‘treated’, Fig 9), indicating recruitment of muBM-MSC to the site of rec-*ov*MayDay(31–170) administration. Sites receiving higher concentrations of 10 and 25 μg rec-*ov*MayDay(31–170) (‘treated’, Fig 9) showed significantly more localized muBM-MSC’s, relative to sites receiving the vehicle control. The highest dose of rec-*ov*MayDay(31–170) (25 μg) lead to significantly more muBM-MSC localization than the positive control, SDF-1 at 10 μg. Whole animal images showed that the majority of labelled muBM-MSC cells appeared to be localized to the lungs at t = 0, then diffused to extremities and abdominal organs at t = 24 h.

## Discussion

Studies have shown that dECM biomaterials have a range of biological properties and are capable of recruiting MSCs, a feature that may in part explain their clinical performance in soft tissue repair [36, 37, 45]. OFM has previously been shown to stimulate HUVEC cell migration, proliferation and angiogenesis *in vitro* [25], and the current findings support the conclusion that OFM itself stimulates MSC chemotaxis, a biological property that has not previously been described. dECM biomaterials undergo proteolytic digestion once implanted and several studies have shown that chemical or enzymatic breakdown of dECM biomaterials produces novel or hidden cryptic ECM molecules and can modify the biological properties of dECMs [46–49], including the recruitment of MSCs [40]. For example, protein S100-A7 derived from MMP-20 digested dentin matrix was shown to recruit CD146 positive cells to the site of wounded dental pulp in a rat model [50]. A collagen III-derived peptide isolated from a dECM by proteolytic degradation was shown to be chemotactic towards human cortical neural stem cells, adipocyte stem cells, myoblast cells and Schwann cells *in vitro* and *in vivo* [45, 48].

**Fig 10:**
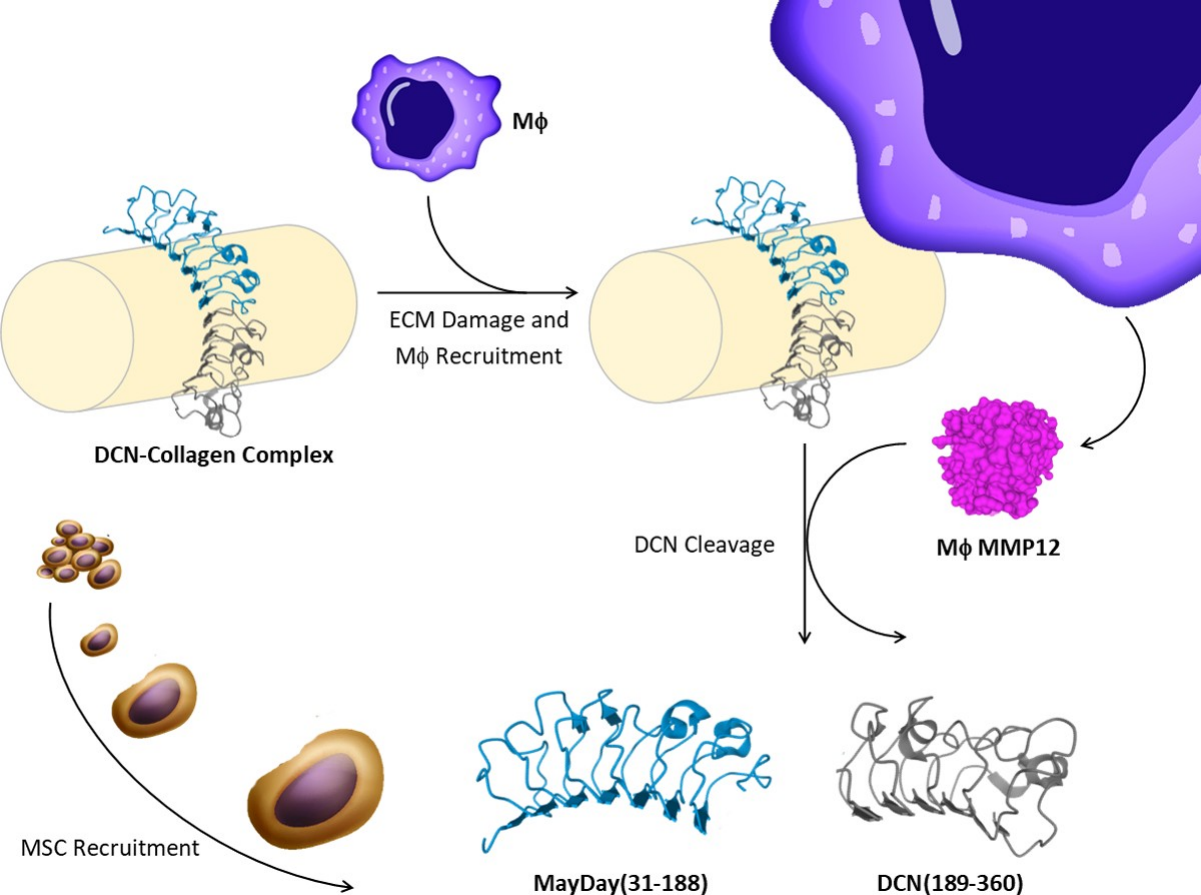
Proposed mechanism. When the extracellular matrix is intact, DCN protein is associated with collagen I, uniformly interspersed along fibrils. During tissue damage or injection, macrophages (Mϕ) are recruited to the site of damage. M2 macrophage cells express MMP-12 which cleaves DCN, close to the collagen binding site. DCN is now uncoupled from collagen and free to diffuse to surrounding tissue and blood stream. The N-terminal DCN fragment, putatively MayDay(31–188), is now free to act as a chemotactic factor for mesenchymal stromal cells, drawing them towards the site of tissue damage.

Rather than using proteolytic processing of ECM our approach used a combination of RAW macrophage cells and the dECM OFM. This strategy identified a novel dominant protein fragment, putatively MayDay(31–188) originating from the N-terminal of DCN. *In vitro* and *in vivo* assays show that recombinant MayDay(31–170) is chemotactic toward cultured MSCs, and suggests that DCN and DCN cleavage may play an important role in MSC recruitment following macrophage mediated degradation of the ECM (Fig 10). DCN belongs to the small leucine-rich (LRR) proteoglycan family that binds to collagens and growth factors [51–54]. DCN has been shown to affect multiple cellular functions such as differentiation, proliferation, migration, cell spreading and inflammation [55–57]. In relation to soft tissue repair, DCN has demonstrated roles in collagen fibrogenesis [58, 59], modulating TGFβ [60], scar formation [61] and inflammatory reactions during contact dermatitis [62, 63]. The DCN sequence of interest, spanning amino acids 31–188, is known to play a role in collagen binding; LRR 4–6 are important for binding of DCN to collagen and receptor tyrosine kinases, including VEGFR2 and EGFR [64, 65]. Putative collagen binding sites residues 101–104 (RELK) and 243–246 (RELH) are located within the concave face of DCN in LRRs 3–4 and 10 respectively [66]. A review by Gubiotti *et al*. stipulates that while the DCN is in a complex with collagen other DCN binding sites are unavailable to cell receptors, and that unbound monomeric DCN is able to act as a paracrine factor [64]. However, the authors of this review note that many of the ligand/DCN interfaces remain uncharacterized.

Macrophages were chosen for the current studies as they are known as initial responders to tissue damage, and furthermore dECM biomaterials lead to a predominantly M2 population of macrophage cells [67]. M2 macrophages are known to continuously express MMP12 [68], and the predicted MMP12 proteolysis of DCN would result in cleavage at amino acids 177–178 and 188–189 giving rise to two smaller proteins of ~10–20 kDa. The putative N-terminal DCN fragment identified in this study, MayDay(31–188), would include the first and second DCN domains, as well as the first 5 LRRs of DCN. It is unknown whether digestion by MMP-12 occurs at the 177–178 or 188–189 cleavage sites of DCN, however the activity of recombinant MayDay(31–170) indicates that the activity is likely the result of an epitope located in the first 5 LLRs of DCN. Studies are ongoing to further elaborate the C-terminus of MayDay in the DCN sequence, as well as, the MayDay(31–188) receptor.

There is tremendous interest in endogenous MSC recruitment as an alternative to *ex vivo* culture of autologous MSCs. Based on our findings it is hypothesized that the administration of the putative MayDay protein, or an analogue thereof may have therapeutic applications in the recruitment of MSCs to damaged or diseased tissue. SDF-1 is possibly the best studied chemotactic agent in this class, having been evaluated for the treatment of renal ischemia [69] and ischemic cardiomyopathy [70], traumatic brain injury [71] and the repair of cognitive ability and cortical dendritic spine rescue [72]. In human studies, a gene therapy trial overexpressed SDF-1 in patients with ischemic heart disease, demonstrated improvements to patient outcomes [73]. Clinical trials with BL-8040 and G-CSF (a glycoprotein that induces stem cell mobilization by decreasing bone marrow SDF-1 and up-regulating CXCR4) [74] have shown a significant increase in CD34+ cell mobilization from the bone marrow for patients that require autologous transplant in myeloma [75]. Direct injection of SDF-1 demonstrated increased neovascularization in skeletal muscle and myocardium [76, 77]. Other delivery methods have been explored including the incorporation of SDF-1 with a collagen sponge to improve tendon repair in a rat model of Achilles tendon repair [78]. A protease resistant version of SDF-1 injection was shown to increase the half-life of SDF-1 and improve myocardial infarction recovery [79]. Further *in vivo* studies are needed to assess the therapeutic potential of MayDay in soft tissue regeneration.

The use of serum-free media and studies using recombinant MayDay(31–170) and MMP12 digested DCN support the conclusion that the observed bioactivity was not an artifact. However, one limitation to the study is the use of *in vitro* isolated MSCs as a model for progenitor cell recruitment. In this study we refer to the mu-BM-MSCs and ovAD-MSC cells as ‘mesenchymal stromal cells’ because they have been cultured *in vitro* and are no longer part of the stem cell niche found in bone marrow or adipose tissue. While these cultured cells are a useful model for cell recruitment *in vivo* and *in vitro*, they may not necessarily recapitulate the action of cells from the stem cell niche. A further exploration of the effect of MayDay(31–188) on endogenous stem cell populations in an *in vivo* model is required.

The discovery platform described herein could theoretically be modified to include alternate dECM biomaterials, for instance nerve-, CNS-, muscle- or tumor-derived dECM, isolated from a variety of mammalian or non-mammalian sources [80]. In parallel, a wide variety of cell types could be used as tools for dECM breakdown. Here we conducted a biologically directed isolation utilizing a MSC chemotaxis assay, but other relevant assays could be incorporated (e.g. bacteriocidal, tumorogenesis, collagen synthesis) to guide identification of novel bioactive ECM components of interest.

## Conclusions

This work presents a platform for the discovery of ECM-derived bioactive products. Using this platform, a novel mechanism has been demonstrated for MSC homing resulting from the breakdown of the ECM component DCN by macrophage cells. To our knowledge, this mechanism has not been described previously and may represent a new discovery in constructive tissue remodeling processes and a new functional mechanism of DCN that complements many previous studies demonstrating the structural and paracrine functions of the DCN protein. The use of this novel DCN fragment which we have termed MayDay, may be a therapeutic tool for regenerative medicine technologies towards a number of pathological conditions such as arthritis, myocardial infarction and nerve tissue regeneration where the recruitment of MSCs may benefit patient outcomes. While the extent of the dynamic and reciprocal relationship between cell and the ECM is not fully understood, the described discovery platform may be utilized to identify other ECM-derived bioactive factors and to help elucidate the undiscovered roles of the ECM.

## Supporting information

S1 Fig ovAD-MSC differentiation. *In vitro* culture of ovAD-MSCs; chondrogenic media and stained with Toluidine Blue (A), osteogenic media and stained with Alizarin Red (B), and adipogenic media and stained with Oil Red O (C). Scale = 100 μm.
(TIF)

S2 Fig muBM-MSC differentiation. *In vitro* culture of muBM-MSCs; chondrogenic media and stained with Alcian Blue (A), osteogenic media and stained with Alizarin Red (B), and adipogenic media and Lipidtoxgreen (C). Scale = 100 μm.
(TIF)

S3 Fig Flow cytometry characterization. muBM-MSC cells at passage three were characterized with a mouse mesenchymal marker antibody panel including CD11 and CD45 (A) and CD106, CD105, CD73, Sca1, CD29 and CD44 (B). Red lines indicate cells stained with specific antibody, black lines indicate isotype control and grey line indicates unstained control.
(TIF)

S4 Fig Recombinant protein sequences. Recombinant protein sequences for *E*. *coli* expressed experimental proteins; rec-HIS*ov*MayDay(31–170) and rec-*ov*MayDay(31–170). Amino acid sequences are 148 and 143 residues in length respectively.
(TIF)

S1 File MASCOT sequence analysis. MASCOT Analysis resulting from ESI MS/MS analysis of conditioned media samples prepared using; in solution trypsin digestion, Tris-Tricine SDS-PAGE 1D-gel excision and SEC.
(PDF)

S2 File Raw gel images. Uncropped SDS-PAGE gels used in this manuscript.
(PDF)
