# Correction: A novel chemotactic factor derived from the extracellular matrix protein decorin recruits mesenchymal stromal cells *in vitro* and *in vivo*

**DOI:** 10.1371/journal.pone.0238964

**Published:** 2020-09-03

**Authors:** Sandi G. Dempsey, Christopher H. Miller, Julia Schueler, Robert W. F. Veale, Darren J. Day, Barnaby C. H. May

The first and second authors’ names are incorrect. The correct names are: Sandi G. Dempsey and Christopher H. Miller. The correct citation is: Dempsey SG, Miller CH, Schueler J, Veale RWF, Day DJ, May BCH (2020) A novel chemotactic factor derived from the extracellular matrix protein decorin recruits mesenchymal stromal cells *in vitro* and *in vivo*. PLoS ONE 15(7): e0235784. https://doi.org/10.1371/journal.pone.0235784.

In the Introduction, there is an error in the third sentence of the fourth paragraph. The correct sentence is: However, an alternate approach is to recruit endogenous MSCs via a chemotactic agent to the site of damage, thus eliminating the time, cost and potential complications that are associated with the isolation and culture of MSCs *ex vivo* [40].
