# Supplementary figures and images for: A novel chemotactic factor derived from the extracellular matrix protein decorin recruits mesenchymal stromal cells in vitro and in vivo
Source: PLoS One. 2020 Jul 13;15(7):e0235784. doi: 10.1371/journal.pone.0235784 (PMC7357784; doi:10.1371/journal.pone.0235784)

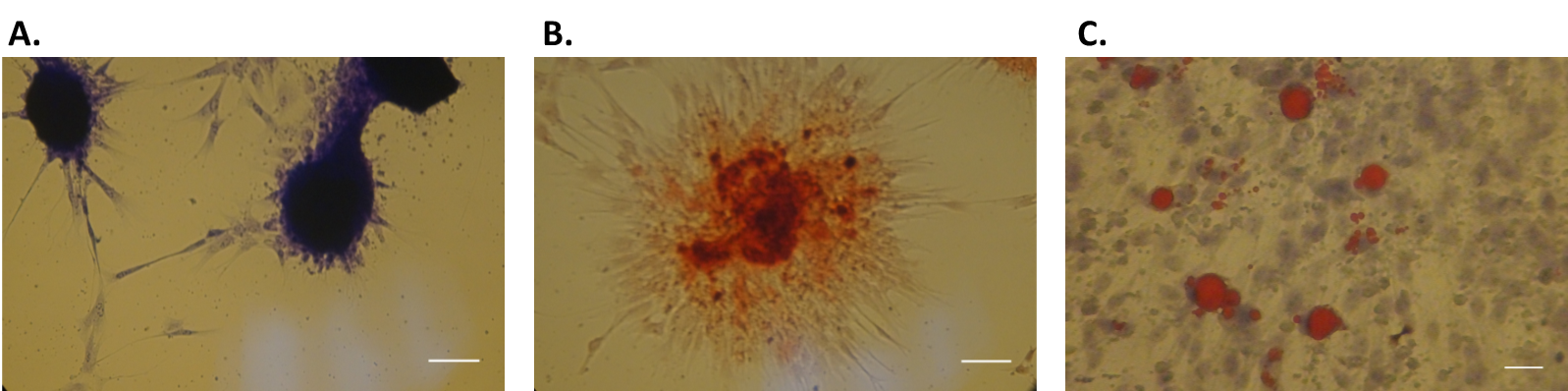

Supplement: S1 Fig — In vitro culture of ovAD-MSCs; chondrogenic media and stained with Toluidine Blue (A), osteogenic media and stained with Alizarin Red (B), and adipogenic media and stained with Oil Red O (C). Scale = 100 μm. (TIF) [file pone.0235784.s001.tif]

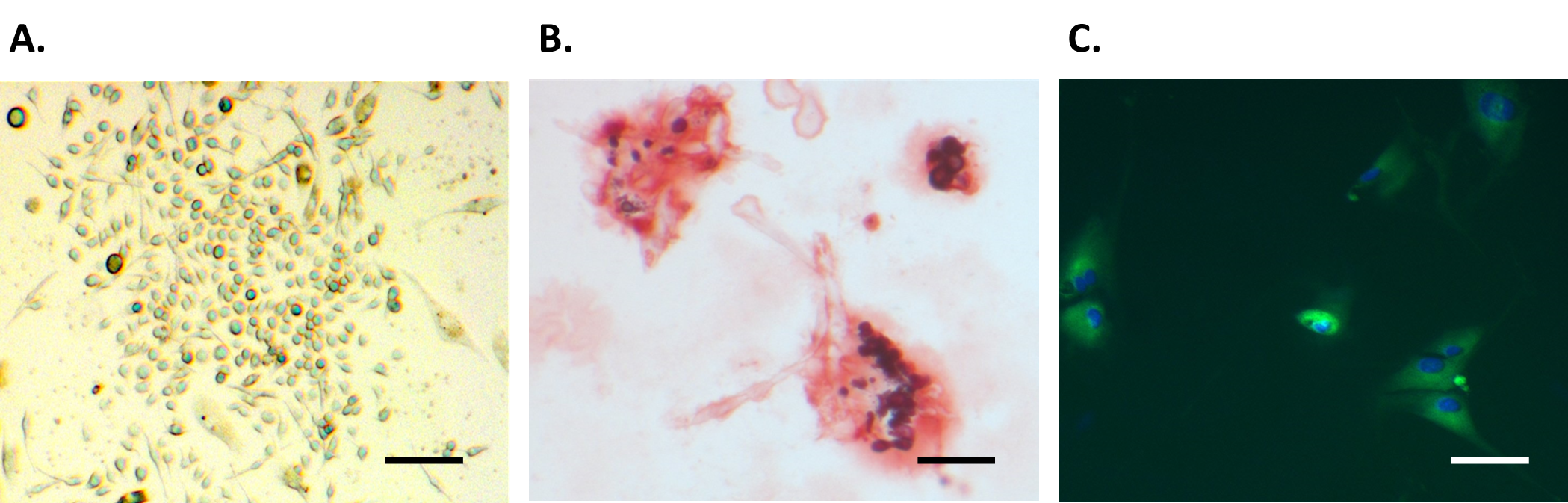

Supplement: S2 Fig — In vitro culture of muBM-MSCs; chondrogenic media and stained with Alcian Blue (A), osteogenic media and stained with Alizarin Red (B), and adipogenic media and Lipidtoxgreen (C). Scale = 100 μm. (TIF) [file pone.0235784.s002.tif]

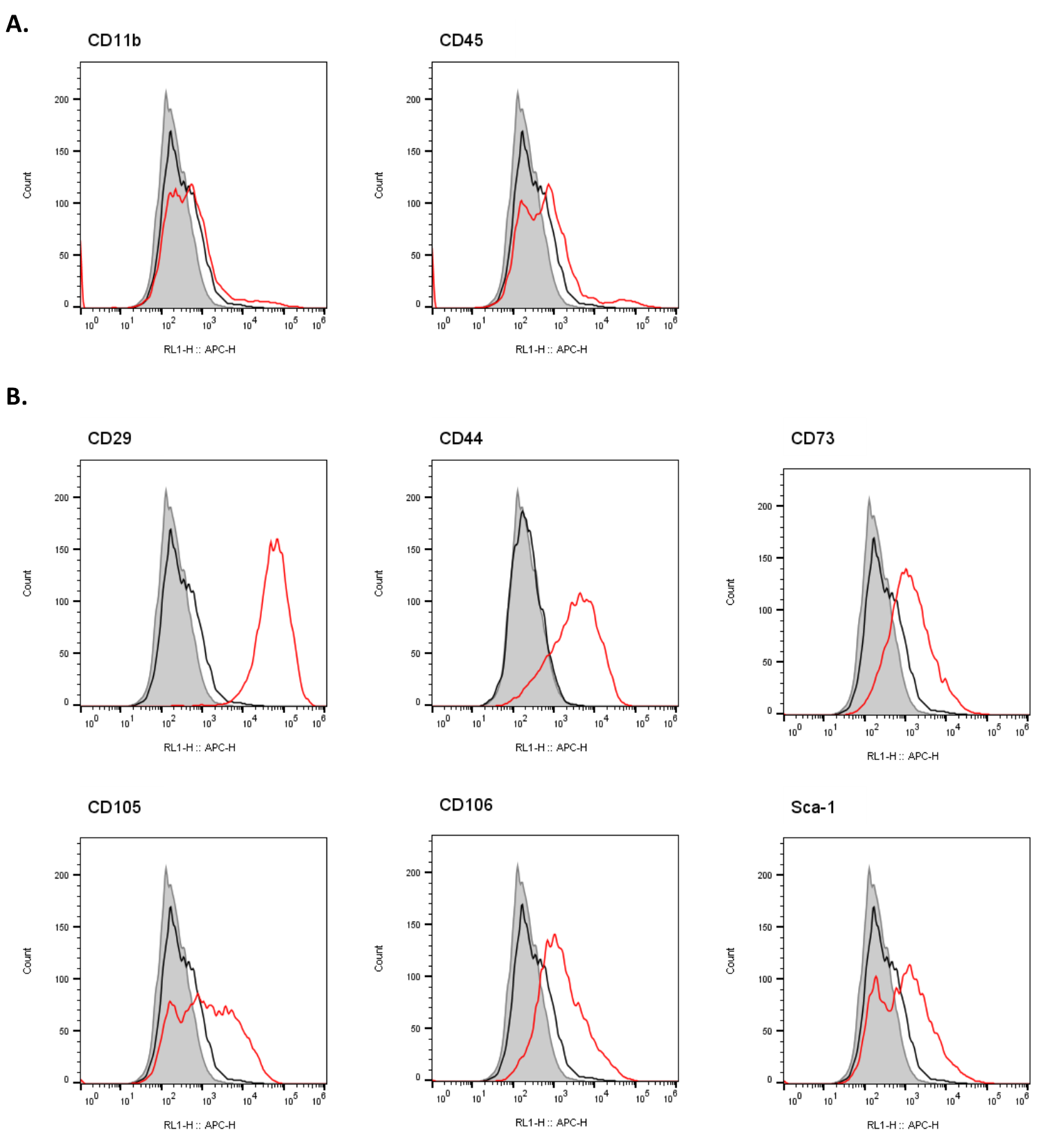

Supplement: S3 Fig — muBM-MSC cells at passage three were characterized with a mouse mesenchymal marker antibody panel including CD11 and CD45 (A) and CD106, CD105, CD73, Sca1, CD29 and CD44 (B). Red lines indicate cells stained with specific antibody, black lines indicate isotype control and grey line indicates unstained control. (TIF) [file pone.0235784.s003.tif]

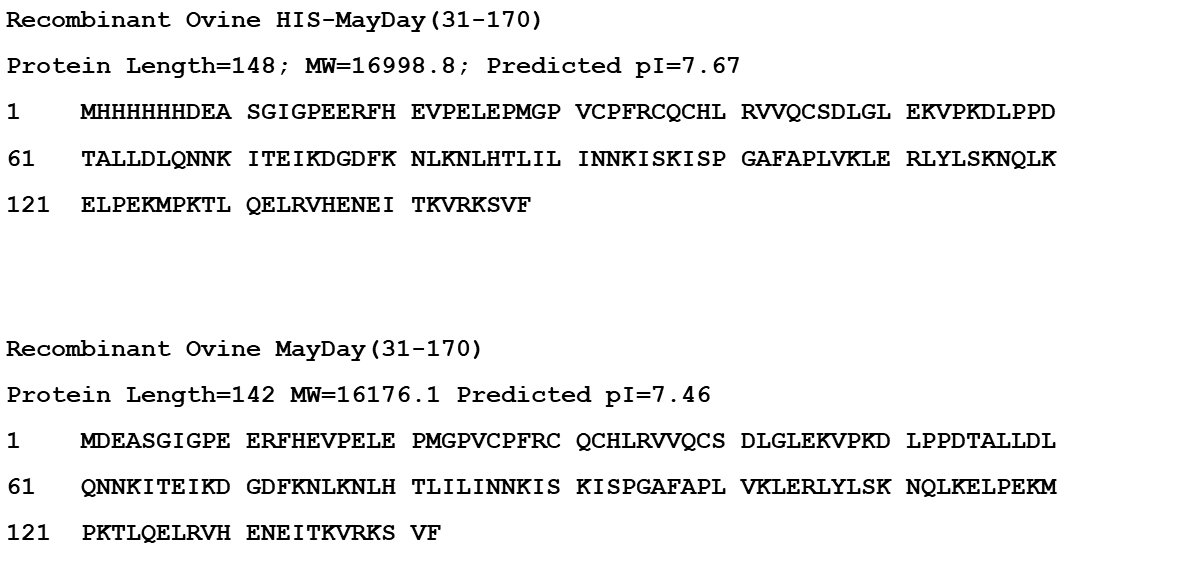

Supplement: S4 Fig — Recombinant protein sequences for E. coli expressed experimental proteins; rec-HISovMayDay(31–170) and rec-ovMayDay(31–170). Amino acid sequences are 148 and 143 residues in length respectively. (TIF) [file pone.0235784.s004.tif]
