# Supplementary material for: A novel chemotactic factor derived from the extracellular matrix protein decorin recruits mesenchymal stromal cells in vitro and in vivo
Source: PLoS One. 2020 Jul 13;15(7):e0235784. doi: 10.1371/journal.pone.0235784 (PMC7357784; doi:10.1371/journal.pone.0235784)
Supplement: S1 File — MASCOT Analysis resulting from ESI MS/MS analysis of conditioned media samples prepared using; in solution trypsin digestion, Tris-Tricine SDS-PAGE 1D-gel excision and SEC. (PDF) [file pone.0235784.s005.pdf]

S4 Fig MASCOT Search Results – Trypsin Sample

Mascot Search Results

```
User         :
Email        :
Search title : Submitted from Extraction 17432 by Mascot Daemon on APAF-WS-08
MS data file : \\apaf-hpv-file\projects\External\e_17432_VicUniWell_SandiDempsey_20150209\1_MassSpec\5600\Run1\Results\150213_P17432_SD1096-1_1.MGF
Database     : sp_sheep_140625 sheep_140625_ (647 sequences; 258179 residues)
Timestamp    : 15 Feb 2015 at 21:52:32 GMT
Protein hits : Q9TTE2 sp|Q9TTE2|PGS2_SHEEP Decorin OS=Ovis aries GN=DCN PE=2 SV=1
               P60713 sp|P60713|ACTB_SHEEP Actin, cytoplasmic 1 OS=Ovis aries GN=ACTB PE=2 SV=1
               Q9MZA9 sp|Q9MZA9|VIME_SHEEP Vimentin (Fragment) OS=Ovis aries GN=VIM PE=2 SV=3
               O77727 sp|O77727|K1C15_SHEEP Keratin, type I cytoskeletal 15 OS=Ovis aries GN=KRT15 PE=2 SV=1
               O46390 sp|O46390|PGS1_SHEEP Biglycan OS=Ovis aries GN=BGN PE=2 SV=1
               Q6B7M7 sp|Q6B7M7|COF1_SHEEP Cofilin-1 OS=Ovis aries GN=CFL1 PE=2 SV=3
               P68251 sp|P68251|1433B_SHEEP 14-3-3 protein beta/alpha (Fragments) OS=Ovis aries GN=YWHAB PE=1 SV=2
               Q6YNC8 sp|Q6YNC8|H2AZ_SHEEP Histone H2A.Z OS=Ovis aries GN=H2AFZ PE=2 SV=3
               P62896 sp|P62896|CYC_SHEEP Cytochrome c OS=Ovis aries GN=CYCS PE=1 SV=2
               Q9BGM5 sp|Q9BGM5|K1C25_SHEEP Keratin, type I cytoskeletal 25 OS=Ovis aries GN=KRT25 PE=2 SV=1
               Q28554 sp|Q28554|G3P_SHEEP Glyceraldehyde-3-phosphate dehydrogenase (Fragment) OS=Ovis aries GN=GAPDH PE=2 SV=4
               P15241 sp|P15241|K2M2_SHEEP Keratin, type II microfibrillar, component 7C OS=Ovis aries PE=1 SV=1
               P21814 sp|P21814|UTMP_SHEEP Uterine milk protein OS=Ovis aries PE=1 SV=2
               Q83957 sp|Q83957|NCAP_ORSVW Nucleoprotein OS=Ovine respiratory syncytial virus (strain WSU 83-1578) GN=N PE=2 SV=1
               P01318 sp|P01318|INS_SHEEP Insulin OS=Ovis aries GN=INS PE=1 SV=2
               P03370 sp|P03370|POL_VILV Pol polyprotein OS=Maedi visna virus (strain 1514) GN=pol PE=3 SV=1
               P0C276 sp|P0C276|RL40_SHEEP Ubiquitin-60S ribosomal protein L40 OS=Ovis aries GN=UBA52 PE=2 SV=2
```

	sp_sheep_140625	Decoy	False discovery rate
Peptide matches above identity threshold	104	3	2.88 %
Peptide matches above homology or identity threshold	104	3	2.88 %

Mascot Score Histogram

Ions score is -10*Log(P), where P is the probability that the observed match is a random event.
Individual ions scores > 19 indicate identity or extensive homology (p<0.01).
Protein scores are derived from ions scores as a non-probabilistic basis for ranking protein hits.

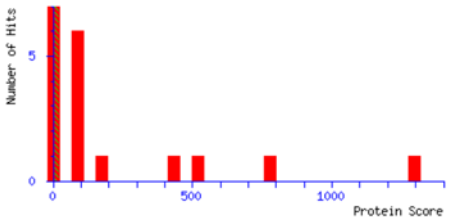

Peptide Summary Report

Format As: Peptide Summary | Help

Significance threshold p< 0.01 | Max. number of hits AUTO | Show Percolator scores

Standard scoring | MudPIT scoring | Ions score or expect cut-off 19 | Show sub-sets 0

Show pop-ups | Suppress pop-ups | Sort unassigned Decreasing Score | Require bold red

Select All | Select None | Search Selected | Error tolerant | Archive Report

1. Q9TTE2 **Mass:** 39947 **Score:** 1295 **Matches:** 30(30) **Sequences:** 5(5) **emPAI:** 0.89
sp|Q9TTE2|PGS2_SHEEP Decorin OS=Ovis aries GN=DCN PE=2 SV=1
Check to include this hit in error tolerant search or archive report

Query	Observed	Mr(expt)	Mr(calc)	ppm	Miss	Score	Expect	Rank	Unique	Peptide
476	550.3283	1098.6420	1098.6437	-1.56	0	(59)	1.3e-006	1	U	K.ISPGAFAPLVK.L
477	550.3284	1098.6423	1098.6437	-1.27	0	(65)	2.8e-007	1	U	K.ISPGAFAPLVK.L
478	550.3287	1098.6428	1098.6437	-0.83	0	(51)	8.2e-006	1	U	K.ISPGAFAPLVK.L
479	550.3288	1098.6430	1098.6437	-0.67	0	66	2.5e-007	1	U	K.ISPGAFAPLVK.L
586	389.2077	1164.6014	1164.6026	-1.10	1	(46)	2.5e-005	1	U	K.ITEIKDGDFK.N
587	389.2077	1164.6014	1164.6026	-1.08	1	(49)	1.3e-005	1	U	K.ITEIKDGDFK.N
588	389.2078	1164.6017	1164.6026	-0.84	1	(48)	1.8e-005	1	U	K.ITEIKDGDFK.N
589	583.3082	1164.6018	1164.6026	-0.69	1	(57)	2e-006	1	U	K.ITEIKDGDFK.N
590	583.3082	1164.6018	1164.6026	-0.69	1	61	8.3e-007	1	U	K.ITEIKDGDFK.N
591	583.3082	1164.6018	1164.6026	-0.69	1	(57)	2e-006	1	U	K.ITEIKDGDFK.N
592	583.3085	1164.6024	1164.6026	-0.20	1	(55)	3e-006	1	U	K.ITEIKDGDFK.N
593	583.3085	1164.6024	1164.6026	-0.20	1	(59)	1.3e-006	1	U	K.ITEIKDGDFK.N
594	583.3085	1164.6024	1164.6026	-0.20	1	(56)	2.7e-006	1	U	K.ITEIKDGDFK.N
595	583.3085	1164.6024	1164.6026	-0.20	1	(54)	4e-006	1	U	K.ITEIKDGDFK.N
596	583.3085	1164.6024	1164.6026	-0.20	1	(58)	1.8e-006	1	U	K.ITEIKDGDFK.N
597	389.2081	1164.6024	1164.6026	-0.20	1	(58)	1.7e-006	1	U	K.ITEIKDGDFK.N
598	389.2082	1164.6028	1164.6026	0.16	1	(51)	9e-006	1	U	K.ITEIKDGDFK.N
797	431.5934	1291.7584	1291.7612	-2.15	0	73	5.3e-008	1	U	K.NLHTLILINNK.I
798	646.8870	1291.7595	1291.7612	-1.28	0	(53)	5.5e-006	1	U	K.NLHTLILINNK.I
799	646.8878	1291.7610	1291.7612	-0.17	0	(48)	1.7e-005	1	U	K.NLHTLILINNK.I
800	646.8878	1291.7610	1291.7612	-0.17	0	(52)	6.3e-006	1	U	K.NLHTLILINNK.I
154	431.5948	1291.7626	1291.7612	1.10	0	(36)	0.00026	1	U	K.NLHTLILINNK.I
1205	833.9335	1665.8524	1665.8573	-2.96	0	99	1.2e-010	1	U	K.DLPPDTALLDLQNNK.I
1206	833.9335	1665.8524	1665.8573	-2.96	0	(97)	2.4e-010	1	U	K.DLPPDTALLDLQNNK.I
1207	833.9335	1665.8524	1665.8573	-2.96	0	(87)	2e-009	1	U	K.DLPPDTALLDLQNNK.I
1208	833.9335	1665.8524	1665.8573	-2.94	0	(79)	1.4e-008	1	U	K.DLPPDTALLDLQNNK.I
1209	833.9335	1665.8524	1665.8573	-2.94	0	(94)	4.2e-010	1	U	K.DLPPDTALLDLQNNK.I
1210	556.2929	1665.8567	1665.8573	-0.37	0	(61)	9.3e-007	1	U	K.DLPPDTALLDLQNNK.I
1211	556.2929	1665.8568	1665.8573	-0.34	0	(94)	4.2e-010	1	U	K.DLPPDTALLDLQNNK.I
1460	664.3621	1990.0644	1990.0735	-4.54	1	52	6.1e-006	1	U	K.VPKDLPPDTALLDLQNNK.I

2. P60713 **Mass:** 41710 **Score:** 797 **Matches:** 23(23) **Sequences:** 7(7) **emPAI:** 0.84
sp|P60713|ACTB_SHEEP Actin, cytoplasmic 1 OS=Ovis aries GN=ACTB PE=2 SV=1
Check to include this hit in error tolerant search or archive report

Query	Observed	Mr(expt)	Mr(calc)	ppm	Miss	Score	Expect	Rank	Unique	Peptide
78	398.2388	794.4630	794.4650	-2.50	0	(32)	0.00064	1	U	K.IIAPPER.K
79	398.2388	794.4630	794.4650	-2.47	0	(29)	0.0013	1	U	K.IIAPPER.K
80	398.2389	794.4633	794.4650	-2.20	0	40	9.2e-005	1	U	K.IIAPPER.K
535	566.7669	1131.5193	1131.5197	-0.27	0	45	3.1e-005	1	U	R.GYSFTTTAER.E
536	566.7671	1131.5196	1131.5197	-0.00	0	(42)	7e-005	1	U	R.GYSFTTTAER.E
537	566.7672	1131.5198	1131.5197	0.14	0	(44)	4.2e-005	1	U	R.GYSFTTTAER.E
538	566.7685	1131.5224	1131.5197	2.47	0	(43)	5.6e-005	1	U	R.GYSFTTTAER.E
582	581.3102	1160.6059	1160.6111	-4.46	0	37	0.00043	1	U	K.EITALAPSTMK.I
662	400.2392	1197.6958	1197.6982	-2.05	0	45	3.2e-005	1	U	R.AVFPSIVGRPR.H
1097	506.2393	1515.6962	1515.6954	0.54	0	79	1.3e-008	1	U	K.QEYDESGPSIVHR.K
1098	506.2394	1515.6964	1515.6954	0.70	0	(71)	8.4e-008	1	U	K.QEYDESGPSIVHR.K
1365	895.9486	1789.8826	1789.8846	-1.11	0	(67)	2.5e-007	1	U	K.SYELPDGQVITIGNER.F
1366	597.6354	1789.8843	1789.8846	-0.18	0	85	4.3e-009	1	U	K.SYELPDGQVITIGNER.F
1367	597.6354	1789.8843	1789.8846	-0.18	0	(64)	5.3e-007	1	U	K.SYELPDGQVITIGNER.F
1368	597.6354	1789.8843	1789.8846	-0.18	0	(65)	4.1e-007	1	U	K.SYELPDGQVITIGNER.F
1369	597.6355	1789.8848	1789.8846	0.11	0	(72)	7.8e-008	1	U	K.SYELPDGQVITIGNER.F
1370	597.6355	1789.8848	1789.8846	0.11	0	(56)	3.2e-006	1	U	K.SYELPDGQVITIGNER.F
1371	597.6355	1789.8848	1789.8846	0.11	0	(64)	4.8e-007	1	U	K.SYELPDGQVITIGNER.F
1372	597.6355	1789.8848	1789.8846	0.11	0	(63)	6.7e-007	1	U	K.SYELPDGQVITIGNER.F
1373	895.9552	1789.8959	1789.8846	6.30	0	(66)	3e-007	1	U	K.SYELPDGQVITIGNER.F
1431	652.0258	1953.0555	1953.0571	-0.81	0	(42)	5.7e-005	1	U	R.VAPEEHPVLLTEAPLNPK.A
1432	652.0265	1953.0577	1953.0571	0.31	0	(43)	5e-005	1	U	R.VAPEEHPVLLTEAPLNPK.A
1433	652.0271	1953.0595	1953.0571	1.23	0	48	1.6e-005	1	U	R.VAPEEHPVLLTEAPLNPK.A

---

3. Q9MZA9 **Mass:** 17161 **Score:** 524 **Matches:** 13(13) **Sequences:** 7(7) **emPAI:** 3.20
sp|Q9MZA9|VIME_SHEEP Vimentin (Fragment) OS=Ovis aries GN=VIM PE=2 SV=3
Check to include this hit in error tolerant search or archive report

Query	Observed	Mr(expt)	Mr(calc)	ppm	Miss	Score	Expect	Rank	Unique	Peptide
161	435.7210	869.4274	869.4283	-1.00	0	38	0.00016	1	U	R.FANYIDK.V
200	453.7364	905.4583	905.4607	-2.56	0	36	0.00038	1		R.FLEQQNK.I
507	558.2944	1114.5742	1114.5618	11.1	0	38	0.00078	1	U	K.VELQELNDR.F
526	375.8723	1124.5952	1124.5978	-2.31	1	63	4.6e-007	1	U	R.FANYIDKVR.F
599	585.3605	1168.7064	1168.7067	-0.23	0	(48)	1.7e-005	1	U	K.ILLAELEQLK.G
600	585.3605	1168.7065	1168.7067	-0.11	0	59	1.2e-006	1	U	K.ILLAELEQLK.G
601	585.3609	1168.7072	1168.7067	0.47	0	(48)	1.4e-005	1	U	K.ILLAELEQLK.G
1121	770.4561	1538.8975	1538.9032	-3.64	1	(55)	3.5e-006	1	U	K.ILLAELEQLKGQGK.S
1122	513.9738	1538.8995	1538.9032	-2.38	1	(65)	2.9e-007	1	U	K.ILLAELEQLKGQGK.S
1123	513.9741	1538.9005	1538.9032	-1.70	1	81	8.7e-009	1	U	K.ILLAELEQLKGQGK.S
1124	513.9744	1538.9013	1538.9032	-1.20	1	(56)	2.6e-006	1	U	K.ILLAELEQLKGQGK.S
1167	529.9372	1586.7897	1586.7900	-0.21	1	96	3e-010	1	U	R.TNEKVELQELNDR.F
1168	529.9374	1586.7902	1586.7900	0.15	1	(88)	1.9e-009	1	U	R.TNEKVELQELNDR.F

---

4. O77727 **Mass:** 48740 **Score:** 420 **Matches:** 12(12) **Sequences:** 6(6) **emPAI:** 0.48
sp|O77727|K1C15_SHEEP Keratin, type I cytoskeletal 15 OS=Ovis aries GN=KRT15 PE=2 SV=1
Check to include this hit in error tolerant search or archive report

Query	Observed	Mr(expt)	Mr(calc)	ppm	Miss	Score	Expect	Rank	Unique	Peptide
90	404.2027	806.3907	806.3923	-1.87	0	(35)	0.00033	1	U	R.LAADDFR.L
91	404.2034	806.3921	806.3923	-0.14	0	(40)	0.00012	1	U	R.LAADDFR.L
92	404.2034	806.3923	806.3923	0.04	0	44	4.1e-005	1	U	R.LAADDFR.L
101	412.2307	822.4469	822.4487	-2.16	0	(44)	4.3e-005	1	U	R.LASYLEK.V
102	412.2312	822.4477	822.4487	-1.14	0	44	4.1e-005	1	U	R.LASYLEK.V
365	516.3010	1030.5874	1030.5910	-3.48	0	(44)	3.7e-005	1	U	R.VLDELTLTK.T
366	516.3015	1030.5883	1030.5910	-2.59	0	(48)	1.6e-005	1	U	R.VLDELTLTK.T
367	516.3017	1030.5889	1030.5910	-2.09	0	58	1.6e-006	1	U	R.VLDELTLTK.T
385	521.7992	1041.5839	1041.5818	1.99	0	63	4.5e-007	1	U	R.VILEIDNAR.L
387	522.2930	1042.5715	1042.5771	-5.30	0	53	4.5e-006	1	U	K.ILAAAIDNSR.V
816	651.3319	1300.6492	1300.6510	-1.39	0	87	3e-009	1	U	R.ALEEANADLEVK.I
817	651.3321	1300.6496	1300.6510	-1.05	0	(81)	1.2e-008	1	U	R.ALEEANADLEVK.I

---

5. O46390 **Mass:** 41497 **Score:** 172 **Matches:** 4(4) **Sequences:** 2(2) **emPAI:** 0.17
sp|O46390|PGS1_SHEEP Biglycan OS=Ovis aries GN=BGN PE=2 SV=1
Check to include this hit in error tolerant search or archive report

Query	Observed	Mr(expt)	Mr(calc)	ppm	Miss	Score	Expect	Rank	Unique	Peptide
207	455.2732	908.5319	908.5331	-1.37	0	(34)	0.0004	1	U	R.VPAGLPDLK.L
208	455.2735	908.5324	908.5331	-0.80	0	54	4.1e-006	1	U	R.VPAGLPDLK.L
828	656.8760	1311.7375	1311.7398	-1.75	0	(62)	5.8e-007	1	U	K.IQAIELEDLLR.Y
829	656.8766	1311.7387	1311.7398	-0.80	0	81	7.5e-009	1	U	K.IQAIELEDLLR.Y

---

6. Q6B7M7 **Mass:** 18507 **Score:** 109 **Matches:** 2(2) **Sequences:** 2(2) **emPAI:** 0.40
sp|Q6B7M7|COF1_SHEEP Cofilin-1 OS=Ovis aries GN=CFL1 PE=2 SV=3
Check to include this hit in error tolerant search or archive report

Query	Observed	Mr(expt)	Mr(calc)	ppm	Miss	Score	Expect	Rank	Unique	Peptide
866	669.3184	1336.6223	1336.6187	2.69	0	60	1.1e-006	1	U	R.YALYDATYETK.E
868	670.8917	1339.7689	1339.7711	-1.64	0	70	1.1e-007	1	U	K.LGGSAVISLEGKPL.-

---

7. P68251 **Mass:** 22040 **Score:** 87 **Matches:** 3(3) **Sequences:** 2(2) **emPAI:** 0.33
sp|P68251|1433B_SHEEP 14-3-3 protein beta/alpha (Fragments) OS=Ovis aries GN=YWHAB PE=1 SV=2
Check to include this hit in error tolerant search or archive report

Query	Observed	Mr(expt)	Mr(calc)	ppm	Miss	Score	Expect	Rank	Unique	Peptide

197	452.2589	902.5032	902.5073	-4.53	0	43	5.5e-005	1	U	R.VISSIEQK.T
203	454.2652	906.5159	906.5174	-1.69	0	44	3.8e-005	1		R.NLLSVAYK.N
204	454.2652	906.5159	906.5174	-1.69	0	(40)	0.00011	1		R.NLLSVAYK.N

Proteins matching the same set of peptides:
P68253 **Mass:** 17989 **Score:** 87 **Matches:** 3(3) **Sequences:** 2(2)
sp|P68253|1433G_SHEEP 14-3-3 protein gamma (Fragments) OS=Ovis aries GN=YWHAG PE=1 SV=1

8. Q6YNC8 **Mass:** 13545 **Score:** 66 **Matches:** 3(3) **Sequences:** 1(1) **emPAI:** 0.25
sp|Q6YNC8|H2AZ_SHEEP Histone H2A.Z OS=Ovis aries GN=H2AFZ PE=2 SV=3
Check to include this hit in error tolerant search or archive report

Query	Observed	Mr(expt)	Mr(calc)	ppm	Miss	Score	Expect	Rank	Unique	Peptide
242	472.7673	943.5200	943.5240	-4.16	0	(33)	0.00052	1	U	R.AGLQFPVGR.I
243	472.7682	943.5219	943.5240	-2.17	0	48	1.7e-005	1	U	R.AGLQFPVGR.I
244	472.7685	943.5224	943.5240	-1.68	0	(25)	0.0035	1	U	R.AGLQFPVGR.I

9. P62896 **Mass:** 11696 **Score:** 50 **Matches:** 1(1) **Sequences:** 1(1) **emPAI:** 0.30
sp|P62896|CYC_SHEEP Cytochrome c OS=Ovis aries GN=CYCS PE=1 SV=2
Check to include this hit in error tolerant search or archive report

Query	Observed	Mr(expt)	Mr(calc)	ppm	Miss	Score	Expect	Rank	Unique	Peptide
54	390.2125	1167.6156	1167.6149	0.58	0	50	2e-005	1	U	K.TGPNLHGLFGR.K

10. Q9BGM5 **Mass:** 49282 **Score:** 46 **Matches:** 2(2) **Sequences:** 1(1) **emPAI:** 0.14
sp|Q9BGM5|K1C25_SHEEP Keratin, type I cytoskeletal 25 OS=Ovis aries GN=KRT25 PE=2 SV=1
Check to include this hit in error tolerant search or archive report

Query	Observed	Mr(expt)	Mr(calc)	ppm	Miss	Score	Expect	Rank	Unique	Peptide
422	532.8067	1063.5988	1063.5662	30.7	0	(25)	0.0029	1	U	R.LASYLENVR.A
423	355.5408	1063.6006	1063.5662	32.3	0	41	7.9e-005	1	U	R.LASYLENVR.A

11. Q28554 **Mass:** 34710 **Score:** 44 **Matches:** 1(1) **Sequences:** 1(1) **emPAI:** 0.10
sp|Q28554|G3P_SHEEP Glyceraldehyde-3-phosphate dehydrogenase (Fragment) OS=Ovis aries GN=GAPDH PE=2 SV=4
Check to include this hit in error tolerant search or archive report

Query	Observed	Mr(expt)	Mr(calc)	ppm	Miss	Score	Expect	Rank	Unique	Peptide
911	685.3745	1368.7345	1368.7361	-1.18	0	44	3.9e-005	1	U	R.GAAQNIIPASTGAAK.A

12. P15241 **Mass:** 53647 **Score:** 40 **Matches:** 2(2) **Sequences:** 2(2) **emPAI:** 0.13
sp|P15241|K2M2_SHEEP Keratin, type II microfibrillar, component 7C OS=Ovis aries PE=1 SV=1
Check to include this hit in error tolerant search or archive report

Query	Observed	Mr(expt)	Mr(calc)	ppm	Miss	Score	Expect	Rank	Unique	Peptide
200	453.7364	905.4583	905.4607	-2.56	0	36	0.00038	1		R.FLEQQNK.L
756	632.3501	1262.6857	1262.6870	-1.03	0	25	0.0032	1	U	K.LGLDIEIATYR.R

Proteins matching the same set of peptides:
P25691 **Mass:** 55220 **Score:** 40 **Matches:** 2(2) **Sequences:** 2(2)
sp|P25691|K2M3_SHEEP Keratin, type II microfibrillar, component 5 OS=Ovis aries PE=1 SV=1

13. P21814 **Mass:** 49191 **Score:** 30 **Matches:** 4(4) **Sequences:** 1(1) **emPAI:** 0.07
sp|P21814|UTMP_SHEEP Uterine milk protein OS=Ovis aries PE=1 SV=2
Check to include this hit in error tolerant search or archive report

Query	Observed	Mr(expt)	Mr(calc)	ppm	Miss	Score	Expect	Rank	Unique	Peptide
292	492.3159	982.6173	982.5811	36.9	1	(23)	0.0047	1	U	K.ALIAENPKK.N
293	492.3160	982.6175	982.5811	37.0	1	(23)	0.0052	1	U	K.ALIAENPKK.N
294	492.3162	982.6179	982.5811	37.4	1	24	0.0043	1	U	K.ALIAENPKK.N
295	492.3164	982.6183	982.5811	37.9	1	(20)	0.0098	1	U	K.ALIAENPKK.N

14. Q83957 **Mass:** 43394 **Score:** 28 **Matches:** 2(2) **Sequences:** 1(1) **emPAI:** 0.08
sp|Q83957|NCAP_ORSVW Nucleoprotein OS=Ovine respiratory syncytial virus (strain WSU 83-1578) GN=N PE=2 SV=1
Check to include this hit in error tolerant search or archive report

Query	Observed	Mr(expt)	Mr(calc)	ppm	Miss	Score	Expect	Rank	Unique	Peptide
399	524.7745	1047.5345	1047.5019	31.1	1	25	0.0041	1	U	R.QDVNGKEMK.F
401	524.7747	1047.5349	1047.5019	31.5	1	(24)	0.0056	1	U	R.QDVNGKEMK.F

15. P01318 **Score:** 28 **Matches:** 1(1) **Sequences:** 1(1) **emPAI:** 0.30
sp|P01318|INS_SHEEP Insulin OS=Ovis aries GN=INS PE=1 SV=2
Check to include this hit in error tolerant search or archive report

Query	Observed	Mr(expt)	Mr(calc)	ppm	Miss	Score	Expect	Rank	Unique	Peptide
152	430.2201	858.4256	858.4276	-2.34	0	28	0.0017	1	U	R.GFFYTPK.A

16. P03370 **Mass:** 126558 **Score:** 26 **Matches:** 1(1) **Sequences:** 1(1) **emPAI:** 0.03
sp|P03370|POL_VILV Pol polyprotein OS=Maedi visna virus (strain 1514) GN=pol PE=3 SV=1
Check to include this hit in error tolerant search or archive report

Query	Observed	Mr(expt)	Mr(calc)	ppm	Miss	Score	Expect	Rank	Unique	Peptide
902	680.3549	1358.6952	1358.6500	33.3	1	26	0.0049	1	U	R.AIEEACKQGPEK.M + Carbamidomethyl (C)

Proteins matching the same set of peptides:
P16901 **Mass:** 124437 **Score:** 26 **Matches:** 1(1) **Sequences:** 1(1)

sp|P16901|POL_OMVVS Pol polyprotein OS=Ovine maedi visna related virus (strain South Africa) GN=pol PE=3 SV=1
P23426 **Mass:** 126548 **Score:** 26 **Matches:** 1(1) **Sequences:** 1(1)
sp|P23426|POL_VILV1 Pol polyprotein OS=Maedi visna virus (strain 1514 / clone LV1-1KS1) GN=pol PE=3 SV=1
P23427 **Mass:** 126543 **Score:** 26 **Matches:** 1(1) **Sequences:** 1(1)
sp|P23427|POL_VILV2 Pol polyprotein OS=Maedi visna virus (strain 1514 / clone LV1-1KS2) GN=pol PE=3 SV=1
P35956 **Mass:** 126098 **Score:** 26 **Matches:** 1(1) **Sequences:** 1(1)
sp|P35956|POL_VILVK Pol polyprotein OS=Maedi visna virus (strain KV1772) GN=pol PE=1 SV=1

---

17. P0C276 **Mass:** 14719 **Score:** 25 **Matches:** 1(1) **Sequences:** 1(1) **emPAI:** 0.23
sp|P0C276|RL40_SHEEP Ubiquitin-60S ribosomal protein L40 OS=Ovis aries GN=UBA52 PE=2 SV=2
☐ Check to include this hit in error tolerant search or archive report

Query	Observed	Mr(expt)	Mr(calc)	ppm	Miss	Score	Expect	Rank	Unique	Peptide
449	541.2789	1080.5432	1080.5451	-1.81	0	25	0.0038	1	U	R.TLSDYNIQK.E

Proteins matching the same set of peptides:
P0CG55 **Mass:** 34305 **Score:** 25 **Matches:** 1(1) **Sequences:** 1(1)
sp|P0CG55|UBB_SHEEP Polyubiquitin-B OS=Ovis aries GN=UBB PE=2 SV=1

Peptide matches not assigned to protein hits: (no details means no match)

Query	Observed	Mr(expt)	Mr(calc)	ppm	Miss	Score	Expect	Rank	Unique	Peptide
115	416.2304	830.4463	830.4498	-4.18	1	20	0.014	1		SVDPKTGK
683	611.3280	1220.6414	1220.6070	28.2	1	18	0.017	1		SESIMKLEER
755	632.3501	1262.6856	1262.6870	-1.09	0	18	0.017	1		LGLDIEIATYR
137	425.7659	849.5173	849.5184	-1.39	0	17	0.018	1		HLQLAIR
903	680.3560	1358.6974	1358.6500	34.9	1	17	0.076	1		AIEEACKQGPEK + Carbamidomethyl (C)
49	382.7141	763.4136	763.4228	-12.10	0	16	0.022	1		YILEAR
75	394.7417	787.4689	787.4956	-33.93	1	16	0.026	1		KITWLK
151	430.2200	858.4255	858.4276	-2.41	0	16	0.026	1		GFFYTPK
153	430.2206	858.4266	858.4276	-1.13	0	16	0.027	1		GFFYTPK
138	425.7660	849.5175	849.5184	-1.08	0	16	0.027	1		HLQLAIR
754	632.3498	1262.6851	1262.6870	-1.55	0	16	0.029	1		LGLDIEIATYR
74	394.7416	787.4686	787.4956	-34.21	1	16	0.028	1		KITWLK
48	382.7137	763.4129	763.4228	-12.97	0	15	0.031	1		YILEAR
334	505.8144	1009.6142	1009.6172	-2.94	0	14	0.038	1		LIIGPLQEK
411	528.8071	1055.5997	1055.5836	15.3	1	14	0.043	1		LIRQANEGR
901	680.3547	1358.6949	1358.6500	33.1	1	13	0.089	1		AIEEACKQGPEK + Carbamidomethyl (C)
663	400.2393	1197.6959	1197.6982	-1.92	0	13	0.047	1		AVFPSIVGRPR
300	495.2717	988.5288	988.5301	-1.36	0	13	0.072	1		SEITDLRR
498	554.2933	1106.5720	1106.6270	-49.67	1	12	0.16	1		LTFIKNLCR
41	380.2383	758.4620	758.4538	10.8	0	12	0.076	1		ITDIIGK
749	630.8210	2519.2547	2519.2366	7.18	0	12	0.09	1		SFTSQTYMDSSTVHLFVTIISR
1489	680.7080	2039.1022	2039.0180	41.3	1	12	0.062	1		MNTTDCFIAVVNAIKEVR + Oxidation (M)
1519	691.6812	2072.0217	2072.0911	-33.48	1	12	0.09	1		VTGYACLVGDKVMKPAHVK + Carbamidomethyl (C)
140	426.2166	850.4187	850.3855	39.1	0	12	0.086	1		TCSVNEAK
25	367.7305	733.4464	733.4374	12.2	0	12	0.067	1		VLFDLK
684	611.3281	1220.6416	1220.6070	28.4	1	12	0.07	1		SESIMKLEER
40	379.2441	756.4737	756.4857	-15.92	1	11	0.072	1		LLDLRK
45	381.2303	760.4460	760.4119	44.8	0	11	0.073	1		WSVELK
483	551.7962	1101.5778	1101.5666	10.2	0	11	0.11	1		ASAAVSAAPTEK
410	528.8049	1055.5952	1055.5836	11.0	1	11	0.084	1		LIRQANEGR
168	437.2328	872.4511	872.4464	5.34	1	11	0.28	1		NNRTSGPK
609	589.8393	1177.6640	1177.6231	34.8	0	11	0.083	1		LVDTFLEDVK
440	538.8074	1075.6003	1075.6389	-35.96	1	10	0.1	1		EIKAFLLSR
481	550.8193	1099.6240	1099.5848	35.7	0	10	0.1	1		YIAVCHPLK + Carbamidomethyl (C)
23	365.2258	728.4371	728.4181	26.2	0	10	0.13	1		QAEILR
400	524.7747	1047.5348	1047.5019	31.4	1	10	0.14	1		QDVNGKEMK
228	465.2222	928.4297	928.4614	-34.09	0	10	0.11	1		ENDPSVLR
139	426.2166	850.4186	850.3855	39.0	0	10	0.14	1		TCSVNEAK
26	367.7314	733.4482	733.4374	14.6	0	9	0.11	1		VLFDLK
1053	371.6794	1482.6885	1482.7150	-17.89	1	9	0.11	1		RHHTDVGFIMDR
1401	931.9813	3723.8960	3723.8236	19.4	1	9	0.25	1		AMIQSALDGSIIENGNVATRDVDVFIGACSEPIYR
482	551.7961	1101.5777	1101.5666	10.1	0	9	0.17	1		ASAAVSAAPTEK
515	560.3113	1118.6080	1118.6448	-32.85	1	9	0.21	1		LELYKQGLR
398	524.7739	1047.5332	1047.5019	29.9	1	9	0.19	1		QDVNGKEMK
610	589.8421	1177.6696	1177.6231	39.6	0	8	0.15	1		LVDTFLEDVK
214	457.2933	912.5720	912.5644	8.37	0	8	0.16	1		VLLSSPAVK
627	394.8756	1181.6050	1181.6404	-30.02	0	8	0.26	1		VDTVSAPAPGLR
279	485.7542	969.4938	969.5244	-31.53	0	8	0.16	1		VVSDPQGIR
484	551.7963	1101.5780	1101.5666	10.4	0	8	0.24	1		ASAAVSAAPTEK
44	381.2303	760.4460	760.4119	44.8	0	8	0.17	1		WSVELK
38	379.2438	756.4730	756.4857	-16.89	1	7	0.18	1		LLDLRK
86	402.6984	803.3823	803.4025	-25.13	0	7	0.19	1		ATDEQLK
291	492.2796	982.5447	982.5811	-37.06	1	7	0.19	1		LIDPERIK
249	475.7446	949.4747	949.4981	-24.69	1	7	0.21	1		EARSNVFK
83	400.7341	799.4535	799.4341	24.4	0	7	0.2	1		DLIHFR
822	652.3350	1302.6554	1302.6905	-26.96	1	7	0.27	1		RAAPGDGPRPGPR
142	426.2170	850.4195	850.3855	40.0	0	7	0.27	1		TCSVNEAK
493	553.2912	1104.5679	1104.5788	-9.85	1	7	0.2	1		RQYLQQNR
823	652.3355	1302.6565	1302.6905	-26.10	1	7	0.28	1		RAAPGDGPRPGPR
1651	654.0679	2612.2425	2612.2046	14.5	0	7	0.22	1		SEFVLPSYFDQWIYSPMFNAR + Oxidation (M)
46	381.2303	760.4460	760.4119	44.9	0	7	0.22	1		WSVELK
437	538.8056	1075.5967	1075.6389	-39.29	1	7	0.26	1		EIKAFLLSR
171	437.7517	873.4888	873.4709	20.6	0	7	0.22	1		EAGVFVPR
1073	501.9295	1502.7666	1502.8133	-31.08	0	6	0.3	1		GDILQVFDGWILK
441	538.8076	1075.6006	1075.6389	-35.64	1	6	0.23	1		EIKAFLLSR
36	375.6956	749.3766	749.3530	31.4	0	6	0.28	1		IMEWR + Oxidation (M)
1040	490.9009	1469.6808	1469.6894	-5.88	0	6	0.23	1		LQVSQEEFLCMK + Oxidation (M)
413	529.3007	1056.5868	1056.5637	21.9	0	6	0.24	1		LDEIMAPIR
134	423.2409	844.4672	844.4654	2.13	0	6	0.86	1		SIEEIVR
110	414.2182	826.4218	826.4007	25.5	0	6	0.25	1		TGMFSIR + Oxidation (M)
6	351.1994	700.3843	700.3980	-19.54	1	6	0.29	1		ERAVAR
8	353.2168	704.4191	704.3891	42.6	0	6	0.25	1		MLVAAGK + Oxidation (M)

☑	872	448.2413	1341.7020	1341.7365	-25.64	1	6	0.25	1	APTASGSGGAIRGIK
☑	313	497.2571	1488.7494	1488.8049	-37.30	1	6	0.77	1	FVPDGSVASRSVIR
☑	248	475.7446	949.4747	949.4981	-24.69	1	6	0.28	1	EARSNVFK
☑	84	400.7466	799.4786	799.4803	-2.14	0	6	0.27	1	AINELIK
☑	289	491.2498	980.4851	980.4419	44.0	0	6	1	1	SSLPSMMGR + Oxidation (M)
☑	580	386.8820	1157.6241	1157.6193	4.10	1	6	0.28	1	KDPTQAFVPR
☑	1146	524.3021	1569.8846	1569.8151	44.2	0	5	0.29	1	VTLVFEHVDQDLR
☑	345	511.2742	1020.5339	1020.5577	-23.31	1	5	0.3	1	RTQPLNHR
☑	290	492.2796	982.5446	982.5811	-37.10	1	5	0.3	1	LIDPERIK
☑	489	552.7694	1103.5242	1103.5103	12.6	0	5	0.37	1	GMEHLLNMK + 2 Oxidation (M)
☑	82	400.7335	799.4524	799.4341	23.0	0	5	0.31	1	DLIHFR
☑	117	416.2502	830.4858	830.4498	43.4	0	5	0.35	1	LENAGSLK
☑	659	598.8220	1195.6295	1195.5880	34.7	0	5	0.31	1	CGHTNNLRPK + Carbamidomethyl (C)
☑	1672	924.1470	2769.4191	2769.2981	43.7	1	5	0.31	1	AEDGSVIDYELIDQDARDLYDAGVK
☑	392	523.8017	1045.5888	1045.5702	17.8	1	5	0.32	1	LMKLQNQR + Oxidation (M)
☑	156	432.7138	863.4130	863.4501	-42.94	1	5	0.32	1	IKDAFDR
☑	1491	683.0295	2046.0668	2046.0647	1.05	1	5	0.44	1	DRVGGLPAFLENTFIGNAR
☑	415	529.3100	1056.6055	1056.6543	-46.18	1	5	0.33	1	LGIESKGLLK
☑	758	633.3215	1264.6284	1264.6155	10.2	0	5	0.35	1	AGSVGAEACLMLK + Oxidation (M)
☑	236	471.7928	941.5710	941.5658	5.51	1	5	0.34	1	KALLDLNR
☑	235	471.7904	941.5662	941.5294	39.1	0	5	0.35	1	NIVEAAAVR
☑	761	634.3413	1266.6681	1266.6819	-10.91	1	4	0.39	1	QIPPLNLETKD
☑	559	573.2925	1144.5705	1144.5724	-1.61	1	4	0.54	1	EGLQENKEAK
☑	1041	490.9013	1469.6822	1469.6894	-4.94	0	4	0.36	1	LQVSQEEFLCMK + Oxidation (M)
☑	492	553.2888	1104.5630	1104.5788	-14.27	1	4	0.36	1	RQYLQQNR
☑	427	534.2583	1066.5021	1066.5090	-6.49	1	4	0.37	1	GDCPRAVGHR
☑	1150	785.9504	1569.8862	1569.8151	45.3	0	4	0.37	1	VTLVFEHVDQDLR
☑	111	414.2185	826.4225	826.4007	26.4	0	4	0.37	1	TGMFSIR + Oxidation (M)
☑	344	511.2742	1020.5338	1020.5577	-23.42	1	4	0.38	1	RTQPLNHR
☑	35	375.2139	748.4133	748.4119	1.85	0	4	0.57	1	FNEIVK
☑	428	534.3034	2133.1846	2133.1041	37.7	0	4	1.2	1	EIGWMEGGQLVLGNPAPIPR
☑	1027	730.3837	2917.5056	2917.5367	-10.65	1	4	0.75	1	ALSVEFPEMMSEVIAAQLPKILAGMVK + Oxidation (M)
☑	201	453.7375	905.4605	905.4719	-12.57	0	4	0.56	1	NFTAAVQR
☑	362	515.7799	1029.5453	1029.5455	-0.14	0	4	0.42	1	EIQTVVGER
☑	1151	524.3027	1569.8863	1569.8151	45.4	0	4	0.42	1	VTLVFEHVDQDLR
☑	31	374.2280	746.4415	746.4221	26.0	1	4	0.42	1	VVMARR + Oxidation (M)
☑	658	598.8216	1195.6287	1195.6349	-5.24	1	4	0.42	1	GNDSRFLFLK
☑	417	529.3106	1056.6066	1056.6543	-45.08	1	4	0.43	1	LGIESKGLLK
☑	578	578.8386	1155.6627	1155.6248	32.8	1	4	0.43	1	QSPVDVDLKR
☑	218	458.2619	1371.7639	1371.7796	-11.44	1	4	0.84	1	KCIVLIIVGDDK + Carbamidomethyl (C)
☑	285	487.7646	973.5147	973.5379	-23.79	1	3	0.76	1	LLSKTCPR + Carbamidomethyl (C)
☑	32	374.2285	746.4424	746.4221	27.2	1	3	0.47	1	VVMARR + Oxidation (M)
☑	1117	511.9568	1532.8484	1532.8061	27.6	0	3	0.47	1	QPVPFQVDCILFK
☑	343	510.7769	1019.5392	1019.5148	24.0	1	3	0.47	1	FSPEAASRR
☑	647	596.8192	1191.6239	1191.6360	-10.17	1	3	0.47	1	RQQLQTYQK
☑	454	541.8182	1081.6219	1081.6608	-35.91	0	3	0.48	1	VGQILAVLNR
☑	30	373.2437	744.4729	744.4494	31.6	0	3	0.48	1	LLSSIGR
☑	686	408.2186	1221.6341	1221.6210	10.8	1	3	0.49	1	KGSNVMLAVCK + Carbamidomethyl (C); Oxidation (M)
☑	1564	738.0028	2210.9865	2211.0855	-44.77	0	3	0.5	1	YEAQAHGHMKPLSASQVSVR + Oxidation (M)
☑	1152	524.3027	1569.8863	1569.8151	45.4	0	3	0.5	1	VTLVFEHVDQDLR
☑	414	529.3096	1056.6046	1056.6543	-46.98	1	3	0.51	1	LGIESKGLLK
☑	894	452.9118	1355.7136	1355.7198	-4.55	0	3	0.51	1	GFTIGPDAGPGVIR
☑	577	386.2251	1155.6533	1155.6863	-28.53	1	3	0.52	1	ISEAKLTGIPK
☑	1096	505.9263	1514.7570	1514.7399	11.3	0	3	0.52	1	AEMSDAVSPINLPR + Oxidation (M)
☑	39	379.2438	756.4731	756.4857	-16.74	1	3	0.55	1	LLDLRK
☑	1648	654.0678	2612.2420	2612.2046	14.3	0	3	0.55	1	SEFVLPSYFDQWIYSPMFNAR + Oxidation (M)
☑	150	428.7690	855.5235	855.4926	36.1	1	3	0.56	1	LRISPDR
☑	272	483.2557	964.4968	964.5032	-6.58	1	3	0.7	1	GPFRFWR
☑	1353	589.3412	1765.0019	1764.9958	3.42	1	3	0.56	1	GLRQQAVLAIGQELNR
☑	94	405.2227	808.4308	808.4265	5.34	1	3	0.56	1	RMFDIK
☑	541	567.3184	1132.6222	1132.6492	-23.87	0	2	0.57	1	NIFVLDITAK
☑	416	529.3105	1056.6065	1056.6543	-45.22	1	2	0.58	1	LGIESKGLLK
☑	81	400.2239	798.4333	798.4460	-15.91	0	2	0.59	1	RPGASVGR
☑	989	714.3708	2853.4541	2853.4081	16.1	1	2	1.1	1	EFVKMLPGDYPFEIQISGGCELLPR + Oxidation (M)
☑	1147	524.3021	1569.8846	1569.8151	44.2	0	2	0.61	1	VTLVFEHVDQDLR
☑	1352	589.3409	1765.0009	1764.9958	2.85	1	2	0.61	1	GLRQQAVLAIGQELNR
☑	497	554.2760	2213.0748	2213.1804	-47.74	1	2	1.5	1	AGIAHLYGIAGTTNVTGDQVKK
☑	97	408.2462	1628.9555	1628.9111	27.3	1	2	0.72	1	SSSLPHHVRALGQIK
☑	554	572.7952	1143.5759	1143.5594	14.5	0	2	0.63	1	VTEMPSDLPR
☑	695	613.8410	1225.6675	1225.6601	6.02	1	2	0.63	1	EGKLIMGIGHR + Oxidation (M)
☑	1675	564.8604	2819.2656	2819.3371	-25.35	1	2	0.63	1	NLNNLDISKNNFLSMPETCQWPGK + Carbamidomethyl (C)
☑	266	481.2450	960.4754	960.4487	27.8	0	2	0.95	1	DLPQMWR + Oxidation (M)
☑	540	567.3159	1132.6172	1132.6492	-28.26	0	2	0.66	1	NIFVLDITAK
☑	278	485.7534	969.4921	969.5244	-33.22	0	2	0.64	1	VVSDPQGIR
☑	994	362.2019	1444.7786	1444.7562	15.5	0	2	0.64	1	SPELVAEVAIFDR
☑	1134	782.3803	1562.7461	1562.7909	-28.64	1	2	0.65	1	VEQLSNMIVRSCK + Carbamidomethyl (C)
☑	303	495.2915	988.5684	988.5203	48.7	0	2	0.65	1	VAQGHQPPR
☑	374	519.2727	1036.5309	1036.5342	-3.14	0	2	0.65	1	TLGPFYPSR
☑	1354	589.3413	1765.0021	1764.9958	3.53	1	2	0.67	1	GLRQQAVLAIGQELNR
☑	217	458.2434	1828.9445	1828.9651	-11.28	1	2	1.4	1	KPTCAVRNQMETVKPK
☑	155	432.7137	863.4129	863.4501	-43.06	1	2	0.69	1	IKDAFDR
☑	895	452.9124	1355.7152	1355.7198	-3.36	0	2	0.69	1	GFTIGPDAGPGVIR
☑	434	537.8057	1073.5967	1073.6485	-48.17	0	2	1	1	IPAYGVLTIK
☑	10	357.2302	712.4459	712.4119	47.6	0	2	0.7	1	GLIGPEK
☑	527	564.2897	1126.5648	1126.6207	-49.59	1	1	0.76	1	SQNIPTRVGR
☑	253	477.3042	952.5938	952.5705	24.4	0	1	0.75	1	LLEVLNPR
☑	435	537.8057	1073.5968	1073.6485	-48.12	0	1	1.1	1	IPAYGVLTIK
☑	431	537.7659	1073.5173	1073.4699	44.1	0	1	1.6	1	QESEEPPMK
☑	270	482.2320	1443.6741	1443.7180	-30.42	0	1	1.4	1	ITANSVYGLMGFR + Oxidation (M)
☑	1514	690.0050	2066.9930	2067.0282	-17.00	0	1	0.78	1	IAVNCFGQHVTIALMSYK + Carbamidomethyl (C); Oxidation (M)
☑	116	416.2500	830.4855	830.4610	29.6	0	1	0.9	1	SQLSQLR
☑	1116	767.4297	1532.8448	1532.8061	25.3	0	1	0.82	1	QPVPFQVDCILFK
☑	47	382.2186	762.4227	762.4276	-6.35	0	1	2	1	LFENLK
☑	753	632.3315	1262.6483	1262.6507	-1.82	0	1	0.84	1	AEAGTGFLEQLK

1602	786.0410	2355.1011	2355.0844	7.10	0	1	0.8	1	TITPCISSPCAPAAPCTPCVPR + 3 Carbamidomethyl (C)
488	552.7692	1103.5238	1103.5094	13.1	1	1	1	1	GGEGAEAEEKK
529	565.3107	1128.6067	1128.6502	-38.52	1	1	0.81	1	LEARLSALEK
3	350.7332	699.4518	699.4391	18.2	1	1	0.81	1	RIANVK
1195	413.2566	1648.9973	1648.9624	21.2	1	1	0.81	1	KVINALSGIDLTHLR
205	454.7270	907.4394	907.4069	35.8	0	1	1	1	TCSVNEAK + Carbamidomethyl (C)
76	396.2267	1580.8779	1580.8463	20.0	1	1	1.8	1	HPEDRKPYLWIK
806	648.3163	1294.6180	1294.5944	18.2	1	1	0.82	1	KCCQAGMVLGGR + Carbamidomethyl (C); Oxidation (M)
1441	653.0013	1955.9820	1956.0251	-22.02	0	1	0.83	1	LAALCLADNSVALHYGGLR
146	428.7652	855.5159	855.5542	-44.74	1	1	0.83	1	IDVILRK
252	477.3041	952.5936	952.5705	24.2	0	1	0.83	1	LLEVLNPR
1102	760.8853	1519.7560	1519.7705	-9.53	0	1	0.84	1	MVVVVSDHYLQSK + Oxidation (M)
1678	979.4890	2935.4453	2935.4064	13.2	1	1	0.84	1	SLEKVKPGMMCSVAGWGQLGVNMPSADK + Oxidation (M)
220	459.2472	1832.9597	1832.9091	27.6	1	1	3.9	1	VLNSFSNGMKHLDDLK + Oxidation (M)
1647	654.0668	2612.2382	2612.2046	12.9	0	1	0.84	1	SEFVLPSYFDQWIYSPMFNAR + Oxidation (M)
779	643.7832	1285.5518	1285.5543	-1.98	0	1	0.86	1	LHDCSCLSHSK + Carbamidomethyl (C)
1685	804.6562	3214.5955	3214.6982	-31.93	0	0	0.89	1	SMVVADNGELLSEGGVPGIVALFVVLECIIR + Oxidation (M)
1671	924.1459	2769.4160	2769.2981	42.6	1	0	0.92	1	AEDGSVIDYELIDQDARDLYDAGVK
485	552.3132	1102.6118	1102.5805	28.4	0	0	1.2	1	INDMAGITIR
147	428.7654	855.5163	855.5542	-44.30	1	0	0.92	1	IDVILRK
227	465.2215	928.4285	928.4614	-35.42	0	0	0.93	1	ENDPSVLR
1674	697.0620	2784.2187	2784.3325	-40.88	1	0	0.93	1	SFAGIRFGCFMPLSLDSDLTMYLK + Carbamidomethyl (C); Oxidation (M)
534	566.3138	1130.6131	1130.5680	39.9	0	0	0.95	1	LNGNATIESGR
1673	924.1471	2769.4194	2769.2981	43.8	1	0	0.95	1	AEDGSVIDYELIDQDARDLYDAGVK
896	452.9124	1355.7155	1355.7198	-3.13	0	0	0.96	1	GFTIGPDAGPGVIR
1484	507.9833	2027.9040	2027.9808	-37.89	1	0	0.96	1	FACQTFLKDEIRPMEK + Carbamidomethyl (C); Oxidation (M)
251	477.3040	952.5934	952.5705	24.0	0	0	0.97	1	LLEVLNPR
1636	845.7285	2534.1636	2534.1320	12.5	0	0	0.97	1	VMPISGDYVQGFMSYMVPYYR + 2 Oxidation (M)
1252	562.2678	1683.7815	1683.8324	-30.25	1	0	0.98	1	LGNVYLNIKMSCEK + Carbamidomethyl (C); Oxidation (M)
185	448.7394	895.4642	895.4399	27.1	0	0	0.98	1	IETNYTR
34	375.2126	748.4107	748.4344	-31.61	1	0	3.2	1	RISAFR
1	350.0632	698.1118							
2	350.7323	699.4500							
4	350.7333	699.4519							
5	350.7333	699.4520							
7	353.2089	704.4032							
9	357.1881	712.3616							
11	357.2303	712.4460							
12	357.2304	712.4462							
13	359.3136	716.6126							
14	359.3145	716.6144							
15	359.3150	716.6155							
16	362.0946	722.1746							
17	362.0953	722.1761							
18	362.1170	722.2194							
19	363.0920	724.1695							
20	364.2109	726.4072							
21	364.2254	726.4362							
22	364.2254	726.4362							
24	365.2277	728.4409							
27	369.2125	736.4104							
28	371.6801	741.3456							
29	371.7315	741.4485							
33	374.7167	747.4188							
37	376.2073	750.4001							
42	380.7908	759.5670							
43	381.2301	760.4457							
50	383.8720	765.7295							
51	384.2348	766.4550							
52	385.7413	769.4681							
53	387.7152	773.4159							
55	390.6997	779.3849							
56	390.8137	779.6129							
57	391.2817	780.5489							
58	391.2819	780.5492							
59	391.2819	780.5493							
60	391.2821	780.5496							
61	391.2822	780.5498							
62	391.2823	780.5501							
63	391.2824	780.5502							
64	391.2825	780.5504							
65	391.2825	780.5505							
66	391.2826	780.5506							
67	391.2828	780.5510							
68	391.2833	780.5521							
69	391.2833	780.5521							
70	391.2834	780.5523							
71	391.2835	780.5525							
72	391.2837	780.5529							
73	391.2847	780.5549							
77	397.9761	793.9376							
85	402.0591	802.1036							
87	403.2314	804.4482							
88	403.2314	804.4483							
89	403.2314	804.4483							
93	405.2227	808.4308							
95	405.2251	808.4355							
96	406.2489	810.4832							
98	408.7389	815.4633							
99	409.7407	817.4668							
100	409.7413	817.4681							
103	412.2394	822.4643							
104	412.7520	823.4895							
105	412.7521	823.4896							

☑	106	412.7524	823.4902
☑	107	413.2444	824.4741
☑	108	414.2059	826.3972
☑	109	414.2182	826.4218
☑	112	414.8723	827.7299
☑	113	414.8739	827.7332
☑	114	415.7393	829.4639
☑	118	416.2520	830.4894
☑	119	416.7507	831.4868
☑	120	416.7519	831.4893
☑	121	418.2195	834.4244
☑	122	418.2208	834.4270
☑	123	419.7577	837.5009
☑	124	420.2284	838.4423
☑	125	421.7575	841.5005
☑	126	421.7576	841.5006
☑	127	421.7577	841.5008
☑	128	421.7578	841.5010
☑	129	421.7580	841.5014
☑	130	421.7581	841.5016
☑	131	421.7581	841.5016
☑	132	422.7603	843.5061
☑	133	423.2382	844.4619
☑	135	424.7336	847.4526
☑	136	424.7340	847.4534
☑	141	426.2168	850.4191
☑	143	427.2252	852.4358
☑	144	428.2616	854.5086
☑	145	428.7593	855.5040
☑	148	428.7655	855.5164
☑	149	428.7656	855.5166
☑	157	433.2240	864.4334
☑	158	433.2240	864.4334
☑	159	433.2243	864.4340
☑	160	434.7117	867.4089
☑	162	435.7704	869.5262
☑	163	435.7716	869.5286
☑	164	435.7732	869.5318
☑	165	435.7736	869.5327
☑	166	435.7737	869.5328
☑	167	435.7737	869.5328
☑	169	437.2561	872.4977
☑	170	437.7514	873.4882
☑	172	438.7637	875.5128
☑	173	441.2729	880.5312
☑	174	441.7542	881.4938
☑	175	445.7499	889.4852
☑	176	445.7514	889.4882
☑	177	445.7603	889.5061
☑	178	446.2155	890.4164
☑	179	446.7521	891.4897
☑	180	447.7187	893.4228
☑	181	447.7190	893.4234
☑	182	448.2126	894.4106
☑	183	448.2131	894.4116
☑	184	448.7393	895.4640
☑	186	449.2391	896.4636
☑	187	449.2443	896.4740
☑	188	449.2445	896.4745
☑	189	449.7572	897.4998
☑	190	450.2683	898.5220
☑	191	450.2685	898.5225
☑	192	450.2984	898.5823
☑	193	450.2985	898.5825
☑	194	450.2985	898.5825
☑	195	451.2523	900.4900
☑	196	451.2545	900.4944
☑	198	452.2605	902.5065
☑	199	453.2453	904.4761
☑	202	453.7835	905.5524
☑	206	454.7497	907.4849
☑	209	457.2076	912.4006
☑	210	457.2583	912.5020
☑	211	457.2588	912.5031
☑	212	457.2763	912.5381
☑	213	457.2764	912.5383
☑	215	457.7859	913.5573
☑	216	457.9154	913.8163
☑	219	459.2400	916.4654
☑	221	459.2786	916.5426
☑	222	459.9111	917.8077
☑	223	460.7568	919.4990
☑	224	460.7569	919.4992
☑	225	461.7344	921.4543
☑	226	461.7345	921.4545
☑	229	465.2851	928.5557
☑	230	465.2859	928.5572
☑	231	468.2440	934.4734
☑	232	469.7606	937.5065
☑	233	471.7846	941.5547
☑	234	471.7863	941.5580
☑	237	471.7930	941.5714
☑	238	472.2583	942.5021
☑	239	472.2584	942.5022

240	472.2598	942.5051
241	472.2873	942.5600
245	473.2787	944.5429
246	474.1328	946.2510
247	475.2585	948.5024
250	476.2321	950.4497
254	478.8087	955.6028
255	478.8101	955.6056
256	479.2865	956.5584
257	479.2887	956.5628
258	479.2887	956.5629
259	479.2890	956.5634
260	479.2891	956.5637
261	479.2892	956.5639
262	479.2894	956.5642
263	479.2895	956.5645
264	479.2899	956.5653
265	479.7647	957.5149
267	481.2452	960.4757
268	481.2453	960.4760
269	482.2298	962.4450
271	482.2476	962.4806
273	483.2558	964.4971
274	483.2564	964.4981
275	483.7832	965.5519
276	483.7860	965.5575
277	484.7509	967.4873
280	486.2976	970.5806
281	487.2744	972.5342
282	487.2745	972.5345
283	487.2746	972.5346
284	487.2746	972.5346
286	488.2783	974.5420
287	488.2795	974.5444
288	489.2534	976.4922
296	493.2855	984.5565
297	493.7623	985.5101
298	494.3008	986.5871
299	495.2681	988.5216
301	495.2835	988.5525
302	495.2836	988.5526
304	495.2915	988.5684
305	495.2915	988.5685
306	495.2916	988.5687
307	497.2517	992.4888
308	497.2523	992.4899
309	497.2536	992.4927
310	497.2538	992.4929
311	497.2539	992.4932
312	497.2544	992.4943
314	498.2167	994.4188
315	499.2746	996.5347
316	499.2756	996.5367
317	499.7625	997.5104
318	499.8002	997.5858
319	499.8012	997.5878
320	499.8034	997.5923
321	499.8042	997.5938
322	499.8043	997.5941
323	500.7522	999.4898
324	500.7524	999.4902
325	500.7684	999.5222
326	500.7717	999.5288
327	500.7748	999.5351
328	500.7753	999.5360
329	501.7626	1001.5106
330	501.9303	1001.8461
331	505.2434	1008.4723
332	505.2690	1008.5234
333	505.2698	1008.5250
335	507.2659	1012.5173
336	507.3014	1012.5882
337	507.3024	1012.5903
338	507.7992	1013.5838
339	507.7994	1013.5842
340	508.7919	1015.5692
341	510.2603	1018.5061
342	510.2604	1018.5061
346	511.7664	1021.5183
347	511.7666	1021.5186
348	512.7740	1023.5333
349	512.7740	1023.5335
350	513.2840	1024.5535
351	513.2840	1024.5535
352	513.3082	1024.6018
353	513.3084	1024.6022
354	513.3085	1024.6024
355	513.3086	1024.6026
356	513.7640	1025.5135
357	515.2999	1028.5853
358	515.2999	1028.5853
359	515.7402	1029.4659
360	515.7406	1029.4666
361	515.7421	1029.4696

☑	363	515.7800	1029.5455
☑	364	515.7835	1029.5524
☑	368	516.3125	1030.6105
☑	369	517.7923	1033.5700
☑	370	518.1306	1034.2466
☑	371	518.7733	1035.5321
☑	372	518.7735	1035.5325
☑	373	519.2709	1036.5273
☑	375	519.7671	1037.5196
☑	376	520.2990	1038.5835
☑	377	520.2996	1038.5846
☑	378	520.8262	1039.6378
☑	379	520.8264	1039.6383
☑	380	521.7246	1041.4347
☑	381	521.7970	1041.5794
☑	382	521.7972	1041.5798
☑	383	521.7972	1041.5799
☑	384	521.7973	1041.5800
☑	386	522.2735	1042.5325
☑	388	522.2936	1042.5726
☑	389	523.2669	1044.5193
☑	390	523.7711	1045.5276
☑	391	523.7729	1045.5312
☑	393	523.8018	1045.5891
☑	394	523.8018	1045.5891
☑	395	523.8030	1045.5915
☑	396	524.7614	1047.5083
☑	397	524.7615	1047.5084
☑	402	525.7646	1049.5147
☑	403	525.7650	1049.5153
☑	404	525.7688	1049.5230
☑	405	526.2766	1050.5386
☑	406	527.2887	1052.5629
☑	407	527.2894	1052.5642
☑	408	528.7759	1055.5373
☑	409	528.7762	1055.5378
☑	412	529.2948	1056.5751
☑	418	530.2922	1058.5698
☑	419	530.2939	1058.5732
☑	420	530.7845	1059.5545
☑	421	530.7861	1059.5577
☑	424	533.2619	1064.5092
☑	425	533.7855	1065.5565
☑	426	534.2541	1066.4937
☑	429	536.2560	1070.4975
☑	430	536.7556	1071.4967
☑	432	537.8035	1073.5925
☑	433	537.8055	1073.5965
☑	436	538.3016	1074.5886
☑	438	538.8064	1075.5982
☑	439	538.8064	1075.5982
☑	442	539.2828	1076.5510
☑	443	539.7414	1077.4682
☑	444	539.7633	1077.5121
☑	445	539.7636	1077.5126
☑	446	540.7982	1079.5818
☑	447	540.9097	1079.8048
☑	448	541.2760	1080.5374
☑	450	541.7823	1081.5500
☑	451	541.7824	1081.5503
☑	452	361.5378	1081.5917
☑	453	541.8181	1081.6217
☑	455	542.1968	1082.3791
☑	456	542.3062	1082.5978
☑	457	542.7837	1083.5528
☑	458	543.3131	1084.6116
☑	459	543.3134	1084.6122
☑	460	543.3151	1084.6156
☑	461	543.7651	1085.5156
☑	462	543.7652	1085.5158
☑	463	543.7658	1085.5170
☑	464	544.2509	1086.4872
☑	465	544.2658	1086.5171
☑	466	544.2848	1086.5551
☑	467	364.5694	1090.6863
☑	468	546.6289	1091.2433
☑	469	364.8645	1091.5717
☑	470	546.8354	1091.6562
☑	471	547.2668	1092.5191
☑	472	547.3294	1092.6443
☑	473	547.8140	1093.6134
☑	474	550.3041	1098.5936
☑	475	550.3045	1098.5944
☑	480	550.8047	1099.5949
☑	486	368.5530	1102.6370
☑	487	368.5532	1102.6378
☑	490	552.7695	1103.5245
☑	491	553.2820	1104.5494
☑	494	369.2043	1104.5910
☑	495	553.7903	1105.5661
☑	496	554.2754	1106.5362
☑	499	554.3039	1106.5933
☑	500	554.3039	1106.5933
☑	501	554.7500	1107.4854

☑	502	554.7500	1107.4854
☑	503	370.8469	1109.5189
☑	504	556.9493	1111.8839
☑	505	557.8202	1113.6259
☑	506	557.8207	1113.6267
☑	508	558.2997	1114.5849
☑	509	558.3016	1114.5886
☑	510	558.7740	1115.5334
☑	511	559.7948	1117.5751
☑	512	559.8187	1117.6227
☑	513	559.8188	1117.6229
☑	514	560.2967	1118.5788
☑	516	374.2048	1119.5925
☑	517	374.2048	1119.5925
☑	518	561.2952	1120.5757
☑	519	561.2954	1120.5762
☑	520	561.2954	1120.5762
☑	521	561.7921	1121.5696
☑	522	561.7922	1121.5698
☑	523	561.8280	1121.6414
☑	524	561.8287	1121.6429
☑	525	561.8288	1121.6430
☑	528	565.3091	1128.6037
☑	530	565.7779	1129.5412
☑	531	565.7779	1129.5413
☑	532	565.7780	1129.5415
☑	533	565.8245	1129.6345
☑	539	566.9799	1131.9452
☑	542	568.3052	1134.5957
☑	543	568.7705	1135.5265
☑	544	568.7707	1135.5268
☑	545	569.2774	1136.5403
☑	546	569.3023	1136.5900
☑	547	569.7723	1137.5301
☑	548	570.3468	1138.6791
☑	549	571.3070	1140.5994
☑	550	571.3074	1140.6002
☑	551	571.3074	1140.6002
☑	552	572.7731	1143.5316
☑	553	572.7740	1143.5335
☑	555	572.7954	1143.5762
☑	556	572.7975	1143.5804
☑	557	572.8376	1143.6606
☑	558	572.8390	1143.6635
☑	560	573.2926	1144.5706
☑	561	573.3202	1144.6259
☑	562	573.3202	1144.6259
☑	563	573.3206	1144.6266
☑	564	574.6686	1147.3226
☑	565	575.3025	1148.5905
☑	566	575.3035	1148.5924
☑	567	383.8717	1148.5932
☑	568	383.8717	1148.5932
☑	569	383.8717	1148.5932
☑	570	383.8717	1148.5932
☑	571	383.8717	1148.5933
☑	572	576.2868	1150.5591
☑	573	385.2139	1152.6198
☑	574	385.2139	1152.6198
☑	575	577.7706	1153.5266
☑	576	578.3289	1154.6433
☑	579	578.8390	1155.6634
☑	581	581.2796	1160.5446
☑	583	581.3137	1160.6128
☑	584	581.3148	1160.6150
☑	585	581.3159	1160.6172
☑	602	586.3187	1170.6228
☑	603	586.3187	1170.6229
☑	604	586.3191	1170.6237
☑	605	586.8431	1171.6716
☑	606	587.8255	1173.6364
☑	607	588.8044	1175.5942
☑	608	588.8044	1175.5943
☑	611	590.3036	1178.5926
☑	612	590.3038	1178.5930
☑	613	590.3150	1178.6154
☑	614	590.3152	1178.6158
☑	615	590.3224	1178.6303
☑	616	590.3229	1178.6313
☑	617	590.3240	1178.6335
☑	618	590.3260	1178.6374
☑	619	590.3263	1178.6381
☑	620	590.3274	1178.6402
☑	621	590.8121	1179.6096
☑	622	590.8137	1179.6128
☑	623	590.8138	1179.6130
☑	624	590.8149	1179.6152
☑	625	590.8150	1179.6154
☑	626	590.8250	1179.6355
☑	628	395.8651	1184.5736
☑	629	395.8654	1184.5744
☑	630	395.8656	1184.5749
☑	631	395.8656	1184.5750
☑	632	593.3089	1184.6033

☑	633	593.3090	1184.6034
☑	634	593.3091	1184.6037
☑	635	593.3092	1184.6038
☑	636	593.3092	1184.6039
☑	637	395.9031	1184.6873
☑	638	593.3513	1184.6880
☑	639	593.7962	1185.5779
☑	640	593.8279	1185.6413
☑	641	593.8304	1185.6463
☑	642	396.2237	1185.6494
☑	643	396.2237	1185.6494
☑	644	593.8327	1185.6509
☑	645	595.2768	1188.5390
☑	646	595.2787	1188.5429
☑	648	596.8193	1191.6240
☑	649	596.8198	1191.6251
☑	650	597.2835	1192.5525
☑	651	597.2837	1192.5528
☑	652	597.3057	1192.5969
☑	653	597.3077	1192.6008
☑	654	597.8274	1193.6402
☑	655	399.2147	1194.6221
☑	656	598.8168	1195.6190
☑	657	598.8177	1195.6209
☑	660	599.3498	1196.6850
☑	661	599.8175	1197.6204
☑	664	600.8384	1199.6623
☑	665	601.2952	1200.5758
☑	666	601.2960	1200.5774
☑	667	601.2967	1200.5789
☑	668	601.8288	1201.6431
☑	669	601.9965	1201.9785
☑	670	603.0118	1204.0090
☑	671	603.3205	1204.6265
☑	672	604.3016	1206.5887
☑	673	604.3185	1206.6225
☑	674	604.8123	1207.6100
☑	675	405.8986	1214.6740
☑	676	608.8555	1215.6964
☑	677	608.8558	1215.6971
☑	678	608.8560	1215.6974
☑	679	608.8561	1215.6977
☑	680	609.3335	1216.6525
☑	681	610.3369	1218.6592
☑	682	611.3103	1220.6061
☑	685	408.2140	1221.6201
☑	687	408.2186	1221.6341
☑	688	613.3078	1224.6011
☑	689	613.3089	1224.6031
☑	690	613.3090	1224.6034
☑	691	409.5471	1225.6195
☑	692	409.5475	1225.6207
☑	693	409.5475	1225.6207
☑	694	613.8395	1225.6644
☑	696	613.8709	1225.7273
☑	697	614.2858	1226.5570
☑	698	614.2865	1226.5584
☑	699	614.7891	1227.5636
☑	700	615.3033	1228.5920
☑	701	615.3655	1228.7164
☑	702	615.3661	1228.7176
☑	703	615.3665	1228.7184
☑	704	615.3667	1228.7188
☑	705	615.8177	1229.6208
☑	706	616.8013	1231.5881
☑	707	616.9599	1231.9052
☑	708	617.3202	1232.6258
☑	709	617.3205	1232.6264
☑	710	412.2308	1233.6707
☑	711	618.2900	1234.5654
☑	712	618.2903	1234.5661
☑	713	618.2906	1234.5666
☑	714	619.0040	1235.9934
☑	715	619.3225	1236.6305
☑	716	619.8001	1237.5857
☑	717	619.8004	1237.5862
☑	718	414.2176	1239.6310
☑	719	414.5538	1240.6396
☑	720	621.6551	1241.2957
☑	721	621.8194	1241.6242
☑	722	621.8525	1241.6904
☑	723	621.8573	1241.6999
☑	724	622.2910	1242.5674
☑	725	622.3440	1242.6734
☑	726	415.2447	1242.7122
☑	727	415.8521	1244.5344
☑	728	416.1778	1245.5116
☑	729	416.1808	1245.5206
☑	730	623.8244	1245.6343
☑	731	623.8250	1245.6354
☑	732	623.8253	1245.6361
☑	733	623.8256	1245.6367
☑	734	625.3299	1248.6452
☑	735	625.3301	1248.6456

☑	736	625.8156	1249.6166
☑	737	625.8183	1249.6221
☑	738	625.8223	1249.6300
☑	739	627.3206	1252.6266
☑	740	627.8064	1253.5982
☑	741	628.3057	1254.5969
☑	742	628.3058	1254.5970
☑	743	628.3122	1254.6099
☑	744	628.3354	1254.6562
☑	745	628.7984	1255.5822
☑	746	629.7981	1257.5816
☑	747	629.8007	1257.5868
☑	748	630.3149	1258.6152
☑	750	630.8250	1259.6354
☑	751	631.2976	1260.5806
☑	752	631.2981	1260.5816
☑	757	632.8263	1263.6381
☑	759	422.8923	1265.6550
☑	760	634.3411	1266.6677
☑	762	423.2382	1266.6926
☑	763	634.3537	1266.6928
☑	764	423.2389	1266.6947
☑	765	635.3631	1268.7116
☑	766	635.3633	1268.7121
☑	767	635.7846	1269.5546
☑	768	635.7856	1269.5566
☑	769	636.3148	1270.6151
☑	770	637.3663	1272.7180
☑	771	637.3666	1272.7186
☑	772	638.3250	1274.6355
☑	773	638.8146	1275.6146
☑	774	639.3586	1276.7026
☑	775	639.3588	1276.7031
☑	776	427.2288	1278.6645
☑	777	428.9215	1283.7427
☑	778	643.3430	1284.6715
☑	780	643.8763	1285.7380
☑	781	643.8765	1285.7385
☑	782	643.8766	1285.7387
☑	783	643.8769	1285.7392
☑	784	643.8771	1285.7396
☑	785	643.8774	1285.7403
☑	786	644.3194	1286.6242
☑	787	644.3196	1286.6246
☑	788	644.3199	1286.6252
☑	789	645.2976	1288.5807
☑	790	645.2986	1288.5826
☑	791	645.2987	1288.5829
☑	792	430.5661	1288.6766
☑	793	430.5664	1288.6775
☑	794	431.1734	1290.4983
☑	795	646.7992	1291.5839
☑	796	646.8396	1291.6646
☑	801	431.9072	1292.6999
☑	802	431.9073	1292.7000
☑	803	431.9224	1292.7453
☑	804	431.9224	1292.7454
☑	805	647.8684	1293.7223
☑	807	648.8074	1295.6003
☑	808	648.8078	1295.6010
☑	809	648.8078	1295.6011
☑	810	648.8171	1295.6196
☑	811	649.3137	1296.6129
☑	812	433.5931	1297.7574
☑	813	650.2738	1298.5331
☑	814	433.8559	1298.5458
☑	815	434.1910	1299.5513
☑	818	651.8601	1301.7055
☑	819	651.8605	1301.7065
☑	820	651.8608	1301.7070
☑	821	651.8611	1301.7077
☑	824	436.8948	1307.6627
☑	825	655.3287	1308.6427
☑	826	656.8696	1311.7247
☑	827	656.8698	1311.7251
☑	830	657.8155	1313.6165
☑	831	657.8156	1313.6166
☑	832	657.8162	1313.6179
☑	833	657.8655	1313.7165
☑	834	657.8697	1313.7249
☑	835	657.8697	1313.7249
☑	836	438.9179	1313.7319
☑	837	657.8751	1313.7357
☑	838	438.9266	1313.7578
☑	839	438.9269	1313.7589
☑	840	658.8219	1315.6291
☑	841	439.8973	1316.6701
☑	842	661.8558	1321.6970
☑	843	441.5859	1321.7358
☑	844	441.9081	1322.7025
☑	845	442.5892	1324.7458
☑	846	442.5893	1324.7460
☑	847	664.3302	1326.6458
☑	848	664.3304	1326.6463

☑	849	664.8259	1327.6372
☑	850	664.8261	1327.6376
☑	851	664.8319	1327.6493
☑	852	443.5583	1327.6532
☑	853	665.3321	1328.6496
☑	854	443.9061	1328.6965
☑	855	443.9094	1328.7064
☑	856	665.3654	1328.7162
☑	857	665.3661	1328.7177
☑	858	665.3666	1328.7186
☑	859	665.3672	1328.7198
☑	860	665.8644	1329.7142
☑	861	444.2731	1329.7976
☑	862	666.8296	1331.6447
☑	863	666.8314	1331.6482
☑	864	666.8315	1331.6485
☑	865	666.8520	1331.6895
☑	867	447.5609	1339.6608
☑	869	448.1714	1341.4923
☑	870	671.8444	1341.6742
☑	871	671.8530	1341.6914
☑	873	672.8251	1343.6357
☑	874	672.8256	1343.6367
☑	875	672.8260	1343.6374
☑	876	672.8279	1343.6412
☑	877	448.9244	1343.7513
☑	878	450.5105	1348.5097
☑	879	675.7061	1349.3976
☑	880	676.3482	1350.6818
☑	881	676.3487	1350.6829
☑	882	676.3495	1350.6845
☑	883	676.3814	1350.7483
☑	884	676.3814	1350.7483
☑	885	676.3821	1350.7496
☑	886	676.3828	1350.7510
☑	887	451.2578	1350.7516
☑	888	451.2579	1350.7518
☑	889	677.3484	1352.6823
☑	890	677.3553	1352.6960
☑	891	677.8693	1353.7240
☑	892	677.8700	1353.7254
☑	893	452.5470	1354.6191
☑	897	679.7985	1357.5824
☑	898	679.7985	1357.5824
☑	899	679.7996	1357.5846
☑	900	679.8540	1357.6934
☑	904	681.3465	1360.6784
☑	905	681.3490	1360.6835
☑	906	681.3500	1360.6854
☑	907	681.3501	1360.6856
☑	908	455.8887	1364.6442
☑	909	455.8887	1364.6443
☑	910	685.3563	1368.6980
☑	912	686.3823	1370.7500
☑	913	686.3825	1370.7504
☑	914	686.3827	1370.7508
☑	915	688.3691	1374.7236
☑	916	688.3695	1374.7244
☑	917	688.8354	1375.6563
☑	918	689.3314	1376.6482
☑	919	690.3661	1378.7176
☑	920	690.3661	1378.7176
☑	921	690.3665	1378.7184
☑	922	690.3665	1378.7184
☑	923	690.3665	1378.7184
☑	924	690.3665	1378.7184
☑	925	690.3666	1378.7186
☑	926	690.3666	1378.7186
☑	927	690.3666	1378.7186
☑	928	690.3666	1378.7186
☑	929	690.3666	1378.7186
☑	930	690.3666	1378.7187
☑	931	690.3666	1378.7187
☑	932	460.5808	1378.7205
☑	933	460.5810	1378.7213
☑	934	690.3696	1378.7247
☑	935	691.3275	1380.6404
☑	936	461.5505	1381.6296
☑	937	692.3472	1382.6798
☑	938	692.3473	1382.6800
☑	939	692.3569	1382.6992
☑	940	692.3585	1382.7024
☑	941	692.3585	1382.7024
☑	942	692.3597	1382.7048
☑	943	462.5263	1384.5570
☑	944	693.3664	1384.7181
☑	945	693.8692	1385.7239
☑	946	695.3406	1388.6667
☑	947	695.3412	1388.6678
☑	948	463.9074	1388.7003
☑	949	464.5884	1390.7434
☑	950	464.5888	1390.7446
☑	951	464.5888	1390.7447
☑	952	464.9157	1391.7253

☑	953	465.2490	1392.7251
☑	954	465.2493	1392.7259
☑	955	699.8867	1397.7588
☑	956	700.3799	1398.7452
☑	957	467.2569	1398.7489
☑	958	700.8884	1399.7622
☑	959	700.8902	1399.7659
☑	960	700.8905	1399.7664
☑	961	468.2210	1401.6413
☑	962	701.8398	1401.6650
☑	963	702.3428	1402.6710
☑	964	702.3451	1402.6755
☑	965	468.9136	1403.7189
☑	966	468.9137	1403.7194
☑	967	703.3689	1404.7232
☑	968	703.3697	1404.7249
☑	969	703.3698	1404.7251
☑	970	703.3699	1404.7252
☑	971	703.3701	1404.7256
☑	972	703.3702	1404.7258
☑	973	703.3714	1404.7282
☑	974	703.3809	1404.7472
☑	975	704.0158	1406.0170
☑	976	469.8857	1406.6354
☑	977	470.2648	1407.7726
☑	978	705.3759	1408.7373
☑	979	471.2752	1410.8038
☑	980	471.5427	1411.6064
☑	981	707.8777	1413.7407
☑	982	472.5870	1414.7393
☑	983	472.5872	1414.7397
☑	984	708.8650	1415.7155
☑	985	709.8612	1417.7079
☑	986	714.3664	1426.7183
☑	987	714.3680	1426.7215
☑	988	714.3684	1426.7223
☑	990	478.2171	1431.6294
☑	991	718.3442	1434.6739
☑	992	718.3444	1434.6742
☑	993	718.3444	1434.6742
☑	995	483.2580	1446.7521
☑	996	724.8536	1447.6927
☑	997	724.8543	1447.6940
☑	998	725.9333	1449.8521
☑	999	725.9345	1449.8545
☑	1000	725.9348	1449.8551
☑	1001	725.9349	1449.8553
☑	1002	484.2924	1449.8554
☑	1003	484.2924	1449.8554
☑	1004	484.2924	1449.8554
☑	1005	484.2924	1449.8554
☑	1006	484.2924	1449.8554
☑	1007	484.2924	1449.8554
☑	1008	484.2924	1449.8554
☑	1009	484.2924	1449.8554
☑	1010	484.2924	1449.8555
☑	1011	484.2924	1449.8555
☑	1012	485.9244	1454.7514
☑	1013	728.7201	1455.4257
☑	1014	728.7205	1455.4263
☑	1015	486.2384	1455.6933
☑	1016	486.2591	1455.7556
☑	1017	486.2593	1455.7560
☑	1018	486.2605	1455.7596
☑	1019	728.8908	1455.7671
☑	1020	486.2644	1455.7713
☑	1021	486.2646	1455.7720
☑	1022	486.2648	1455.7727
☑	1023	486.2648	1455.7727
☑	1024	486.2648	1455.7727
☑	1025	486.2650	1455.7730
☑	1026	730.3485	1458.6824
☑	1028	487.2598	1458.7575
☑	1029	487.2602	1458.7586
☑	1030	731.9116	1461.8086
☑	1031	731.9116	1461.8086
☑	1032	732.3617	1462.7088
☑	1033	733.8468	1465.6791
☑	1034	733.8469	1465.6792
☑	1035	489.5998	1465.7776
☑	1036	489.5998	1465.7776
☑	1037	489.5998	1465.7776
☑	1038	733.8961	1465.7777
☑	1039	490.9004	1469.6795
☑	1042	735.8868	1469.7591
☑	1043	490.9317	1469.7733
☑	1044	737.3335	1472.6524
☑	1045	737.3395	1472.6645
☑	1046	738.3756	1474.7366
☑	1047	738.3763	1474.7380
☑	1048	738.3961	1474.7777
☑	1049	738.3962	1474.7779
☑	1050	740.1136	1478.2126
☑	1051	493.9549	1478.8428

1052	494.2181	1479.6326
1054	742.8773	1483.7399
1055	745.3297	1488.6448
1056	745.3297	1488.6449
1057	745.3306	1488.6466
1058	745.3361	1488.6575
1059	745.9905	1489.9665
1060	497.9390	1490.7951
1061	498.5831	1492.7273
1062	749.3899	1496.7651
1063	499.9292	1496.7657
1064	499.9292	1496.7657
1065	749.3911	1496.7676
1066	750.3447	1498.6748
1067	750.3611	1498.7076
1068	750.3611	1498.7076
1069	750.9195	1499.8244
1070	750.9265	1499.8384
1071	501.2088	1500.6046
1072	751.8615	1501.7084
1074	502.8914	1505.6524
1075	502.8916	1505.6531
1076	754.3695	1506.7245
1077	754.4437	1506.8729
1078	754.4456	1506.8767
1079	755.8480	1509.6815
1080	756.3638	1510.7131
1081	756.3646	1510.7146
1082	505.2548	1512.7425
1083	505.2548	1512.7427
1084	505.2548	1512.7427
1085	505.2548	1512.7427
1086	757.3800	1512.7454
1087	757.3809	1512.7472
1088	757.3809	1512.7473
1089	505.2674	1512.7804
1090	757.3981	1512.7816
1091	505.2706	1512.7901
1092	505.5828	1513.7265
1093	505.5868	1513.7385
1094	757.8939	1513.7733
1095	505.6070	1513.7991
1099	506.2595	1515.7568
1100	506.2598	1515.7574
1101	506.6235	1516.8486
1103	507.6040	1519.7900
1104	508.2227	1521.6462
1105	763.8881	1525.7617
1106	764.3598	1526.7051
1107	764.3601	1526.7057
1108	764.3603	1526.7060
1109	764.3614	1526.7083
1110	764.3669	1526.7192
1111	764.8731	1527.7316
1112	764.8734	1527.7323
1113	510.9139	1529.7200
1114	510.9140	1529.7203
1115	510.9146	1529.7220
1118	511.9568	1532.8487
1119	513.9251	1538.7534
1120	513.9414	1538.8024
1125	770.9781	1539.9417
1126	514.6143	1540.8210
1127	514.6145	1540.8218
1128	514.8601	1541.5586
1129	781.9091	1561.8036
1130	781.9094	1561.8043
1131	781.9147	1561.8148
1132	521.8993	1562.6760
1133	521.8993	1562.6761
1135	783.4472	1564.8798
1136	783.4490	1564.8834
1137	522.6352	1564.8838
1138	783.4501	1564.8857
1139	522.8930	1565.6573
1140	522.8930	1565.6573
1141	784.3756	1566.7367
1142	784.3779	1566.7412
1143	784.5960	1567.1774
1144	785.8916	1569.7687
1145	785.9491	1569.8837
1148	524.3021	1569.8846
1149	524.3021	1569.8846
1153	524.3027	1569.8863
1154	785.9505	1569.8864
1155	787.8379	1573.6613
1156	788.8458	1575.6771
1157	790.0633	1578.1120
1158	527.5458	1579.6155
1159	790.8859	1579.7571
1160	790.8860	1579.7575
1161	527.6186	1579.8340
1162	527.6189	1579.8349
1163	527.6191	1579.8356

☑	1164	793.8809	1585.7472
☑	1165	793.8810	1585.7474
☑	1166	793.8815	1585.7485
☑	1169	530.2987	1587.8743
☑	1170	530.9624	1589.8653
☑	1171	531.9464	1592.8174
☑	1172	797.4249	1592.8353
☑	1173	797.4267	1592.8389
☑	1174	800.3706	1598.7266
☑	1175	534.2874	1599.8403
☑	1176	801.0211	1600.0277
☑	1177	804.3844	1606.7543
☑	1178	804.9059	1607.7973
☑	1179	808.1621	1614.3096
☑	1180	539.9072	1616.6999
☑	1181	540.2728	1617.7965
☑	1182	540.2728	1617.7965
☑	1183	541.9403	1622.7992
☑	1184	407.7108	1626.8142
☑	1185	544.2938	1629.8594
☑	1186	818.1527	1634.2908
☑	1187	546.6246	1636.8520
☑	1188	547.2249	1638.6530
☑	1189	821.2204	1640.4263
☑	1190	547.9462	1640.8167
☑	1191	550.2798	1647.8176
☑	1192	824.9179	1647.8213
☑	1193	824.9179	1647.8213
☑	1194	824.9179	1647.8213
☑	1196	551.2881	1650.8424
☑	1197	827.4254	1652.8363
☑	1198	551.9958	1652.9655
☑	1199	552.6045	1654.7918
☑	1200	552.6046	1654.7920
☑	1201	552.6047	1654.7923
☑	1202	829.0844	1656.1543
☑	1203	554.6536	1660.9390
☑	1204	833.3851	1664.7556
☑	1212	834.3723	1666.7301
☑	1213	834.3801	1666.7456
☑	1214	834.3801	1666.7456
☑	1215	834.3802	1666.7458
☑	1216	834.3802	1666.7458
☑	1217	834.3802	1666.7458
☑	1218	834.3802	1666.7458
☑	1219	834.3802	1666.7458
☑	1220	834.3802	1666.7458
☑	1221	834.3803	1666.7460
☑	1222	834.3803	1666.7460
☑	1223	834.3803	1666.7460
☑	1224	556.5895	1666.7466
☑	1225	556.5898	1666.7477
☑	1226	556.5900	1666.7481
☑	1227	556.6224	1666.8453
☑	1228	834.4305	1666.8465
☑	1229	556.6230	1666.8471
☑	1230	556.6230	1666.8471
☑	1231	560.2938	1677.8597
☑	1232	840.4242	1678.8338
☑	1233	840.4243	1678.8341
☑	1234	840.4244	1678.8342
☑	1235	560.6187	1678.8344
☑	1236	560.6194	1678.8363
☑	1237	560.6199	1678.8379
☑	1238	560.6199	1678.8379
☑	1239	560.6199	1678.8379
☑	1240	560.6199	1678.8379
☑	1241	560.6199	1678.8379
☑	1242	560.6199	1678.8379
☑	1243	560.6199	1678.8379
☑	1244	560.6199	1678.8379
☑	1245	560.6199	1678.8379
☑	1246	560.6199	1678.8379
☑	1247	560.6200	1678.8381
☑	1248	560.9548	1679.8426
☑	1249	560.9582	1679.8527
☑	1250	421.5815	1682.2968
☑	1251	561.9427	1682.8064
☑	1253	565.2757	1692.8053
☑	1254	565.2770	1692.8093
☑	1255	848.3807	1694.7468
☑	1256	848.6268	1695.2391
☑	1257	566.9799	1697.9179
☑	1258	567.2906	1698.8499
☑	1259	567.3244	1698.9514
☑	1260	851.4560	1700.8974
☑	1261	567.9733	1700.8982
☑	1262	854.3924	1706.7702
☑	1263	854.4463	1706.8781
☑	1264	854.4466	1706.8785
☑	1265	569.9851	1706.9334
☑	1266	569.9857	1706.9352
☑	1267	569.9863	1706.9369
☑	1268	570.6235	1708.8487

☑	1269	570.6237	1708.8491
☑	1270	571.5897	1711.7473
☑	1271	856.8812	1711.7479
☑	1272	856.8816	1711.7487
☑	1273	856.8816	1711.7487
☑	1274	856.8817	1711.7489
☑	1275	571.5905	1711.7498
☑	1276	571.5905	1711.7498
☑	1277	571.5905	1711.7498
☑	1278	571.5905	1711.7498
☑	1279	571.5905	1711.7498
☑	1280	571.6119	1711.8138
☑	1281	857.3702	1712.7259
☑	1282	571.9304	1712.7693
☑	1283	572.0097	1713.0073
☑	1284	857.5114	1713.0083
☑	1285	857.5114	1713.0083
☑	1286	857.5114	1713.0083
☑	1287	857.5119	1713.0092
☑	1288	858.4826	1714.9506
☑	1289	572.6577	1714.9512
☑	1290	572.6577	1714.9512
☑	1291	858.4832	1714.9518
☑	1292	572.6582	1714.9527
☑	1293	572.6582	1714.9527
☑	1294	572.6582	1714.9527
☑	1295	572.6583	1714.9530
☑	1296	572.6583	1714.9530
☑	1297	572.6583	1714.9530
☑	1298	573.9291	1718.7654
☑	1299	573.9300	1718.7682
☑	1300	574.2273	1719.6601
☑	1301	574.2276	1719.6610
☑	1302	574.2782	1719.8128
☑	1303	574.2782	1719.8128
☑	1304	574.2803	1719.8191
☑	1305	574.6106	1720.8099
☑	1306	574.9827	1721.9263
☑	1307	431.4902	1721.9317
☑	1308	431.4902	1721.9317
☑	1309	431.4902	1721.9317
☑	1310	578.2563	1731.7470
☑	1311	578.2563	1731.7470
☑	1312	579.2992	1734.8759
☑	1313	872.9595	1743.9044
☑	1314	872.9603	1743.9061
☑	1315	583.9929	1748.9568
☑	1316	583.9933	1748.9580
☑	1317	583.9937	1748.9592
☑	1318	584.2838	1749.8294
☑	1319	585.9551	1754.8436
☑	1320	585.9558	1754.8455
☑	1321	586.3257	1755.9554
☑	1322	586.9715	1757.8928
☑	1323	586.9717	1757.8934
☑	1324	879.9549	1757.8953
☑	1325	879.9560	1757.8975
☑	1326	586.9732	1757.8977
☑	1327	586.9736	1757.8990
☑	1328	586.9736	1757.8990
☑	1329	586.9736	1757.8990
☑	1330	587.6137	1759.8192
☑	1331	587.9532	1760.8378
☑	1332	589.2271	1764.6594
☑	1333	589.3231	1764.9475
☑	1334	883.5058	1764.9971
☑	1335	883.5060	1764.9974
☑	1336	883.5061	1764.9976
☑	1337	883.5062	1764.9979
☑	1338	883.5063	1764.9980
☑	1339	883.5064	1764.9982
☑	1340	883.5067	1764.9988
☑	1341	883.5067	1764.9989
☑	1342	883.5067	1764.9989
☑	1343	883.5069	1764.9992
☑	1344	883.5069	1764.9992
☑	1345	883.5069	1764.9992
☑	1346	883.5069	1764.9992
☑	1347	883.5069	1764.9992
☑	1348	883.5069	1764.9992
☑	1349	883.5069	1764.9992
☑	1350	883.5069	1764.9992
☑	1351	883.5069	1764.9992
☑	1355	591.9147	1772.7223
☑	1356	592.3116	1773.9129
☑	1357	592.3124	1773.9154
☑	1358	592.3124	1773.9154
☑	1359	593.2838	1776.8295
☑	1360	594.9379	1781.7919
☑	1361	594.9382	1781.7927
☑	1362	595.2862	1782.8368
☑	1363	894.7133	1787.4121
☑	1364	597.2810	1788.8211
☑	1374	597.9530	1790.8372

☑	1375	*599.9810*	*1796.9211*
☑	1376	*600.2690*	*1797.7853*
☑	1377	*600.2708*	*1797.7907*
☑	1378	*600.2708*	*1797.7907*
☑	1379	*601.9724*	*1802.8955*
☑	1380	*604.6132*	*1810.8176*
☑	1381	*604.9662*	*1811.8769*
☑	1382	*604.9670*	*1811.8792*
☑	1383	*606.9784*	*1817.9134*
☑	1384	*606.9784*	*1817.9135*
☑	1385	*456.5008*	*1821.9741*
☑	1386	*456.5011*	*1821.9754*
☑	1387	*456.5012*	*1821.9756*
☑	1388	*608.6251*	*1822.8536*
☑	1389	*608.6595*	*1822.9567*
☑	1390	*456.9824*	*1823.9005*
☑	1391	*609.0004*	*1823.9793*
☑	1392	*609.6548*	*1825.9427*
☑	1393	*610.6389*	*1828.8949*
☑	1394	*615.2720*	*1842.7942*
☑	1395	*616.9553*	*1847.8442*
☑	1396	*463.6824*	*1850.7003*
☑	1397	*619.2766*	*1854.8079*
☑	1398	*619.2767*	*1854.8082*
☑	1399	*619.3420*	*1855.0041*
☑	1400	*931.9796*	*1861.9445*
☑	1402	*621.6572*	*1861.9499*
☑	1403	*621.6572*	*1861.9499*
☑	1404	*621.6572*	*1861.9499*
☑	1405	*621.9953*	*1862.9641*
☑	1406	*621.9960*	*1862.9663*
☑	1407	*932.9288*	*1863.8430*
☑	1408	*622.2885*	*1863.8438*
☑	1409	*622.2885*	*1863.8438*
☑	1410	*622.2885*	*1863.8438*
☑	1411	*622.2885*	*1863.8438*
☑	1412	*622.2886*	*1863.8440*
☑	1413	*622.2886*	*1863.8440*
☑	1414	*468.0219*	*1868.0587*
☑	1415	*626.9872*	*1877.9396*
☑	1416	*627.2894*	*1878.8463*
☑	1417	*940.4385*	*1878.8624*
☑	1418	*639.6213*	*1915.8422*
☑	1419	*639.9592*	*1916.8557*
☑	1420	*961.8154*	*1921.6162*
☑	1421	*641.6498*	*1921.9276*
☑	1422	*644.3944*	*1930.1613*
☑	1423	*644.3951*	*1930.1636*
☑	1424	*966.5020*	*1930.9894*
☑	1425	*967.0002*	*1931.9859*
☑	1426	*645.9798*	*1934.9177*
☑	1427	*646.3171*	*1935.9296*
☑	1428	*646.3176*	*1935.9309*
☑	1429	*646.3185*	*1935.9336*
☑	1430	*487.7442*	*1946.9479*
☑	1434	*652.3460*	*1954.0161*
☑	1435	*652.6660*	*1954.9762*
☑	1436	*652.6661*	*1954.9764*
☑	1437	*652.6666*	*1954.9779*
☑	1438	*652.6864*	*1955.0373*
☑	1439	*652.6865*	*1955.0377*
☑	1440	*652.6883*	*1955.0430*
☑	1442	*490.0197*	*1956.0499*
☑	1443	*490.0210*	*1956.0550*
☑	1444	*490.0218*	*1956.0583*
☑	1445	*653.6647*	*1957.9724*
☑	1446	*653.6665*	*1957.9777*
☑	1447	*656.3573*	*1966.0500*
☑	1448	*657.3424*	*1969.0054*
☑	1449	*659.3366*	*1974.9879*
☑	1450	*659.3366*	*1974.9881*
☑	1451	*659.3369*	*1974.9889*
☑	1452	*991.0228*	*1980.0311*
☑	1453	*991.0230*	*1980.0314*
☑	1454	*991.0233*	*1980.0321*
☑	1455	*661.0190*	*1980.0353*
☑	1456	*661.0195*	*1980.0365*
☑	1457	*661.3037*	*1980.8892*
☑	1458	*662.3105*	*1983.9098*
☑	1459	*498.2177*	*1988.8416*
☑	1461	*498.7541*	*1990.9874*
☑	1462	*664.6939*	*1991.0598*
☑	1463	*665.0493*	*1992.1262*
☑	1464	*665.3235*	*1992.9486*
☑	1465	*665.3252*	*1992.9537*
☑	1466	*665.3252*	*1992.9538*
☑	1467	*665.3295*	*1992.9667*
☑	1468	*502.7543*	*2006.9880*
☑	1469	*670.3053*	*2007.8942*
☑	1470	*671.0314*	*2010.0724*
☑	1471	*671.0316*	*2010.0730*
☑	1472	*671.0336*	*2010.0791*
☑	1473	*671.6754*	*2012.0043*
☑	1474	*671.6923*	*2012.0550*

☑	1475	671.6924	2012.0553
☑	1476	673.3006	2016.8799
☑	1477	673.3007	2016.8804
☑	1478	673.9601	2018.8584
☑	1479	673.9968	2018.9686
☑	1480	673.9968	2018.9686
☑	1481	674.0292	2019.0657
☑	1482	676.6930	2027.0571
☑	1483	676.6930	2027.0573
☑	1485	679.7206	2036.1399
☑	1486	1019.0780	2036.1414
☑	1487	679.7216	2036.1430
☑	1488	679.7222	2036.1446
☑	1490	682.0242	2043.0507
☑	1492	683.3552	2047.0439
☑	1493	684.3319	2049.9738
☑	1494	684.3326	2049.9760
☑	1495	686.3319	2055.9739
☑	1496	686.3321	2055.9744
☑	1497	686.3323	2055.9749
☑	1498	687.9704	2060.8892
☑	1499	688.6441	2062.9106
☑	1500	1032.4626	2062.9106
☑	1501	688.6442	2062.9107
☑	1502	1032.4629	2062.9112
☑	1503	1032.4629	2062.9112
☑	1504	1032.4629	2062.9112
☑	1505	1032.4629	2062.9112
☑	1506	1032.4629	2062.9112
☑	1507	1032.4629	2062.9112
☑	1508	1032.4629	2062.9112
☑	1509	1032.4629	2062.9112
☑	1510	688.6450	2062.9131
☑	1511	1032.4728	2062.9311
☑	1512	1032.9616	2063.9085
☑	1513	1032.9619	2063.9092
☑	1515	690.0055	2066.9946
☑	1516	690.0501	2067.1285
☑	1517	690.0503	2067.1292
☑	1518	690.0508	2067.1306
☑	1520	691.6825	2072.0256
☑	1521	692.3076	2073.9009
☑	1522	1044.8037	2087.5928
☑	1523	697.0066	2087.9979
☑	1524	697.3596	2089.0570
☑	1525	702.6938	2105.0595
☑	1526	703.0339	2106.0798
☑	1527	528.2292	2108.8877
☑	1528	704.0121	2109.0146
☑	1529	705.3700	2113.0883
☑	1530	529.7118	2114.8181
☑	1531	529.9628	2115.8221
☑	1532	706.2830	2115.8273
☑	1533	711.0430	2130.1073
☑	1534	1067.4617	2132.9088
☑	1535	1067.8292	2133.6438
☑	1536	429.5344	2142.6355
☑	1537	715.3690	2143.0852
☑	1538	1073.5017	2144.9888
☑	1539	1073.5038	2144.9931
☑	1540	1073.5048	2144.9951
☑	1541	716.0069	2144.9988
☑	1542	716.0070	2144.9992
☑	1543	722.3372	2163.9898
☑	1544	722.3373	2163.9899
☑	1545	723.0171	2166.0295
☑	1546	723.0185	2166.0336
☑	1547	723.9955	2168.9647
☑	1548	724.6425	2170.9057
☑	1549	727.0202	2178.0389
☑	1550	727.0214	2178.0425
☑	1551	728.0101	2181.0084
☑	1552	728.3660	2182.0762
☑	1553	728.7156	2183.1249
☑	1554	728.7156	2183.1249
☑	1555	546.7891	2183.1273
☑	1556	546.7902	2183.1317
☑	1557	546.7905	2183.1329
☑	1558	547.0361	2184.1151
☑	1559	733.6444	2197.9113
☑	1560	551.0365	2200.1169
☑	1561	736.0521	2205.1344
☑	1562	737.3723	2209.0951
☑	1563	737.3723	2209.0951
☑	1565	738.0033	2210.9882
☑	1566	738.0033	2210.9882
☑	1567	740.3650	2218.0731
☑	1568	746.3448	2236.0127
☑	1569	747.0535	2238.1388
☑	1570	747.0537	2238.1393
☑	1571	747.1067	2238.2984
☑	1572	747.1067	2238.2984
☑	1573	747.7109	2240.1108
☑	1574	747.7118	2240.1137

☑	1575	747.7119	2240.1138
☑	1576	561.0451	2240.1513
☑	1577	561.2924	2241.1404
☑	1578	749.6908	2246.0505
☑	1579	752.0435	2253.1086
☑	1580	752.3850	2254.1331
☑	1581	752.3855	2254.1348
☑	1582	565.2932	2257.1439
☑	1583	757.4111	2269.2115
☑	1584	757.4119	2269.2138
☑	1585	757.4120	2269.2142
☑	1586	758.7091	2273.1055
☑	1587	759.0516	2274.1329
☑	1588	760.7519	2279.2339
☑	1589	761.0583	2280.1530
☑	1590	763.2913	2286.8522
☑	1591	763.6328	2287.8766
☑	1592	573.7700	2291.0508
☑	1593	765.3476	2293.0210
☑	1594	574.2632	2293.0238
☑	1595	766.0177	2295.0313
☑	1596	771.3930	2311.1571
☑	1597	776.4114	2326.2123
☑	1598	778.7493	2333.2261
☑	1599	585.3036	2337.1853
☑	1600	588.7378	2350.9222
☑	1601	786.0408	2355.1006
☑	1603	786.0412	2355.1019
☑	1604	789.3988	2365.1745
☑	1605	789.7088	2366.1046
☑	1606	594.8181	2375.2432
☑	1607	796.0400	2385.0982
☑	1608	798.0501	2391.1285
☑	1609	603.0094	2408.0085
☑	1610	610.2985	2437.1650
☑	1611	610.2985	2437.1650
☑	1612	614.2993	2453.1680
☑	1613	616.0615	2460.2169
☑	1614	616.7779	2463.0824
☑	1615	620.0609	2476.2145
☑	1616	620.0609	2476.2145
☑	1617	826.7232	2477.1477
☑	1618	826.7232	2477.1479
☑	1619	832.1052	2493.2939
☑	1620	624.8076	2495.2015
☑	1621	833.0740	2496.2002
☑	1622	834.3963	2500.1671
☑	1623	834.4122	2500.2147
☑	1624	836.0714	2505.1923
☑	1625	836.0717	2505.1932
☑	1626	836.9826	2507.9259
☑	1627	837.0756	2508.2049
☑	1628	628.3578	2509.4019
☑	1629	838.4076	2512.2011
☑	1630	838.4077	2512.2012
☑	1631	842.0813	2523.2221
☑	1632	842.0823	2523.2250
☑	1633	634.3157	2533.2336
☑	1634	634.3164	2533.2366
☑	1635	634.3166	2533.2371
☑	1637	845.7318	2534.1734
☑	1638	849.7527	2546.2362
☑	1639	849.7961	2546.3663
☑	1640	850.1328	2547.3766
☑	1641	638.3128	2549.2222
☑	1642	640.8077	2559.2017
☑	1643	858.4120	2572.2141
☑	1644	869.1430	2604.4071
☑	1645	869.1447	2604.4122
☑	1646	871.7517	2612.2332
☑	1649	654.0679	2612.2425
☑	1650	654.0679	2612.2425
☑	1652	654.0679	2612.2425
☑	1653	654.0679	2612.2425
☑	1654	654.3134	2613.2246
☑	1655	657.0540	2624.1870
☑	1656	888.1503	2661.4291
☑	1657	668.5512	2670.1757
☑	1658	668.5513	2670.1761
☑	1659	668.5514	2670.1766
☑	1660	672.5762	2686.2756
☑	1661	541.5694	2702.8106
☑	1662	902.4244	2704.2513
☑	1663	904.4239	2710.2499
☑	1664	904.7652	2711.2739
☑	1665	682.8056	2727.1933
☑	1666	682.8057	2727.1938
☑	1667	684.3189	2733.2464
☑	1668	690.2942	2757.1475
☑	1669	923.1093	2766.3061
☑	1670	693.2914	2769.1363
☑	1676	961.8133	2882.4179
☑	1677	734.8674	2935.4405
☑	1679	734.8698	2935.4502

☑	1680	*980.1579*	*2937.4519*
☑	1681	*748.0944*	*2988.3486*
☑	1682	*770.1953*	*3076.7522*
☑	1683	*774.5428*	*3094.1423*
☑	1684	*1044.8027*	*3131.3862*
☑	1686	*807.9072*	*3227.5996*
☑	1687	*818.1549*	*3268.5907*
☑	1688	*833.3865*	*3329.5171*
☑	1689	*834.1223*	*3332.4603*
☑	1690	*838.1867*	*3348.7179*
☑	1691	*862.6314*	*3446.4963*
☑	1692	*597.1167*	*3576.6567*
☑	1693	*911.6700*	*3642.6508*
☑	1694	*911.6700*	*3642.6508*
☑	1695	*525.7616*	*3673.2799*
☑	1696	*925.9234*	*3699.6646*
☑	1697	*925.9237*	*3699.6657*
☑	1698	*636.6457*	*3813.8306*
☑	1699	*658.2039*	*3943.1800*
☑	1700	*702.3605*	*4208.1190*
☑	1701	*658.0546*	*4599.3313*
☑	1702	*621.4548*	*4963.5804*

Search Parameters

```
Type of search         : MS/MS Ion Search
Enzyme                 : Trypsin
Variable modifications : Carbamidomethyl (C),Oxidation (M)
Mass values            : Monoisotopic
Protein Mass           : Unrestricted
Peptide Mass Tolerance : ± 50 ppm
Fragment Mass Tolerance: ± 0.6 Da
Max Missed Cleavages   : 1
Instrument type        : ESI-QUAD-TOF
Number of queries      : 1702
```

Mascot: http://www.matrixscience.com/

S4 Fig MASCOT Search Results – SEC Sample

Mascot Search Results

**User**	:	
**Email**	:	
**Search title**	:	**Submitted from Sheep Rerun-2 by Mascot Daemon on APAF-WS-08**
**MS data file**	:	**\\apaf-hpv-file\projects\External\e_17906_VUW_SandiDempsey_20150615\1_MassSpec\5600\Run2\RawData\150713_P17906_SD97-1_1.MGF**
**Database**	:	**sp_sheep_140625 sheep_140625_ (647 sequences; 258179 residues)**
**Timestamp**	:	**13 Jul 2015 at 22:49:26 GMT**
**Protein hits**	:	**Q9BGM5** sp|Q9BGM5|K1C25_SHEEP Keratin, type I cytoskeletal 25 OS=Ovis aries GN=KRT25 PE=2 SV=1
		**Q9TTE2** sp|Q9TTE2|PGS2_SHEEP Decorin OS=Ovis aries GN=DCN PE=2 SV=1
		**Q28554** sp|Q28554|G3P_SHEEP Glyceraldehyde-3-phosphate dehydrogenase (Fragment) OS=Ovis aries GN=GAPDH PE=2 SV=4
		**P60713** sp|P60713|ACTB_SHEEP Actin, cytoplasmic 1 OS=Ovis aries GN=ACTB PE=2 SV=1
		**Q6B7M7** sp|Q6B7M7|COF1_SHEEP Cofilin-1 OS=Ovis aries GN=CFL1 PE=2 SV=3

	sp_sheep_140625	Decoy	False discovery rate
Peptide matches above identity threshold	6	1	16.67 %
Peptide matches above homology or identity threshold	6	1	16.67 %

Mascot Score Histogram

Ions score is -10*Log(P), where P is the probability that the observed match is a random event.
Individual ions scores > 12 indicate identity or extensive homology (p<0.05).
Protein scores are derived from ions scores as a non-probabilistic basis for ranking protein hits.

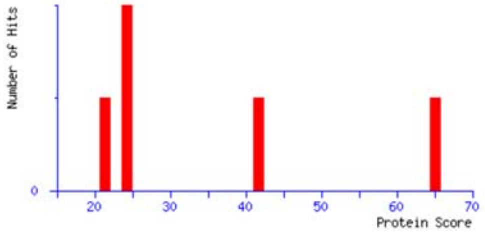

Peptide Summary Report

Format As: Peptide Summary | Help

Significance threshold p< 0.05 | Max. number of hits AUTO | Show Percolator scores

Standard scoring | MudPIT scoring | Ions score or expect cut-off 12 | Show sub-sets 0

Show pop-ups | Suppress pop-ups | Sort unassigned Decreasing Score | Require bold red

Select All | Select None | Search Selected | **Error tolerant** | Archive Report

1. Q9BGM5 **Mass:** 49282 **Score:** 65 **Matches:** 2(2) **Sequences:** 2(2) **emPAI:** 0.14
sp|Q9BGM5|K1C25_SHEEP Keratin, type I cytoskeletal 25 OS=Ovis aries GN=KRT25 PE=2 SV=1
Check to include this hit in error tolerant search or archive report

Query	Observed	Mr(expt)	Mr(calc)	ppm	Miss	Score	Expect	Rank	Unique	Peptide
152	355.5414	1063.6023	1063.5662	33.9	0	56	2.4e-006	1	U	R.LASYLENVR.A
173	555.2561	1108.4977	1108.4825	13.7	0	24	0.012	1	U	R.DAEAWFNEK.S

2. Q9TTE2 **Mass:** 39947 **Score:** 42 **Matches:** 1(1) **Sequences:** 1(1) **emPAI:** 0.08
sp|Q9TTE2|PGS2_SHEEP Decorin OS=Ovis aries GN=DCN PE=2 SV=1
Check to include this hit in error tolerant search or archive report

Query	Observed	Mr(expt)	Mr(calc)	ppm	Miss	Score	Expect	Rank	Unique	Peptide
356	624.3236	1246.6327	1246.6227	7.99	0	42	0.00015	1	U	R.VVQCSDLGLEK.V + Carbamidomethyl (C)

3. Q28554 **Mass:** 34710 **Score:** 25 **Matches:** 1(1) **Sequences:** 1(1) **emPAI:** 0.10
sp|Q28554|G3P_SHEEP Glyceraldehyde-3-phosphate dehydrogenase (Fragment) OS=Ovis aries GN=GAPDH PE=2 SV=4
Check to include this hit in error tolerant search or archive report

Query	Observed	Mr(expt)	Mr(calc)	ppm	Miss	Score	Expect	Rank	Unique	Peptide
438	685.3836	1368.7527	1368.7361	12.1	0	25	0.0034	1	U	R.GAAQNIIPASTGAAK.A

4. P60713 **Mass:** 41710 **Score:** 24 **Matches:** 1(1) **Sequences:** 1(1) **emPAI:** 0.08
sp|P60713|ACTB_SHEEP Actin, cytoplasmic 1 OS=Ovis aries GN=ACTB PE=2 SV=1
Check to include this hit in error tolerant search or archive report

Query	Observed	Mr(expt)	Mr(calc)	ppm	Miss	Score	Expect	Rank	Unique	Peptide
252	589.3136	1176.6126	1176.6060	5.60	0	24	0.0078	1	U	K.EITALAPSTMK.I + Oxidation (M)

5. Q6B7M7 **Mass:** 18507 **Score:** 21 **Matches:** 1(1) **Sequences:** 1(1) **emPAI:** 0.18
sp|Q6B7M7|COF1_SHEEP Cofilin-1 OS=Ovis aries GN=CFL1 PE=2 SV=3
Check to include this hit in error tolerant search or archive report

Query	Observed	Mr(expt)	Mr(calc)	ppm	Miss	Score	Expect	Rank	Unique	Peptide
429	669.3204	1336.6263	1336.6187	5.72	0	21	0.013	1	U	R.YALYDATYETK.E

Peptide matches not assigned to protein hits: (no details means no match)

Query	Observed	Mr(expt)	Mr(calc)	ppm	Miss	Score	Expect	Rank	Unique	Peptide
107	464.2733	926.5319	926.5450	-14.10	1	12	0.064	1		KHFLQVR
224	579.8401	1157.6657	1157.6404	21.9	1	12	0.069	1		DSGRALAEVLK
119	487.2704	972.5262	972.4876	39.7	0	11	0.074	1		LLEGEEQR
183	559.8208	1117.6270	1117.6132	12.4	0	10	0.096	1		TALSVVTWNK
389	635.3687	1268.7229	1268.6837	30.9	1	10	0.11	1		ASELARAEGIPR
413	653.3311	1304.6477	1304.7088	-46.83	0	8	0.41	1		HLVDEPQNLIK
451	706.8388	1411.6631	1411.6337	20.8	1	7	0.2	1		SRGIVEECCFR + 2 Carbamidomethyl (C)
52	428.7669	855.5193	855.4926	31.1	1	6	0.25	1		LRISPDR
176	556.8353	2223.3123	2223.2773	15.7	1	5	0.3	1		IGCTPVLVLSGLDTIRQALVR
145	523.2867	1044.5589	1044.5862	-26.18	1	5	0.31	1		LSRLQCLR + Carbamidomethyl (C)
156	537.7639	1073.5133	1073.4699	40.4	0	5	0.32	1		QESEEPPMK
390	637.3201	1272.6257	1272.6826	-44.72	1	5	0.38	1		GHLYTLAKENK
89	450.7640	899.5134	899.5188	-6.00	1	4	0.56	1		QVLEAGKR
291	601.8473	1201.6800	1201.6853	-4.40	1	3	1.1	1		KLLSMIGGVER
230	580.8443	1159.6741	1159.6271	40.6	1	3	0.54	1		CVVLAEDKVK + Carbamidomethyl (C)
538	963.9523	1925.8900	1925.8822	4.02	1	2	0.88	1		MDSQMTKQALNEIETR + 2 Oxidation (M)
289	601.3000	1200.5854	1200.6098	-20.39	0	2	1.4	1		QSVEADINGLR
222	579.3103	1156.6060	1156.6200	-12.09	1	2	0.7	1		QEEVQLQKR
229	580.8438	1159.6730	1159.6271	39.6	1	2	0.65	1		CVVLAEDKVK + Carbamidomethyl (C)
180	558.8259	1115.6373	1115.5836	48.1	1	2	0.65	1		GWLDTVRNR
179	558.8259	1115.6373	1115.5836	48.1	1	2	0.66	1		GWLDTVRNR
276	595.8478	1189.6811	1189.6489	27.1	0	2	0.66	1		TVSALMVGLQR + Oxidation (M)
284	598.9487	1793.8244	1793.7924	17.8	0	2	0.68	1		DGMQEEAIQEIAGMTR + Oxidation (M)
120	494.2995	986.5845	986.5661	18.6	1	1	0.88	1		TAVWVQRK
228	580.8422	1159.6698	1159.6271	36.8	1	1	0.8	1		CVVLAEDKVK + Carbamidomethyl (C)
223	579.8370	1157.6595	1157.6404	16.5	1	1	0.82	1		DSGRALAEVLK
108	464.7770	927.5395	927.5072	34.8	0	1	0.93	1		CVQRPLR + Carbamidomethyl (C)
210	573.3356	1144.6566	1144.6029	46.9	1	0	0.91	1		VEGWVWKNK
51	428.7669	855.5193	855.5542	-40.79	1	0	0.93	1		LLDRLVK
361	625.3611	1248.7076	1248.7077	-0.13	1	0	0.96	1		ATVEALINKYK
50	428.7668	855.5191	855.5542	-40.98	1	0	0.97	1		LLDRLVK
141	519.2663	1554.7772	1554.7435	21.6	1	0	2.6	1		EIVCFQGFRTPCR
160	543.3153	1084.6160	1084.5989	15.8	1	0	1	1		DEARVLQVR
1	360.2056	718.3965								
2	362.2206	722.4266								
3	362.2212	722.4279								
4	382.2200	762.4254								
5	382.2230	762.4314								
6	384.2343	766.4540								
7	384.2356	766.4566								
8	404.2325	806.4505								
9	406.2473	810.4801								
10	406.2483	810.4820								
11	412.7521	823.4896								
12	412.7533	823.4920								
13	412.7533	823.4920								
14	412.7544	823.4942								
15	414.2448	826.4751								
16	416.2269	830.4393								
17	416.2495	830.4845								
18	420.4436	838.8727								
19	420.4436	838.8727								
20	421.7571	841.4996								
21	421.7580	841.5015								
22	421.7582	841.5018								
23	421.7582	841.5019								
24	421.7583	841.5020								
25	421.7583	841.5020								
26	421.7584	841.5023								
27	421.7585	841.5024								
28	421.7586	841.5026								
29	421.7587	841.5028								
30	421.7587	841.5028								
31	421.7587	841.5028								
32	421.7588	841.5030								
33	421.7589	841.5032								
34	421.7590	841.5034								
35	426.2478	850.4810								
36	427.4293	852.8440								
37	428.2450	854.4754								
38	428.2459	854.4772								
39	428.2464	854.4782								
40	428.2471	854.4797								
41	428.2475	854.4805								
42	428.2480	854.4815								
43	428.2520	854.4895								
44	428.2618	854.5091								
45	428.2622	854.5098								
46	428.7654	855.5162								
47	428.7656	855.5166								
48	428.7656	855.5166								
49	428.7668	855.5191								
53	428.7670	855.5195								
54	429.2380	856.4614								
55	434.4142	866.8138								
56	434.7681	867.5216								

☑	57	435.2574	868.5002
☑	58	435.2592	868.5038
☑	59	435.7562	869.4978
☑	60	435.7736	869.5327
☑	61	435.7738	869.5331
☑	62	435.7741	869.5337
☑	63	435.7745	869.5344
☑	64	435.7745	869.5344
☑	65	435.7747	869.5349
☑	66	435.7747	869.5349
☑	67	435.7748	869.5351
☑	68	437.2350	872.4554
☑	69	441.2628	880.5111
☑	70	442.2643	882.5141
☑	71	442.5896	883.1647
☑	72	442.7629	883.5112
☑	73	448.2583	894.5020
☑	74	448.8663	895.7180
☑	75	449.7724	897.5302
☑	76	450.2695	898.5245
☑	77	450.2696	898.5247
☑	78	450.2699	898.5252
☑	79	450.2699	898.5252
☑	80	450.2700	898.5254
☑	81	450.2710	898.5275
☑	82	450.2715	898.5285
☑	83	450.2724	898.5303
☑	84	450.2747	898.5348
☑	85	450.2750	898.5354
☑	86	450.2752	898.5357
☑	87	450.2752	898.5358
☑	88	450.2984	898.5822
☑	90	455.2437	908.4728
☑	91	456.2783	910.5420
☑	92	456.2793	910.5440
☑	93	456.2797	910.5449
☑	94	456.2798	910.5451
☑	95	456.2807	910.5468
☑	96	456.7796	911.5447
☑	97	456.7800	911.5455
☑	98	457.2073	912.4000
☑	99	457.2741	912.5337
☑	100	457.2783	912.5420
☑	101	457.2784	912.5423
☑	102	457.2912	912.5677
☑	103	457.7781	913.5416
☑	104	462.7805	923.5464
☑	105	463.2797	924.5448
☑	106	464.2515	926.4885
☑	109	466.2636	930.5126
☑	110	466.7819	931.5493
☑	111	471.2605	940.5064
☑	112	472.2701	942.5255
☑	113	472.2858	942.5570
☑	114	472.2890	942.5635
☑	115	476.2616	950.5086
☑	116	476.7778	951.5410
☑	117	476.7784	951.5423
☑	118	486.2933	970.5721
☑	121	494.3024	986.5902
☑	122	501.2332	1000.4518
☑	123	501.2705	1000.5265
☑	124	501.3257	1000.6369
☑	125	501.7352	1001.4559
☑	126	503.2449	1004.4752
☑	127	503.2485	1004.4825
☑	128	503.7649	1005.5152
☑	129	507.2085	1012.4025
☑	130	508.2940	1014.5734
☑	131	510.7764	1019.5382
☑	132	513.2406	1024.4666
☑	133	513.2406	1024.4667
☑	134	513.3044	1024.5942
☑	135	514.2709	1026.5272
☑	136	516.3145	1030.6145
☑	137	516.3150	1030.6155
☑	138	516.8247	1031.6349
☑	139	518.3100	1034.6055
☑	140	518.7106	1035.4066
☑	142	521.2375	1040.4604
☑	143	523.2753	1044.5360
☑	144	523.2850	1044.5554
☑	146	526.2757	1050.5367
☑	147	529.2239	1056.4333
☑	148	529.2553	1056.4961
☑	149	530.2902	1058.5659
☑	150	530.7851	1059.5557
☑	151	530.7858	1059.5570

☑	153	534.8206	1067.6266
☑	154	536.3266	1070.6387
☑	155	536.3270	1070.6394
☑	157	538.3274	1074.6403
☑	158	538.3283	1074.6420
☑	159	541.7518	1081.4891
☑	161	543.8202	1085.6259
☑	162	544.8021	1087.5897
☑	163	544.8271	1087.6397
☑	164	545.3020	1088.5895
☑	165	545.3526	1088.6907
☑	166	545.8223	1089.6300
☑	167	546.3338	1090.6531
☑	168	550.8213	1099.6281
☑	169	551.7969	1101.5793
☑	170	551.8197	1101.6249
☑	171	551.8226	1101.6307
☑	172	551.8237	1101.6329
☑	174	555.8231	1109.6317
☑	175	556.8319	1111.6492
☑	177	557.8220	1113.6295
☑	178	557.8234	1113.6322
☑	181	558.8303	1115.6461
☑	182	559.3280	1116.6415
☑	184	560.3304	1118.6462
☑	185	560.3390	1118.6634
☑	186	562.3300	1122.6454
☑	187	563.8380	1125.6615
☑	188	564.3282	1126.6418
☑	189	565.3071	1128.5996
☑	190	565.8318	1129.6490
☑	191	565.8348	1129.6550
☑	192	565.8352	1129.6558
☑	193	565.8354	1129.6562
☑	194	565.8355	1129.6564
☑	195	565.8419	1129.6692
☑	196	566.2613	1130.5080
☑	197	566.3394	1130.6642
☑	198	566.8438	1131.6730
☑	199	566.8438	1131.6730
☑	200	567.3142	1132.6138
☑	201	567.3161	1132.6176
☑	202	569.2810	1136.5475
☑	203	570.7925	1139.5705
☑	204	571.3544	1140.6942
☑	205	571.3549	1140.6952
☑	206	571.8342	1141.6538
☑	207	571.8423	1141.6701
☑	208	571.8455	1141.6763
☑	209	573.2936	1144.5727
☑	211	573.8368	1145.6591
☑	212	574.8440	1147.6735
☑	213	574.8442	1147.6738
☑	214	574.8442	1147.6739
☑	215	574.8443	1147.6741
☑	216	574.8443	1147.6741
☑	217	575.2963	1148.5781
☑	218	578.3378	1154.6610
☑	219	578.8311	1155.6475
☑	220	578.8356	1155.6565
☑	221	579.2709	1156.5271
☑	225	580.3302	1158.6459
☑	226	580.3413	1158.6680
☑	227	580.8417	1159.6688
☑	231	580.8454	1159.6762
☑	232	581.3444	1160.6743
☑	233	582.2831	1162.5516
☑	234	582.3402	1162.6658
☑	235	582.3495	1162.6845
☑	236	582.3518	1162.6890
☑	237	582.8458	1163.6770
☑	238	586.3163	1170.6180
☑	239	586.8434	1171.6723
☑	240	586.8439	1171.6733
☑	241	586.8458	1171.6770
☑	242	586.8462	1171.6778
☑	243	587.3436	1172.6725
☑	244	587.3436	1172.6725
☑	245	588.3511	1174.6877
☑	246	588.8406	1175.6666
☑	247	588.8409	1175.6672
☑	248	588.8432	1175.6718
☑	249	588.8543	1175.6940
☑	250	588.8570	1175.6995
☑	251	589.2392	1176.4638
☑	253	589.3140	1176.6134
☑	254	589.3310	1176.6475
☑	255	589.3418	1176.6691

☑	256	589.3759	1176.7372
☑	257	589.8566	1177.6986
☑	258	590.3057	1178.5968
☑	259	590.3063	1178.5980
☑	260	590.3113	1178.6080
☑	261	590.8153	1179.6160
☑	262	591.3365	1180.6584
☑	263	592.3424	1182.6703
☑	264	592.8661	1183.7176
☑	265	593.3375	1184.6605
☑	266	593.8499	1185.6852
☑	267	594.3458	1186.6771
☑	268	594.3492	1186.6839
☑	269	594.3494	1186.6843
☑	270	594.8437	1187.6729
☑	271	594.8451	1187.6757
☑	272	594.8517	1187.6889
☑	273	594.8545	1187.6944
☑	274	595.2688	1188.5231
☑	275	595.8469	1189.6792
☑	277	595.8490	1189.6834
☑	278	596.8372	1191.6599
☑	279	597.2375	1192.4604
☑	280	597.2379	1192.4613
☑	281	597.3103	1192.6061
☑	282	597.8222	1193.6299
☑	283	597.8465	1193.6785
☑	285	600.8415	1199.6685
☑	286	600.8482	1199.6818
☑	287	600.8495	1199.6845
☑	288	600.8590	1199.7035
☑	290	601.3588	1200.7031
☑	292	601.8526	1201.6906
☑	293	602.3499	1202.6853
☑	294	602.8539	1203.6933
☑	295	602.8566	1203.6987
☑	296	602.8573	1203.7001
☑	297	603.2547	1204.4949
☑	298	603.2957	1204.5768
☑	299	603.3582	1204.7018
☑	300	603.3585	1204.7024
☑	301	603.3586	1204.7026
☑	302	603.8613	1205.7080
☑	303	604.3618	1206.7090
☑	304	604.3674	1206.7203
☑	305	604.3685	1206.7224
☑	306	605.2790	1208.5434
☑	307	607.3399	1212.6652
☑	308	607.8674	1213.7202
☑	309	608.1772	1214.3398
☑	310	608.3327	1214.6508
☑	311	608.3423	1214.6699
☑	312	608.8467	1215.6788
☑	313	609.3528	1216.6910
☑	314	609.3541	1216.6935
☑	315	609.8574	1217.7002
☑	316	609.8589	1217.7032
☑	317	609.8607	1217.7068
☑	318	609.8610	1217.7075
☑	319	609.8641	1217.7137
☑	320	610.8682	1219.7218
☑	321	611.3289	1220.6432
☑	322	611.3590	1220.7034
☑	323	611.3594	1220.7043
☑	324	611.3621	1220.7096
☑	325	611.8613	1221.7080
☑	326	611.8644	1221.7142
☑	327	612.8572	1223.6999
☑	328	614.3286	1226.6426
☑	329	615.3538	1228.6931
☑	330	615.3578	1228.7010
☑	331	616.3465	1230.6784
☑	332	616.3519	1230.6893
☑	333	616.3522	1230.6898
☑	334	616.8575	1231.7004
☑	335	617.3207	1232.6269
☑	336	617.3497	1232.6849
☑	337	412.2313	1233.6722
☑	338	617.8609	1233.7073
☑	339	617.8624	1233.7103
☑	340	617.8732	1233.7318
☑	341	618.2926	1234.5705
☑	342	618.2943	1234.5741
☑	343	618.3578	1234.7010
☑	344	618.3609	1234.7071
☑	345	618.3612	1234.7079
☑	346	618.8667	1235.7189
☑	347	619.3522	1236.6898

348	622.8579	1243.7013
349	622.8615	1243.7084
350	622.8674	1243.7203
351	623.3575	1244.7004
352	623.3608	1244.7070
353	623.8531	1245.6916
354	623.8627	1245.7109
355	623.8634	1245.7123
357	624.3581	1246.7016
358	624.3605	1246.7064
359	624.8600	1247.7055
360	624.8686	1247.7227
362	625.8637	1249.7128
363	625.8764	1249.7383
364	625.8845	1249.7544
365	627.3682	1252.7218
366	627.8108	1253.6070
367	628.8849	1255.7553
368	628.8858	1255.7570
369	628.8872	1255.7599
370	629.2723	1256.5300
371	629.2821	1256.5497
372	629.3208	1256.6271
373	630.3598	1258.7050
374	630.3636	1258.7127
375	630.3640	1258.7134
376	630.3655	1258.7165
377	630.3675	1258.7205
378	630.8429	1259.6712
379	631.3365	1260.6585
380	631.3565	1260.6985
381	631.8629	1261.7113
382	631.8779	1261.7412
383	631.8782	1261.7417
384	632.8619	1263.7093
385	633.3193	1264.6241
386	633.3563	1264.6981
387	633.3691	1264.7236
388	634.8679	1267.7212
391	637.8777	1273.7407
392	638.3339	1274.6533
393	638.3648	1274.7150
394	638.3698	1274.7251
395	638.8735	1275.7325
396	639.8773	1277.7400
397	640.3749	1278.7353
398	640.8720	1279.7295
399	640.8763	1279.7381
400	642.3653	1282.7160
401	643.3347	1284.6547
402	644.8806	1287.7466
403	645.3456	1288.6767
404	646.0210	1290.0275
405	646.2262	1290.4378
406	646.2848	1290.5551
407	646.3751	1290.7356
408	647.3667	1292.7189
409	648.3965	1294.7784
410	650.8646	1299.7146
411	651.3259	1300.6373
412	652.8804	1303.7462
414	653.4738	1304.9330
415	654.3556	1306.6967
416	654.3682	1306.7218
417	655.3330	1308.6515
418	655.3404	1308.6663
419	655.3421	1308.6696
420	655.3501	1308.6857
421	655.3851	1308.7557
422	655.3890	1308.7634
423	655.8863	1309.7581
424	438.9302	1313.7688
425	659.8504	1317.6862
426	665.4228	1328.8310
427	666.3697	1330.7249
428	446.2424	1335.7054
430	669.8388	1337.6630
431	670.3713	1338.7279
432	670.8533	1339.6920
433	672.3747	1342.7348
434	677.3805	1352.7464
435	677.3823	1352.7499
436	678.2536	1354.4927
437	678.3721	1354.7297
439	687.8404	1373.6663
440	688.3683	1374.7220
441	691.3329	1380.6513
442	692.3652	1382.7158

☑	443	465.2499	1392.7279
☑	444	465.2507	1392.7302
☑	445	698.3146	1394.6147
☑	446	699.3677	1396.7208
☑	447	699.4088	1396.8030
☑	448	705.3689	1408.7232
☑	449	705.8503	1409.6860
☑	450	706.3321	1410.6497
☑	452	708.3721	1414.7297
☑	453	472.5869	1414.7390
☑	454	710.3311	1418.6476
☑	455	710.4014	1418.7882
☑	456	713.3557	1424.6969
☑	457	714.4372	1426.8598
☑	458	716.3756	1430.7367
☑	459	721.3564	1440.6982
☑	460	721.3955	1440.7765
☑	461	723.3259	1444.6372
☑	462	723.8850	1445.7554
☑	463	725.8259	1449.6372
☑	464	728.3043	1454.5941
☑	465	730.3561	1458.6976
☑	466	732.4288	1462.8431
☑	467	733.3669	1464.7192
☑	468	733.8514	1465.6883
☑	469	734.3610	1466.7074
☑	470	736.3605	1470.7064
☑	471	738.4021	1474.7896
☑	472	738.4021	1474.7897
☑	473	740.4088	1478.8031
☑	474	743.3755	1484.7364
☑	475	743.9291	1485.8436
☑	476	744.3498	1486.6850
☑	477	748.3425	1494.6705
☑	478	749.3936	1496.7725
☑	479	749.8572	1497.6997
☑	480	751.3721	1500.7297
☑	481	751.3859	1500.7573
☑	482	753.8383	1505.6620
☑	483	756.3698	1510.7250
☑	484	756.3699	1510.7252
☑	485	756.3728	1510.7311
☑	486	757.3895	1512.7645
☑	487	769.3306	1536.6466
☑	488	514.6159	1540.8259
☑	489	779.3141	1556.6136
☑	490	780.4111	1558.8077
☑	491	781.4153	1560.8161
☑	492	781.9159	1561.8172
☑	493	783.3971	1564.7796
☑	494	786.4080	1570.8014
☑	495	797.3834	1592.7522
☑	496	820.3585	1638.7024
☑	497	825.4234	1648.8323
☑	498	829.9136	1657.8127
☑	499	834.3987	1666.7829
☑	500	836.0096	1670.0047
☑	501	847.3882	1692.7618
☑	502	854.3988	1706.7831
☑	503	854.8991	1707.7837
☑	504	882.4129	1762.8111
☑	505	883.3811	1764.7476
☑	506	891.3800	1780.7455
☑	507	897.9192	1793.8238
☑	508	897.9192	1793.8239
☑	509	898.4197	1794.8248
☑	510	905.4079	1808.8013
☑	511	905.4206	1808.8266
☑	512	604.2755	1809.8047
☑	513	905.9154	1809.8163
☑	514	905.9195	1809.8244
☑	515	906.4302	1810.8458
☑	516	906.4353	1810.8560
☑	517	604.9624	1811.8654
☑	518	913.4173	1824.8200
☑	519	913.4565	1824.8983
☑	520	617.2640	1848.7703
☑	521	926.4317	1850.8489
☑	522	926.4373	1850.8601
☑	523	623.3180	1866.9322
☑	524	946.9709	1891.9273
☑	525	952.4783	1902.9421
☑	526	954.9675	1907.9204
☑	527	637.2985	1908.8737
☑	528	637.6340	1909.8801
☑	529	955.9503	1909.8860
☑	530	960.4247	1918.8348
☑	531	963.4527	1924.8908

☑	532	642.9648	1925.8726
☑	533	642.9677	1925.8813
☑	534	963.9484	1925.8823
☑	535	963.9488	1925.8831
☑	536	963.9489	1925.8833
☑	537	963.9493	1925.8841
☑	539	964.4288	1926.8429
☑	540	964.4593	1926.9041
☑	541	967.4280	1932.8414
☑	542	970.5015	1938.9884
☑	543	970.5038	1938.9930
☑	544	647.6509	1939.9309
☑	545	971.3960	1940.7775
☑	546	971.4308	1940.8471
☑	547	971.4374	1940.8603
☑	548	971.4611	1940.9077
☑	549	971.4775	1940.9404
☑	550	648.2967	1941.8684
☑	551	648.2968	1941.8686
☑	552	648.2975	1941.8706
☑	553	971.9457	1941.8767
☑	554	971.9460	1941.8774
☑	555	971.9462	1941.8779
☑	556	971.9464	1941.8782
☑	557	971.9481	1941.8816
☑	558	972.4384	1942.8623
☑	559	648.6328	1942.8766
☑	560	653.0282	1956.0628
☑	561	653.2974	1956.8703
☑	562	979.4456	1956.8766
☑	563	979.4880	1956.9614
☑	564	653.6275	1957.8607
☑	565	979.9385	1957.8625
☑	566	979.9399	1957.8652
☑	567	980.4461	1958.8777
☑	568	980.4484	1958.8822
☑	569	984.4604	1966.9063
☑	570	987.4704	1972.9263
☑	571	987.9399	1973.8653
☑	572	992.4566	1982.8987
☑	573	992.9629	1983.9113
☑	574	1006.5259	2011.0372
☑	575	1014.0132	2026.0119
☑	576	1014.5153	2027.0161
☑	577	1014.5286	2027.0426
☑	578	1021.5074	2041.0003
☑	579	1021.5133	2041.0121
☑	580	1026.4195	2050.8244
☑	581	1027.4353	2052.8560
☑	582	1031.4758	2060.9371
☑	583	1031.4810	2060.9474
☑	584	1031.4815	2060.9484
☑	585	688.3205	2061.9397
☑	586	1032.4756	2062.9367
☑	587	1032.4762	2062.9378
☑	588	1032.4821	2062.9497
☑	589	1039.4589	2076.9033
☑	590	1039.5100	2077.0055
☑	591	693.6316	2077.8730
☑	592	1039.9492	2077.8838
☑	593	1040.4568	2078.8989
☑	594	1040.4611	2078.9077
☑	595	1044.0275	2086.0404
☑	596	1047.4680	2092.9215
☑	597	1047.5042	2092.9939
☑	598	698.9626	2093.8659
☑	599	1047.9443	2093.8740
☑	600	1047.9459	2093.8773
☑	601	699.2924	2094.8553
☑	602	699.2971	2094.8693
☑	603	1048.4425	2094.8705
☑	604	1048.4509	2094.8872
☑	605	1050.5253	2099.0360
☑	606	1050.5402	2099.0658
☑	607	1051.5284	2101.0423
☑	608	1057.5249	2113.0352
☑	609	1057.5462	2113.0779
☑	610	1058.5169	2115.0193
☑	611	1058.5262	2115.0379
☑	612	1058.5443	2115.0740
☑	613	1065.5269	2129.0393
☑	614	1073.5369	2145.0593
☑	615	1078.5158	2155.0171
☑	616	1079.0313	2156.0480
☑	617	1079.0322	2156.0498
☑	618	1084.4427	2166.8709
☑	619	724.3592	2170.0558
☑	620	1086.0369	2170.0592

☑	621	1086.0402	2170.0659
☑	622	1087.0287	2172.0428
☑	623	1087.0296	2172.0447
☑	624	1087.5284	2173.0423
☑	625	1087.5320	2173.0495
☑	626	1092.4431	2182.8716
☑	627	728.6321	2182.8746
☑	628	1092.4456	2182.8767
☑	629	1093.4675	2184.9204
☑	630	729.3576	2185.0509
☑	631	1094.0340	2186.0535
☑	632	1094.0369	2186.0593
☑	633	1094.5319	2187.0493
☑	634	1100.4554	2198.8963
☑	635	738.0097	2211.0073
☑	636	1116.5587	2231.1029
☑	637	1130.5518	2259.0890
☑	638	1136.5444	2271.0743
☑	639	1139.0406	2276.0667
☑	640	572.8142	2287.2275
☑	641	1145.5658	2289.1170
☑	642	573.8316	2291.2972
☑	643	1159.5814	2317.1482
☑	644	1160.0566	2318.0986
☑	645	1166.5581	2331.1016
☑	646	1167.5589	2333.1032
☑	647	1167.5630	2333.1114
☑	648	1168.0532	2334.0917
☑	649	1168.0668	2334.1190
☑	650	1169.5923	2337.1700
☑	651	1174.5645	2347.1144
☑	652	1174.5645	2347.1145
☑	653	1182.5914	2363.1682
☑	654	1183.8068	2365.5989
☑	655	593.8602	2371.4117
☑	656	1189.5874	2377.1602
☑	657	795.3300	2382.9680
☑	658	600.7595	2399.0087
☑	659	619.1008	2472.3741
☑	660	1237.6252	2473.2359
☑	661	866.4009	2596.1810
☑	662	653.3783	2609.4841
☑	663	989.1428	2964.4067
☑	664	911.6811	3642.6951
☑	665	645.8871	3869.2790

Search Parameters

```
Type of search         : MS/MS Ion Search
Enzyme                 : Trypsin
Variable modifications : Carbamidomethyl (C),Oxidation (M)
Mass values            : Monoisotopic
Protein Mass           : Unrestricted
Peptide Mass Tolerance : ± 50 ppm
Fragment Mass Tolerance: ± 0.1 Da
Max Missed Cleavages   : 1
Instrument type        : ESI-QUAD-TOF
Number of queries      : 665
```

Mascot: http://www.matrixscience.com/

S4 Fig MASCOT Search Results – Tris-Tricine Sample

MATRIX SCIENCE Mascot Search Results

User :
Email :
Search title : **Submitted from 14732 by Mascot Daemon on APAF-WS-08**
MS data file : **\\apaf-hpv-file\projects\External\e_17432_VicUniWell_SandiDempsey_20150209\1_MassSpec\5600\Run1\Results\150213_P17432_SD1096-2_1.MGF**
Database : **sp_sheep_140625 sheep_140625_ (647 sequences; 258179 residues)**
Timestamp : **13 Feb 2015 at 04:39:38 GMT**
Protein hits : **P60713** sp|P60713|ACTB_SHEEP Actin, cytoplasmic 1 OS=Ovis aries GN=ACTB PE=2 SV=1
O77727 sp|O77727|K1C15_SHEEP Keratin, type I cytoskeletal 15 OS=Ovis aries GN=KRT15 PE=2 SV=1
Q9TTE2 sp|Q9TTE2|PGS2_SHEEP Decorin OS=Ovis aries GN=DCN PE=2 SV=1
Q9BGM5 sp|Q9BGM5|K1C25_SHEEP Keratin, type I cytoskeletal 25 OS=Ovis aries GN=KRT25 PE=2 SV=1
P14639 sp|P14639|ALBU_SHEEP Serum albumin OS=Ovis aries GN=ALB PE=2 SV=1
P15241 sp|P15241|K2M2_SHEEP Keratin, type II microfibrillar, component 7C OS=Ovis aries PE=1 SV=1
P12436 sp|P12436|VP5_BTV1A Outer capsid protein VP5 OS=Bluetongue virus 1 (isolate Australia) GN=S6 PE=3 SV=1

	sp_sheep_140625	Decoy	False discovery rate
Peptide matches above identity threshold	22	4	18.18 %
Peptide matches above homology or identity threshold	22	4	18.18 %

Mascot Score Histogram

Ions score is -10*Log(P), where P is the probability that the observed match is a random event.
Individual ions scores > 11 indicate identity or extensive homology (p<0.05).
Protein scores are derived from ions scores as a non-probabilistic basis for ranking protein hits.

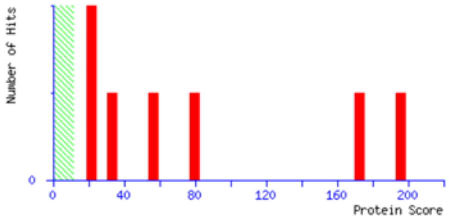

Peptide Summary Report

Format As Peptide Summary Help

Significance threshold p< 0.05 Max. number of hits AUTO Show Percolator scores

Standard scoring MudPIT scoring Ions score or expect cut-off 11 Show sub-sets 0

Show pop-ups Suppress pop-ups Sort unassigned Decreasing Score Require bold red

Select All Select None Search Selected **Error tolerant** Archive Report

1. P60713 **Mass:** 41710 **Score:** 196 **Matches:** 6(6) **Sequences:** 3(3) **emPAI:** 0.36
sp|P60713|ACTB_SHEEP Actin, cytoplasmic 1 OS=Ovis aries GN=ACTB PE=2 SV=1
Check to include this hit in error tolerant search or archive report

	Query	Observed	Mr(expt)	Mr(calc)	ppm	Miss	Score	Expect	Rank	Unique	Peptide
☑	51	398.2400	794.4655	794.4650	0.65	0	34	0.00041	1	U	K.IIAPPER.K
☑	270	566.7674	1131.5203	1131.5197	0.60	0	(42)	6.9e-005	1	U	R.GYSFTTTAER.E
☑	271	566.7677	1131.5208	1131.5197	1.06	0	43	4.7e-005	1	U	R.GYSFTTTAER.E
☑	277	581.3128	1160.6109	1160.6111	-0.12	0	(40)	0.00022	1	U	K.EITALAPSTMK.I
☑	278	581.3129	1160.6113	1160.6111	0.18	0	(44)	0.00011	1	U	K.EITALAPSTMK.I
☑	284	589.3095	1176.6045	1176.6060	-1.30	0	68	5.4e-007	1	U	K.EITALAPSTMK.I + Oxidation (M)

2. O77727 **Mass:** 48740 **Score:** 174 **Matches:** 6(6) **Sequences:** 2(2) **emPAI:** 0.14
sp|O77727|K1C15_SHEEP Keratin, type I cytoskeletal 15 OS=Ovis aries GN=KRT15 PE=2 SV=1
Check to include this hit in error tolerant search or archive report

	Query	Observed	Mr(expt)	Mr(calc)	ppm	Miss	Score	Expect	Rank	Unique	Peptide
☑	56	404.2031	806.3916	806.3923	-0.86	0	(38)	0.00015	1	U	R.LAADDFR.L
☑	57	404.2036	806.3926	806.3923	0.43	0	48	1.9e-005	1	U	R.LAADDFR.L
☑	58	404.2073	806.4000	806.3923	9.66	0	(27)	0.0081	1	U	R.LAADDFR.L
☑	241	516.2998	1030.5851	1030.5910	-5.70	0	(27)	0.0034	1	U	R.VLDELTLTK.T
☑	242	516.3029	1030.5913	1030.5910	0.26	0	71	7.3e-008	1	U	R.VLDELTLTK.T
☑	243	516.3036	1030.5926	1030.5910	1.52	0	(34)	0.00043	1	U	R.VLDELTLTK.T

3. Q9TTE2 **Mass:** 39947 **Score:** 84 **Matches:** 2(2) **Sequences:** 1(1) **emPAI:** 0.08
sp|Q9TTE2|PGS2_SHEEP Decorin OS=Ovis aries GN=DCN PE=2 SV=1
Check to include this hit in error tolerant search or archive report

	Query	Observed	Mr(expt)	Mr(calc)	ppm	Miss	Score	Expect	Rank	Unique	Peptide
☑	264	550.3297	1098.6448	1098.6437	1.02	0	(42)	6.3e-005	1	U	K.ISPGAFAPLVK.L
☑	265	550.3300	1098.6454	1098.6437	1.53	0	55	3.2e-006	1	U	K.ISPGAFAPLVK.L

4. Q9BGM5 **Mass:** 49282 **Score:** 54 **Matches:** 4(3) **Sequences:** 2(2) **emPAI:** 0.14
sp|Q9BGM5|K1C25_SHEEP Keratin, type I cytoskeletal 25 OS=Ovis aries GN=KRT25 PE=2 SV=1
Check to include this hit in error tolerant search or archive report

	Query	Observed	Mr(expt)	Mr(calc)	ppm	Miss	Score	Expect	Rank	Unique	Peptide
	241	516.2998	1030.5851	1030.5845	0.63	0	(11)	0.12	2	U	R.VLDVITLCR.T
	242	516.3029	1030.5913	1030.5845	6.58	0	(13)	0.047	2	U	R.VLDVITLCR.T
	243	516.3036	1030.5926	1030.5845	7.85	0	14	0.041	2	U	R.VLDVITLCR.T
☑	263	545.7676	1089.5207	1089.5237	-2.71	0	53	4.8e-006	1	U	K.VTMQNLNDR.L

5. P14639 **Score:** 28 **Matches:** 1(1) **Sequences:** 1(1) **emPAI:** 0.05
sp|P14639|ALBU_SHEEP Serum albumin OS=Ovis aries GN=ALB PE=2 SV=1
Check to include this hit in error tolerant search or archive report

Query	Observed	Mr(expt)	Mr(calc)	ppm	Miss	Score	Expect	Rank	Unique	Peptide
50	395.2396	788.4647	788.4644	0.48	0	28	0.0015	1	U	K.IVTDLTK.V

6. P15241 **Mass:** 53647 **Score:** 22 **Matches:** 1(1) **Sequences:** 1(1) **emPAI:** 0.06
sp|P15241|K2M2_SHEEP Keratin, type II microfibrillar, component 7C OS=Ovis aries PE=1 SV=1
Check to include this hit in error tolerant search or archive report

Query	Observed	Mr(expt)	Mr(calc)	ppm	Miss	Score	Expect	Rank	Unique	Peptide
303	632.3513	1262.6880	1262.6870	0.81	0	22	0.0069	1	U	K.LGLDIEIATYR.R

Proteins matching the same set of peptides:
P25691 **Mass:** 55220 **Score:** 22 **Matches:** 1(1) **Sequences:** 1(1)
sp|P25691|K2M3_SHEEP Keratin, type II microfibrillar, component 5 OS=Ovis aries PE=1 SV=1

7. P12436 **Mass:** 59207 **Score:** 22 **Matches:** 6(5) **Sequences:** 1(1) **emPAI:** 0.06
sp|P12436|VP5_BTV1A Outer capsid protein VP5 OS=Bluetongue virus 1 (isolate Australia) GN=S6 PE=3 SV=1
Check to include this hit in error tolerant search or archive report

Query	Observed	Mr(expt)	Mr(calc)	ppm	Miss	Score	Expect	Rank	Unique	Peptide
329	598.9439	1793.8098	1793.7924	9.72	0	(15)	0.033	1	U	R.DGMQEEAIQEIAGMTR.G + Oxidation (M)
330	598.9439	1793.8098	1793.7924	9.72	0	(15)	0.03	1	U	R.DGMQEEAIQEIAGMTR.G + Oxidation (M)
332	598.9442	1793.8107	1793.7924	10.2	0	(11)	0.076	1	U	R.DGMQEEAIQEIAGMTR.G + Oxidation (M)
333	598.9444	1793.8113	1793.7924	10.5	0	15	0.029	1	U	R.DGMQEEAIQEIAGMTR.G + Oxidation (M)
334	598.9444	1793.8113	1793.7924	10.5	0	(14)	0.038	1	U	R.DGMQEEAIQEIAGMTR.G + Oxidation (M)
335	598.9444	1793.8113	1793.7924	10.5	0	(14)	0.039	1	U	R.DGMQEEAIQEIAGMTR.G + Oxidation (M)

Peptide matches not assigned to protein hits: (no details means no match)

Query	Observed	Mr(expt)	Mr(calc)	ppm	Miss	Score	Expect	Rank	Unique	Peptide
131	442.7633	883.5121	883.5491	-41.85	0	9	0.13	1		IITPAITR
328	897.9110	1793.8075	1793.7924	8.43	0	8	0.16	1		DGMQEEAIQEIAGMTR + Oxidation (M)
331	598.9439	1793.8098	1793.7924	9.72	0	8	0.17	1		DGMQEEAIQEIAGMTR + Oxidation (M)
222	487.2698	972.5250	972.5175	7.75	1	7	0.18	1		NGAPMTRVK
217	481.2473	960.4801	960.4487	32.6	0	7	0.58	1		DLPQMWR + Oxidation (M)
181	457.2951	912.5756	912.5644	12.3	0	7	0.19	1		VLLSSPAVK
250	523.2846	1044.5546	1044.5862	-30.29	1	7	0.19	1		LSRLQCLR + Carbamidomethyl (C)
312	681.8625	1361.7104	1361.7554	-33.06	1	7	0.23	1		QKSQLILEEFK
327	897.9110	1793.8074	1793.7924	8.38	0	7	0.2	1		DGMQEEAIQEIAGMTR + Oxidation (M)
30	379.2113	756.4080	756.3952	16.9	0	6	0.53	1		MPPSGLR
276	576.2876	1150.5606	1150.5506	8.72	0	6	0.32	1		YLAEVAAGDDK
31	379.2168	756.4191	756.3952	31.6	0	6	0.57	1		MPPSGLR
115	435.7553	1738.9921	1738.9267	37.6	1	5	0.46	1		VVSDPQGIRAWVAWR
223	487.2707	972.5268	972.5175	9.60	1	5	0.57	1		NGAPMTRVK
53	400.7326	799.4507	799.4341	20.9	0	4	0.35	1		DLIHFR
306	643.8787	1285.7428	1285.7241	14.5	1	4	0.44	1		SKLAGLEEALQK
209	477.3051	952.5956	952.5705	26.4	0	3	0.49	1		LLEVLNPR
227	494.3017	986.5889	986.5509	38.5	1	3	0.53	1		TSLKTPGGAR
189	464.2524	926.4902	926.4722	19.4	1	3	1.1	1		YRAYQAR
55	403.2309	1206.6708	1206.6166	45.0	1	2	1.9	1		SEKMLLEDVK + Oxidation (M)
85	416.2503	830.4860	830.4610	30.1	0	2	1.5	1		SQLSQLR
207	472.5869	1414.7390	1414.7351	2.77	1	2	0.63	1		SSMSGLHLVKQGR + Oxidation (M)
282	583.2946	1164.5746	1164.5267	41.1	0	2	0.92	1		IEAMLECQR + Carbamidomethyl (C); Oxidation (M)
210	477.3053	952.5960	952.5705	26.7	0	2	0.67	1		LLEVLNPR
89	420.2481	838.4816	838.4548	31.9	0	1	0.73	1		KPSDPAPK
84	416.2407	1245.7002	1245.6904	7.87	1	0	3	1		QMIQVVKWAK + Oxidation (M)
204	472.2870	942.5594	942.5611	-1.70	1	0	0.91	1		GNKILVSGR
1	*350.1971*	*698.3796*								
2	*350.2004*	*698.3863*								
3	*350.2039*	*698.3933*								
4	*350.2056*	*698.3967*								
5	*354.0738*	*706.1331*								
6	*355.0709*	*708.1272*								
7	*355.1950*	*708.3755*								
8	*362.1218*	*722.2291*								
9	*362.1947*	*722.3748*								
10	*362.2214*	*722.4283*								
11	*362.2220*	*722.4294*								
12	*362.2226*	*722.4306*								
13	*362.2226*	*722.4307*								
14	*362.2229*	*722.4312*								
15	*364.2262*	*726.4379*								
16	*368.0537*	*734.0929*								
17	*368.0947*	*734.1748*								
18	*368.1012*	*734.1879*								
19	*369.1918*	*736.3690*								
20	*369.9351*	*737.8556*								
21	*370.7346*	*739.4547*								
22	*372.2014*	*742.3883*								
23	*372.2020*	*742.3893*								
24	*373.1478*	*744.2810*								
25	*374.0657*	*746.1168*								
26	*376.0835*	*750.1524*								
27	*377.0885*	*752.1624*								
28	*377.8027*	*753.5909*								
29	*378.1180*	*754.2215*								
32	*381.2295*	*760.4444*								
33	*381.2297*	*760.4449*								

☑	34	384.2344	766.4543
☑	35	384.2358	766.4571
☑	36	384.2360	766.4574
☑	37	384.2368	766.4591
☑	38	384.2376	766.4607
☑	39	386.1001	770.1856
☑	40	386.1069	770.1993
☑	41	388.1730	774.3313
☑	42	388.1958	774.3770
☑	43	391.2236	780.4326
☑	44	391.2830	780.5513
☑	45	391.2837	780.5529
☑	46	391.2838	780.5531
☑	47	391.2849	780.5552
☑	48	392.2408	782.4670
☑	49	392.7470	783.4793
☑	52	400.6154	799.2162
☑	54	401.2611	800.5077
☑	59	405.1381	808.2617
☑	60	405.2235	808.4324
☑	61	405.2243	808.4341
☑	62	406.2465	810.4784
☑	63	406.2482	810.4818
☑	64	406.2486	810.4826
☑	65	406.2486	810.4826
☑	66	407.1495	812.2845
☑	67	408.2405	814.4665
☑	68	411.8111	821.6076
☑	69	411.9217	821.8288
☑	70	411.9229	821.8313
☑	71	411.9239	821.8332
☑	72	412.0831	822.1516
☑	73	412.2282	822.4419
☑	74	412.7521	823.4896
☑	75	412.7524	823.4902
☑	76	412.7531	823.4917
☑	77	413.2269	824.4393
☑	78	414.0972	826.1798
☑	79	414.0998	826.1851
☑	80	414.2141	826.4136
☑	81	414.2200	826.4254
☑	82	414.7623	827.5101
☑	83	416.1078	830.2010
☑	86	416.2508	830.4870
☑	87	416.2513	830.4881
☑	88	416.7479	831.4813
☑	90	421.7569	841.4992
☑	91	421.7575	841.5005
☑	92	421.7576	841.5006
☑	93	421.7577	841.5008
☑	94	421.7579	841.5013
☑	95	421.7582	841.5018
☑	96	421.7582	841.5019
☑	97	422.0508	842.0870
☑	98	425.3103	848.6060
☑	99	425.3109	848.6072
☑	100	426.2464	850.4783
☑	101	428.2618	854.5090
☑	102	428.2618	854.5090
☑	103	428.2619	854.5092
☑	104	428.2622	854.5098
☑	105	428.2622	854.5099
☑	106	428.2629	854.5113
☑	107	428.2638	854.5130
☑	108	428.7651	855.5157
☑	109	428.7654	855.5163
☑	110	428.7657	855.5169
☑	111	428.7665	855.5185
☑	112	433.7326	865.4505
☑	113	435.2474	868.4802
☑	114	435.7546	869.4947
☑	116	435.7557	869.4968
☑	117	435.7561	869.4977
☑	118	435.7670	869.5193
☑	119	435.7674	869.5202
☑	120	435.7707	869.5269
☑	121	435.7722	869.5299
☑	122	435.7727	869.5308
☑	123	436.7742	871.5339
☑	124	436.7743	871.5340
☑	125	437.2904	872.5662
☑	126	437.7516	873.4887
☑	127	440.1126	878.2106
☑	128	440.1141	878.2135
☑	129	442.0916	882.1686
☑	130	442.7625	883.5104
☑	132	442.7640	883.5134
☑	133	444.1069	886.1993
☑	134	445.2882	888.5619
☑	135	448.2127	894.4108
☑	136	448.2136	894.4126
☑	137	448.2144	894.4143
☑	138	449.7711	897.5276
☑	139	449.7714	897.5282

☑	140	449.7717	897.5287
☑	141	450.2754	898.5362
☑	142	450.2758	898.5371
☑	143	450.2778	898.5410
☑	144	450.2972	898.5799
☑	145	450.2998	898.5850
☑	146	450.7898	899.5651
☑	147	451.2650	900.5154
☑	148	451.2677	900.5207
☑	149	453.0727	904.1309
☑	150	453.1271	904.2397
☑	151	453.7797	905.5447
☑	152	453.7806	905.5467
☑	153	453.7812	905.5478
☑	154	453.7815	905.5485
☑	155	453.7821	905.5495
☑	156	453.7823	905.5500
☑	157	453.7825	905.5505
☑	158	453.7825	905.5505
☑	159	453.7831	905.5515
☑	160	453.7831	905.5517
☑	161	453.7835	905.5524
☑	162	453.7835	905.5525
☑	163	453.7836	905.5526
☑	164	453.7837	905.5528
☑	165	453.7838	905.5530
☑	166	453.7838	905.5530
☑	167	453.7839	905.5532
☑	168	453.7839	905.5533
☑	169	453.7840	905.5535
☑	170	453.7844	905.5543
☑	171	453.7845	905.5544
☑	172	453.7845	905.5544
☑	173	453.7845	905.5545
☑	174	453.7846	905.5546
☑	175	453.7846	905.5547
☑	176	453.7847	905.5548
☑	177	453.7848	905.5551
☑	178	453.7850	905.5555
☑	179	453.7860	905.5574
☑	180	456.1074	910.2002
☑	182	458.7880	915.5613
☑	183	458.7885	915.5624
☑	184	460.0934	918.1722
☑	185	461.7471	921.4797
☑	186	462.2119	922.4093
☑	187	462.2153	922.4160
☑	188	462.2645	922.5145
☑	190	464.2535	926.4925
☑	191	466.1705	930.3265
☑	192	468.2049	934.3952
☑	193	471.7911	941.5677
☑	194	471.7921	941.5697
☑	195	471.7934	941.5723
☑	196	471.7936	941.5727
☑	197	471.7937	941.5729
☑	198	471.7948	941.5750
☑	199	471.7948	941.5750
☑	200	471.7957	941.5768
☑	201	471.7962	941.5779
☑	202	471.7965	941.5784
☑	203	471.7967	941.5789
☑	205	472.2891	942.5637
☑	206	472.2894	942.5642
☑	208	476.2520	950.4894
☑	211	479.2758	956.5369
☑	212	479.2885	956.5625
☑	213	479.2894	956.5642
☑	214	480.8017	959.5889
☑	215	481.1367	960.2588
☑	216	481.1433	960.2720
☑	218	481.6353	961.2560
☑	219	484.1808	966.3470
☑	220	486.2873	970.5600
☑	221	486.2979	970.5813
☑	224	487.2740	972.5334
☑	225	490.1785	978.3425
☑	226	494.3014	986.5882
☑	228	494.3022	986.5898
☑	229	495.2924	988.5702
☑	230	495.2927	988.5709
☑	231	500.9528	999.8911
☑	232	501.7951	1001.5756
☑	233	502.8147	1003.6149
☑	234	505.2692	1008.5238
☑	235	505.2707	1008.5268
☑	236	511.3268	1020.6390
☑	237	513.2397	1024.4648
☑	238	515.2991	1028.5836
☑	239	515.7415	1029.4685
☑	240	515.7428	1029.4710
☑	244	516.3145	1030.6144
☑	245	516.3156	1030.6167
☑	246	516.8274	1031.6402

☑	247	517.2616	1032.5087
☑	248	517.2617	1032.5088
☑	249	519.7694	1037.5242
☑	251	523.8035	1045.5924
☑	252	524.8277	1047.6408
☑	253	528.7766	1055.5386
☑	254	530.2932	1058.5719
☑	255	533.2640	1064.5134
☑	256	533.3367	1064.6589
☑	257	356.2051	1065.5933
☑	258	357.2253	1068.6540
☑	259	536.3235	1070.6325
☑	260	538.3261	1074.6377
☑	261	538.3275	1074.6404
☑	262	538.8079	1075.6013
☑	266	554.2749	1106.5351
☑	267	369.9290	1106.7653
☑	268	560.3374	1118.6603
☑	269	560.3414	1118.6682
☑	272	570.3486	1138.6827
☑	273	571.3530	1140.6914
☑	274	571.3555	1140.6965
☑	275	571.3560	1140.6974
☑	279	582.1748	1162.3349
☑	280	582.3200	1162.6254
☑	281	582.8387	1163.6629
☑	283	586.8410	1171.6675
☑	285	590.3038	1178.5931
☑	286	590.3044	1178.5942
☑	287	590.3166	1178.6187
☑	288	590.3168	1178.6190
☑	289	590.8138	1179.6130
☑	290	597.3099	1192.6053
☑	291	597.3352	1192.6558
☑	292	608.8585	1215.7025
☑	293	406.7546	1217.2420
☑	294	410.2928	1227.8567
☑	295	615.3668	1228.7190
☑	296	411.9373	1232.7900
☑	297	412.2312	1233.6717
☑	298	412.2314	1233.6725
☑	299	416.2970	1245.8691
☑	300	628.3070	1254.5993
☑	301	628.8007	1255.5868
☑	302	421.7647	1262.2722
☑	304	635.3645	1268.7143
☑	305	639.3617	1276.7087
☑	307	430.2293	1287.6661
☑	308	651.8617	1301.7089
☑	309	661.8581	1321.7016
☑	310	442.5891	1324.7455
☑	311	442.5903	1324.7491
☑	313	465.2496	1392.7271
☑	314	356.0277	1420.0818
☑	315	478.9282	1433.7629
☑	316	725.9364	1449.8582
☑	317	738.3971	1474.7797
☑	318	738.3972	1474.7798
☑	319	498.5838	1492.7295
☑	320	381.0246	1520.0692
☑	321	769.3241	1536.6336
☑	322	547.3182	1638.9327
☑	323	547.3186	1638.9339
☑	324	422.9514	1687.7764
☑	325	425.0065	1695.9970
☑	326	439.8102	1755.2116
☑	336	449.7683	1795.0440
☑	337	456.5015	1821.9768
☑	338	492.8200	1967.2509
☑	339	512.0359	2044.1147
☑	340	514.0896	2052.3291
☑	341	428.0798	2562.4349
☑	342	485.8102	2908.8172

Search Parameters

```
Type of search         : MS/MS Ion Search
Enzyme                 : Trypsin
Variable modifications : Carbamidomethyl (C),Oxidation (M)
Mass values            : Monoisotopic
Protein Mass           : Unrestricted
Peptide Mass Tolerance : ± 50 ppm
Fragment Mass Tolerance: ± 0.6 Da
Max Missed Cleavages   : 1
Instrument type        : ESI-QUAD-TOF
Number of queries      : 342
```

Mascot: http://www.matrixscience.com/
