# Supplementary material for: A novel chemotactic factor derived from the extracellular matrix protein decorin recruits mesenchymal stromal cells in vitro and in vivo
Source: PLoS One. 2020 Jul 13;15(7):e0235784. doi: 10.1371/journal.pone.0235784 (PMC7357784; doi:10.1371/journal.pone.0235784)
Supplement: S2 File — Uncropped SDS-PAGE gels used in this manuscript. (PDF) [file pone.0235784.s006.pdf]

S6 Fig: Raw Image 1. Tris-Glycine SDS-PAGE

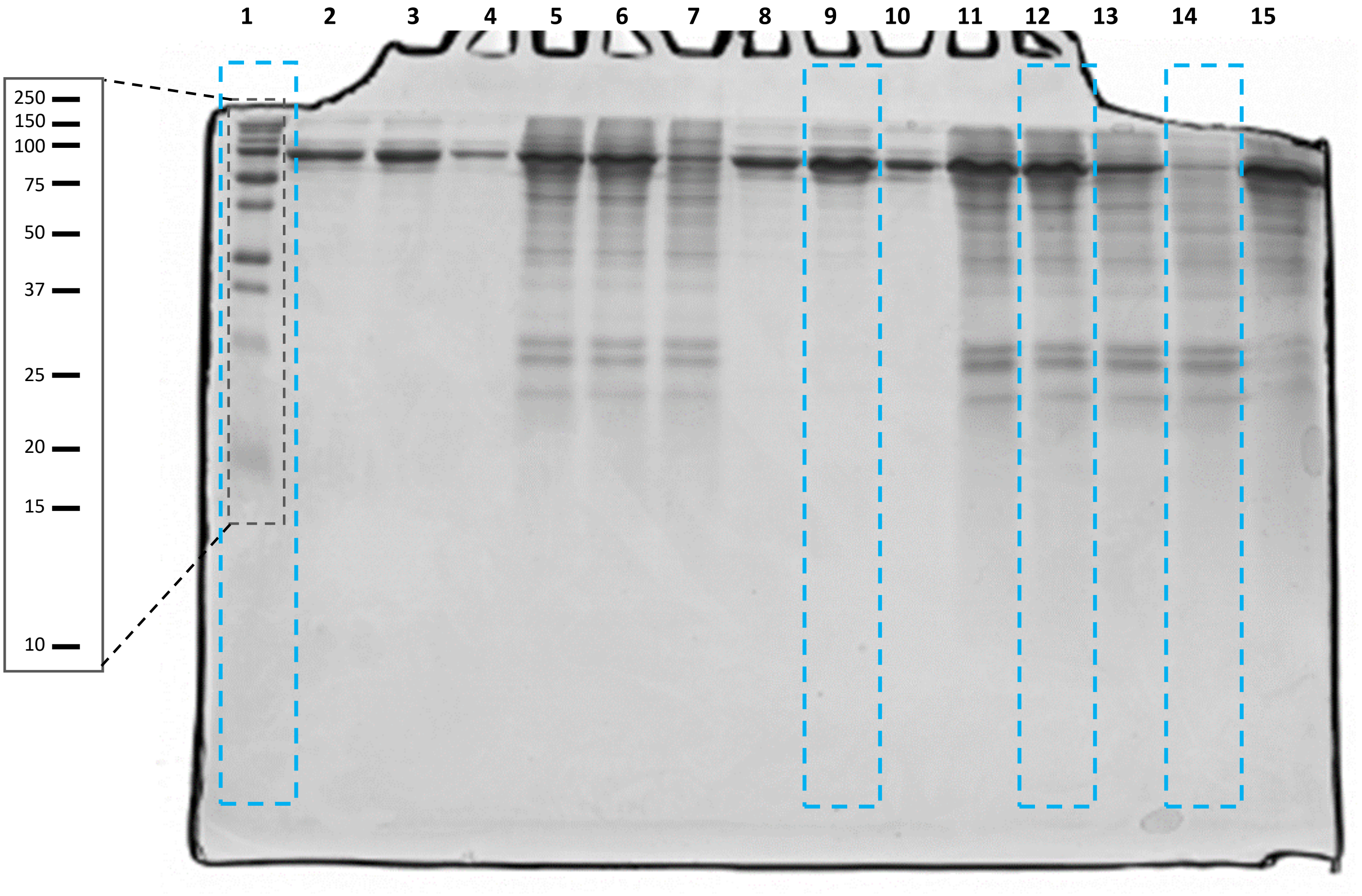

Experimental: Samples of conditioned media (30 µL) were diluted 3:1 with 4x Laemlie buffer. Tris-glycine gels (4% acrylamide stacking gel and a 20% acrylamide resolving gel) were made with a BioRad gel system and run with a glycine running buffer (25 mM Tris, 192 mM glycine, 0.1% w/v SDS, pH 8.3) (Sigma-Aldrich). A total volume of 15 µL of each sample was loaded per well and 8 µL of protein standard ladder (Precision Plus Protein™ Dual Color Standards, BioRad, Hercules, CA, United States). Tris-glycine gels were run for 1 h at 100 V. Gel was stained with Coomassie.

Lane	Sample/Comment	Lane	Sample/Comment
1	Marker	9	*OFM_{FITC10} (48 h incubation)
2	OFM (24 h incubation)	10	OFM_{FITC30} (48 h incubation)
3	OFM_{FITC10} (24 h incubation)	11	Mϕ+OFM (48 h incubation)
4	OFM_{FITC30} (24 h incubation)	12	*Mϕ+OFM_{FITC10} (48 h incubation)
5	Mϕ+OFM (24 h incubation)	13	Mϕ+OFM_{FITC30} (48 h incubation)
6	Mϕ+OFM_{FITC10} (24 h incubation)	14	*Mϕ (48 h incubation)
7	Mϕ+OFM_{FITC30} (24 h incubation)	15	OFM (48 h incubation)
8	OFM (48 h incubation)	* - denotes lanes used in Fig2.A (dashed blue line).	

S6 Fig: Raw Image 2. Tris-Glycine SDS-PAGE

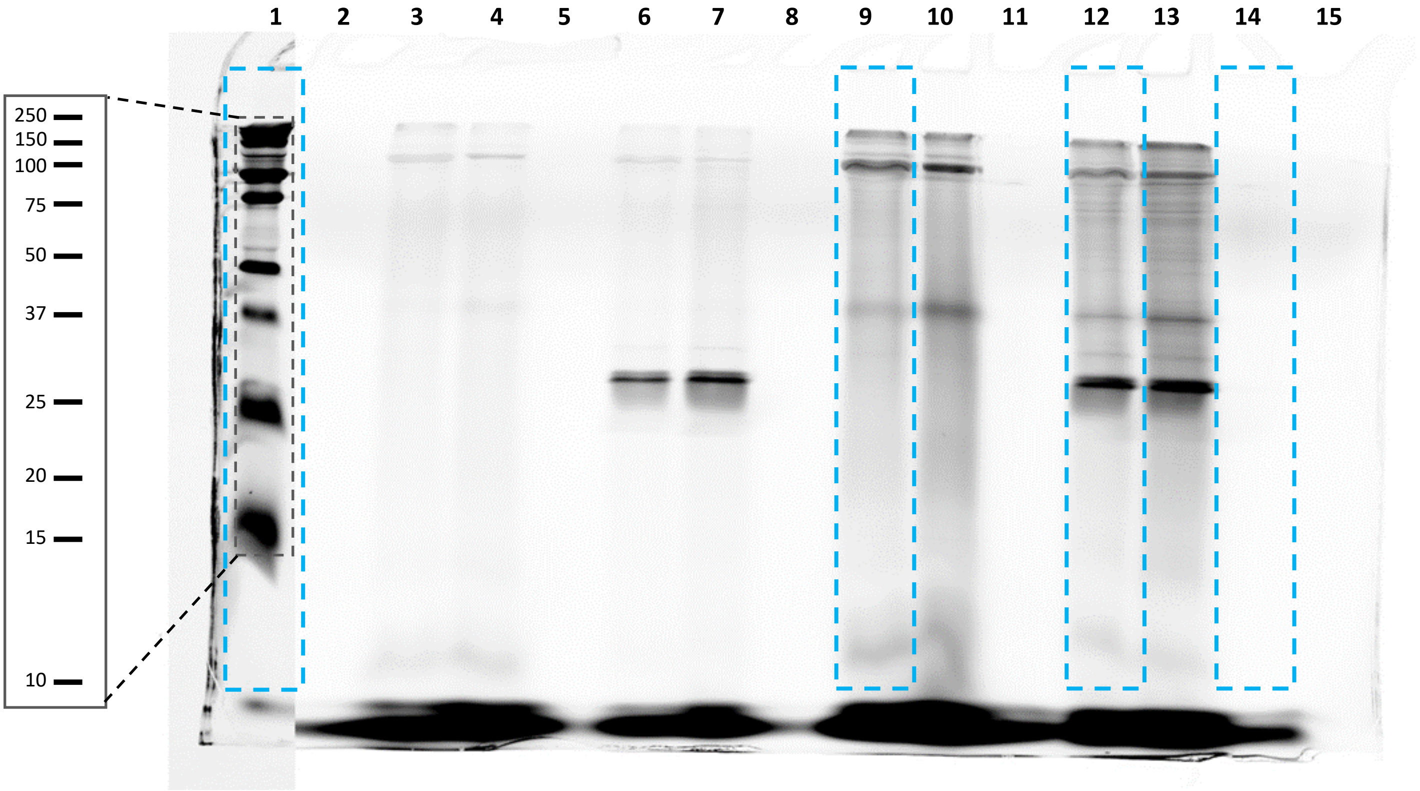

Experimental: Experimental: Samples of conditioned media (30 µL) were diluted 3:1 with 4x Laemlie buffer. Tris-glycine gels (4% acrylamide stacking gel and a 20% acrylamide resolving gel) were made with a BioRad gel system and run with a glycine running buffer (25 mM Tris, 192 mM glycine, 0.1% w/v SDS, pH 8.3) (Sigma-Aldrich). A total volume of 15 µL of each sample was loaded per well and 8 µL of protein standard ladder (Precision Plus Protein™ Dual Color Standards, BioRad, Hercules, CA, United States). Tris-glycine gels were run for 1 h at 100 V. Gel was imaged using a Fluoroskan Ascent FL (Thermo Fisher Scientific). Using red and FITC channels			
Lane	Sample/Comment	Lane	Sample/Comment
1	Marker	9	*OFM_{FITC10} (48 h incubation)
2	OFM (24 h incubation)	10	OFM_{FITC30} (48 h incubation)
3	OFM_{FITC10} (24 h incubation)	11	Mϕ+OFM (48 h incubation)
4	OFM_{FITC30} (24 h incubation)	12	*Mϕ+OFM_{FITC10} (48 h incubation)
5	Mϕ+OFM (24 h incubation)	13	Mϕ+OFM_{FITC30} (48 h incubation)
6	Mϕ+OFM_{FITC10} (24 h incubation)	14	*Mϕ (48 h incubation)
7	Mϕ+OFM_{FITC30} (24 h incubation)	15	OFM (48 h incubation)
8	OFM (48 h incubation)	* - denotes lanes used in Fig2.B (dashed blue line).	

S6 Fig: Raw Image 3. Tris-Tricine SDS-PAGE

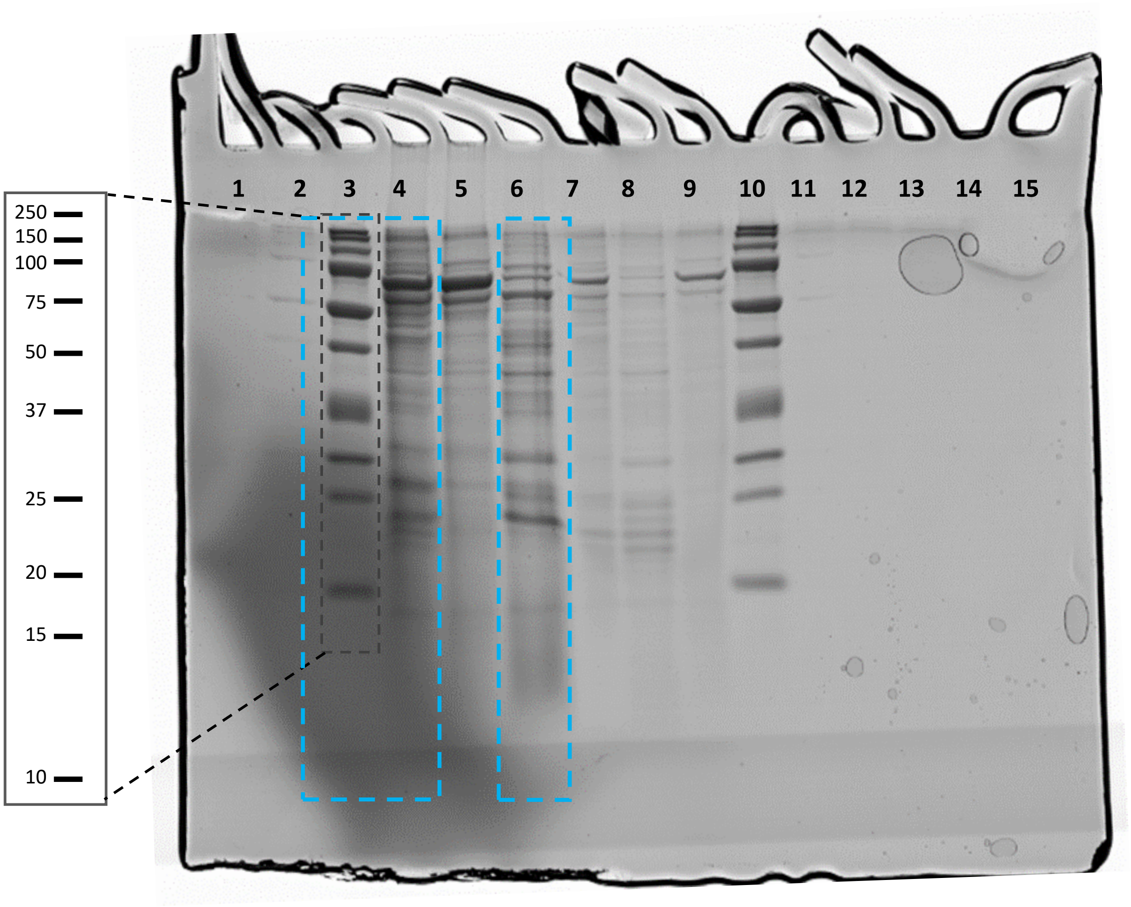

Experimental: Samples were diluted 3:1 with 4x Laemlie buffer. A total volume of 15 µL of each sample was loaded per well and 8 µL of protein standard ladder (Precision Plus Protein™ Dual Color Standards, BioRad). Tris-Tricine gels (4% acrylamide stacking gel and a 16% acrylamide resolving gel) were run using a cathode buffer (100 mM Tris, 100 mM tricine, 0.1% w/v SDS, pH 8.25) and an anode buffer (100 mM Tris, pH 8.9). Gels were run for 2 h at 60 V on ice (~4 °C). Gel was stained with Coomassie.

Lane	Sample/Comment	Lane	Sample/Comment
1	X, empty	9	Mϕ (1/10 dilution)
2	X, empty	10	Marker
3	*Marker	11	X, empty
4	*Mϕ+OFM_{FITC10} (neat)	12	X, empty
5	Mϕ+OFM (neat)	13	X, empty
6	*Mϕ (neat)	14	X, empty
7	Mϕ+OFM_{FITC10} (1/10 dilution)	15	X, empty
8	Mϕ+OFM (1/10 dilution)	* - denotes lanes used in Fig3.A (dashed blue line).	

S6 Fig: Raw Image 4. Tris-Tricine SDS-PAGE

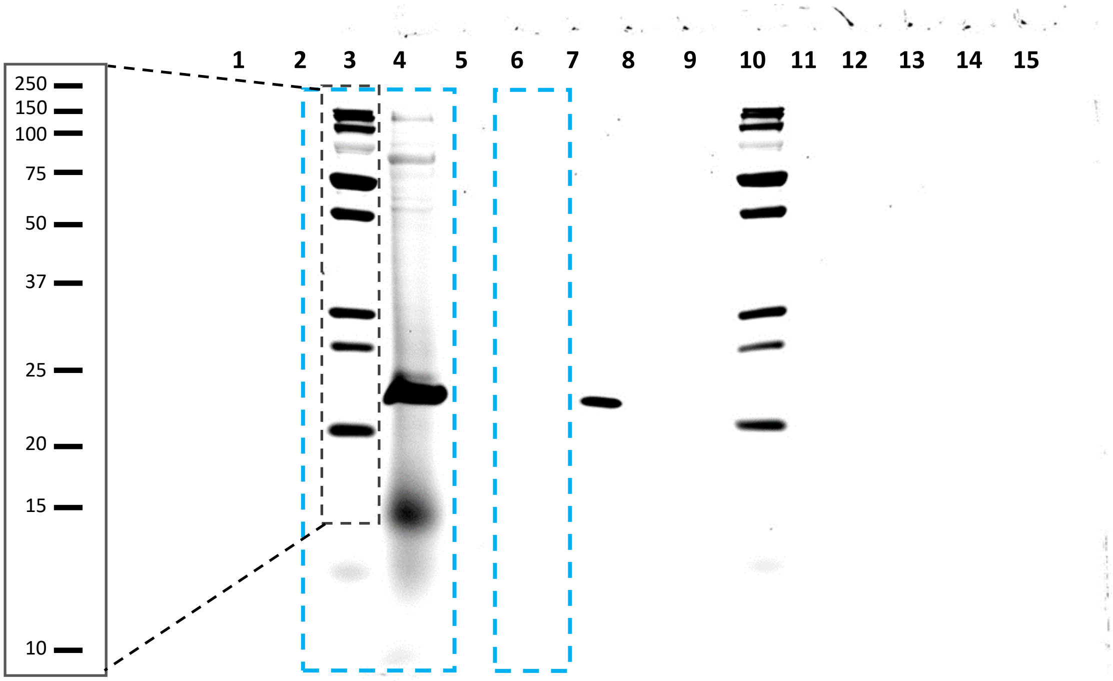

Experimental: Samples were diluted 3:1 with 4x Laemlie buffer. A total volume of 15 µL of each sample was loaded per well and 8 µL of protein standard ladder (Precision Plus Protein™ Dual Color Standards, BioRad). Tris-Tricine gels (4% acrylamide stacking gel and a 16% acrylamide resolving gel) were run using a cathode buffer (100 mM Tris, 100 mM tricine, 0.1% w/v SDS, pH 8.25) and an anode buffer (100 mM Tris, pH 8.9). Gels were run for 2 h at 60 V on ice (~4 °C). Gel was imaged using a Fluoroskan Ascent FL (Thermo Fisher Scientific).			
Lane	Sample/Comment	Lane	Sample/Comment
1	X, empty	9	Mϕ (1/10 dilution)
2	X, empty	10	Marker
3	*Marker	11	X, empty
4	*Mϕ+OFM$_{FITC10}$ (neat)	12	X, empty
5	Mϕ+OFM (neat)	13	X, empty
6	*Mϕ (neat)	14	X, empty
7	Mϕ+OFM$_{FITC10}$ (1/10 dilution)	15	X, empty
8	Mϕ+OFM (1/10 dilution)	* - denotes lanes used in Fig3.B (dashed blue line).	

S6 Fig: Raw Image 5. Bis-Tris SDS-PAGE

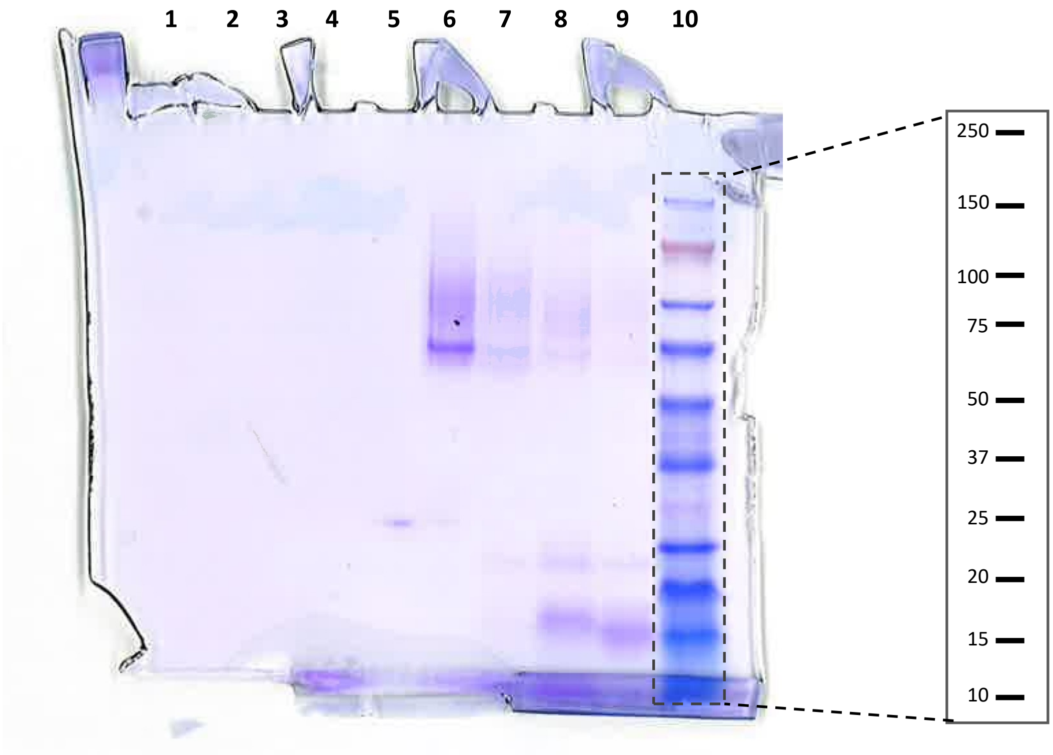

Experimental: Samples were diluted 3:1 with 4x Laemlie buffer and denatured, as described above. A total volume of 30 mL was loaded onto precast Bis-Tris gels (4-12% Bolt NuPAGE, Invitrogen, Carlsbad, CA, USA). Bis-Tris gels were run with a protein standard solution (5 mL) (SeeBlue protein standard, Invitrogen). Gels were run using an Invitrogen Mini Gel system (Invitrogen™) in a BOLT running buffer (Bolt™ MES SDS Running Buffer, Invitrogen™) for 90 mins at 100 V. Gels were rinsed (3x, ROH_2O, 10 mL) and then stained with Coomassie.

Lane	Sample/Comment	Lane	Sample/Comment
1	X, empty	9	DCN (0.25 ug + MMP12 (0.25 μg)
2	X, empty	10	Marker
3	X, empty		
4	X, empty		
5	MMP12 (2.5 μg)		
6	DCN (2.5 μg)		
7	DCN (0.25 ug + MMP12 (0.0025 μg)		
8	DCN (0.25 ug + MMP12 (0.125 μg)		
